# Plastome structure of 8 *Calanthe* s.l. species (Orchidaceae): comparative genomics, phylogenetic analysis

**DOI:** 10.1186/s12870-022-03736-0

**Published:** 2022-08-03

**Authors:** Consolata Nanjala, Vincent Okelo Wanga, Wyclif Odago, Elizabeth Syowai Mutinda, Emmanuel Nyongesa Waswa, Millicent Akinyi Oulo, Elijah Mbandi Mkala, Josiah Kuja, Jia-Xin Yang, Xiang Dong, Guang-Wan Hu, Qing-Feng Wang

**Affiliations:** 1 CAS Key Laboratory of Plant Germplasm Enhancement and Specialty Agriculture, Wuhan Botanical Garden, Chinese Academy of Sciences, Wuhan, 430074 China; 2 University of Chinese Academy of Sciences, Beijing, 100049 China; 3 Sino-Africa Joint Research Center, Chinese Academy of Sciences, Wuhan, 430074 China; 4 Department of Biology, University of Copenhagen, Copenhagen, Denmark

**Keywords:** *Calanthe*, Chloroplast genome, Genome comparison, *Phaius*, Phylogeny

## Abstract

**Background:**

*Calanthe* (Epidendroideae, Orchidaceae) is a pantropical genus distributed in Asia and Africa. Its species are of great importance in terms of economic, ornamental and medicinal values. However, due to limited and confusing delimitation characters, the taxonomy of the *Calanthe* alliance (*Calanthe*, *Cephalantheropsis*, and *Phaius*) has not been sufficiently resolved. Additionally, the limited genomic information has shown incongruences in its systematics and phylogeny. In this study, we used illumina platform sequencing, performed a *de novo* assembly, and did a comparative analysis of 8 *Calanthe *group species' plastomes: 6* Calanthe *and 2 *Phaius* species. Phylogenetic analyses were used to reconstruct the relationships of the species as well as with other species of the family Orchidaceae.

**Results:**

The complete plastomes of the *Calanthe* group species have a quadripartite structure with varied sizes ranging between 150,105bp-158,714bp, including a large single-copy region (LSC; 83,364bp- 87,450bp), a small single-copy region (SSC; 16,297bp -18,586bp), and a pair of inverted repeat regions (IRs; 25,222bp - 26,430bp). The overall GC content of these plastomes ranged between 36.6-36.9%. These plastomes encoded 131-134 differential genes, which included 85-88 protein-coding genes, 37-38 tRNA genes, and 8 rRNA genes. Comparative analysis showed no significant variations in terms of their sequences, gene content, gene order, sequence repeats and the GC content hence highly conserved. However, some genes were lost in *C*. *delavayi* (*P. delavayi*), including *ndhC*, *ndhF*, and *ndhK* genes. Compared to the coding regions, the non-coding regions had more sequence repeats hence important for species DNA barcoding. Phylogenetic analysis revealed a paraphyletic relationship in the *Calanthe* group, and confirmed the position of *Phaius delavayi* in the genus *Calanthe* as opposed to its previous placement in *Phaius*.

**Conclusion:**

This study provides a report on the complete plastomes of 6 *Calanthe* and 2 *Phaius* species and elucidates the structural characteristics of the plastomes. It also highlights the power of plastome data to resolve phylogenetic relationships and clarifies taxonomic disputes among closely related species to improve our understanding of their systematics and evolution. Furthermore, it also provides valuable genetic resources and a basis for studying evolutionary relationships and population genetics among orchid species.

**Supplementary Information:**

The online version contains supplementary material available at 10.1186/s12870-022-03736-0.

## Background

*Calanthe* is the largest genus in tribe Collabieae (Epidendroideae; Orchidaceae), with more than 220 species [1], distributed across tropical and subtropical Asia, Australia, Madagascar, Africa, Central and South America, and the Caribbean [2–4]. *Calanthe* species are evergreen or deciduous plants, terrestrial (rarely epipetric or epiphytic) with thick roots, small oval pseudobulbs, highly ridged leaves, and upright, occasionally arching flowering stems [5]. Their flowers arise from the basal leaf with showy, white, yellow, or pink colors with a resupinate opening, ranging from small, medium and large [6]. They often turn dark blue after damage or during senescence [7]. *Calanthe* is the first orchid species to be artificially used by humans for hybridization purposes [8]. Its species have numerous ornamental and medicinal values and were popular ornamental house plants during the Victorian era [9]. In traditional systems of medicine such as Chinese Traditional Medicine (TCM) and Indian Ayurveda, *Calanthe* has diverse uses, including detoxification and body cooling, resolving hard lumps, promoting blood circulation, treatment of arthritis, rheumatism, ulcers, common colds, and traumatic injuries. In addition, some species are used as tonics and as aphrodisiacs [10, 11].

*Calanthe* has undergone a series of intrageneric taxonomic revisions for many centuries since its establishment in 1821 [12]. The genus was first subdivided into two subgenera and various sections by Schlechter in 1914, and most authors have observed this subgeneric classification in the subsequent years in their studies [13]. Subgenus *Preptanthe* (Rchb.f.) Schltr. is characterized by swollen pseudobulbs and annual leaves, whereas subgenus *Calanthe* lacks prominent pseudobulbs and has evergreen leaves. The *Calanthe* group, a well-defined group of orchids in tribe Collabieae of subfamily Epidendroideae, was identified to include the genera *Calanthe* R. Br., *Cephalantheropsis* Guillaumin, and *Phaius* Lour. [14]. The three genera have been shown to have a close relationship hence leading to delimitation challenges, especially in the genera *Calanthe* and *Phaius*. Generally, species in this group are characterized by plicate leaves, simple, widely spreading sepals and petals, fused lip base and column, and eight waxy pollinia [6]. Morphologically, *Cephalantheropsis* is characterized by a spurless labellum, free from the column, and pollinia growing directly on the globose viscidium, while the *Phaius* labellum has a spur, grows at the column base but lacks adnation with column wings with pollinia attached by short caudicles. On the other hand, *Calanthe* is characterized by its labellum adnate to column wings forming a tube and spurred base having pollinia bound by conspicuous or inconspicuous caudicles, adherent to a sticky viscidium [15]. However, adnation of the lip to the column has been shown to have evolved several times independently, and some species, such as *Phaius delavayi* (Finet) P.J.Cribb & Perner, have an intermediate column type between these two states hence taxonomic incongruences [16].

In terms of molecular studies, the family Orchidaceae generally has been subjected to two classification systems (i.e., Dressler 1993 and Chase et al. (1994)) [17–21] that try to infer its phylogeny and evolution from genus to subfamily levels. Within the Subfamily Epidendroideae, three genera; *Calanthe*, *Cephalantheropsis* and *Phaius*, form an independent alliance known as the *Calanthe* alliance, which can be easily distinguished from other taxa within the subfamily [1, 4, 15]. However, the phylogenetic relationships and affinities within the *Calanthe* alliance remain unresolved. Previous molecular studies conducted on Epidendroideae treated two lineages of the traditional *Calanthe*, namely: *Preptanthe* Rchb. f. and *Styloglossum* Breda, as distinct genera [3, 5]. Additionally, other molecular studies on the *Calanthe* group reported that *Calanthe* is a polyphyletic genus that clusters with its relatives *Cephalantheropsis* and *Phaius, *forming an independent alliance within Epidendroideae (Orchidaceae) named the *Calanthe* alliance [3, 15, 16]*.* This alliance can be differentiated from other taxonomic groups within the family based on plicate leaves, similar sepals and petals, basal and lateral inflorescence, resupinate flowers with free sepals and petals, spurred lips, and eight waxy pollinia forming two groups [15]. However, determining the phylogenetic and taxonomic relationships within this alliance is difficult. This is due to the uncertainties in the precise delimitation characters and the incongruent molecular phylogeny within the *Calanthe* alliance, that has led to poor taxonomic classification; thus, the phylogenetic and taxonomic relationships within the *Calanthe* alliance remain inconclusive. To better understand their phylogenetic relationships, it is necessary for us to identify discrepancies in the genetic information of the major clade the *Calanthe* alliance.

Genus *Phaius* contains ca. 40 known species, out of which 9 occur in China [6, 21]. The species in this genus are also characterized by a labellum growing at the base of the column having a spur but lacks adnation to column wings, and a pollinium usually attached by short caudicles [15]. Based on morphological data, this genus is separated into two types: bract caducous or persistent [6]. *Calanthe*, on the other hand, is characterized by a labellum which is adnate to column wings forming a tube and spurred base, and pollinium having conspicuous or inconspicuous caudicles, usually adhering to a sticky viscidium [6, 14, 21]. Previously, the genera *Calanthe* and *Phaius* were distinguished by the lip being adnate to or almost free from the column, respectively, but all intermediate conditions exist [22]. However, there are limited genetic studies on these species, and the aforementioned characteristics are ambiguous, thus not sufficient to distinguish among the genera or infrageneric taxa of the *Calanthe* alliance. Therefore, there is a need for more in-depth studies to resolve these relationships.

Systematics and phylogeny, since its establishment, has promoted classification and interpretation of the evolutionary relationships among angiosperms via genomic analysis [23]. Chloroplasts are essential in photosynthesis and form part of the primary genetic system together with the nucleus and the mitochondria [24]. Plastome (Chloroplast genome) sizes range from 120 to 170 kb in many angiosperms [25]. The plastome is relatively conserved in terms of the size of the gene, gene content, arrangement of the genes, and genome structure [26]. Compared to the nuclear genome, the chloroplast genome undergoes very few nucleotide substitutions and gene rearrangements; hence has been a perfect model to study genetic change and phylogeny in complex terrestrial plants [27].

In the current study, we sequenced, assembled and annotated the complete chloroplast genome of 8 species from the two genera in the *Calanthe* alliance, namely: *Calanthe* and *Phaius*. The aim of this study was to; 1.) Understand the genetic structure and variation within the plastomes; 2.) Identify and describe the characteristics of the cp genome structure, sequence divergence, mutational hotspot regions, and repeat regions across the plastomes and, 3.) Evaluate the phylogenetic relationships between the genera *Calanthe* and *Phaius*, which may be useful for further species evolution studies.

## Results

### Chloroplast genome organization of the *Calanthe* group species

**Fig. 1:**
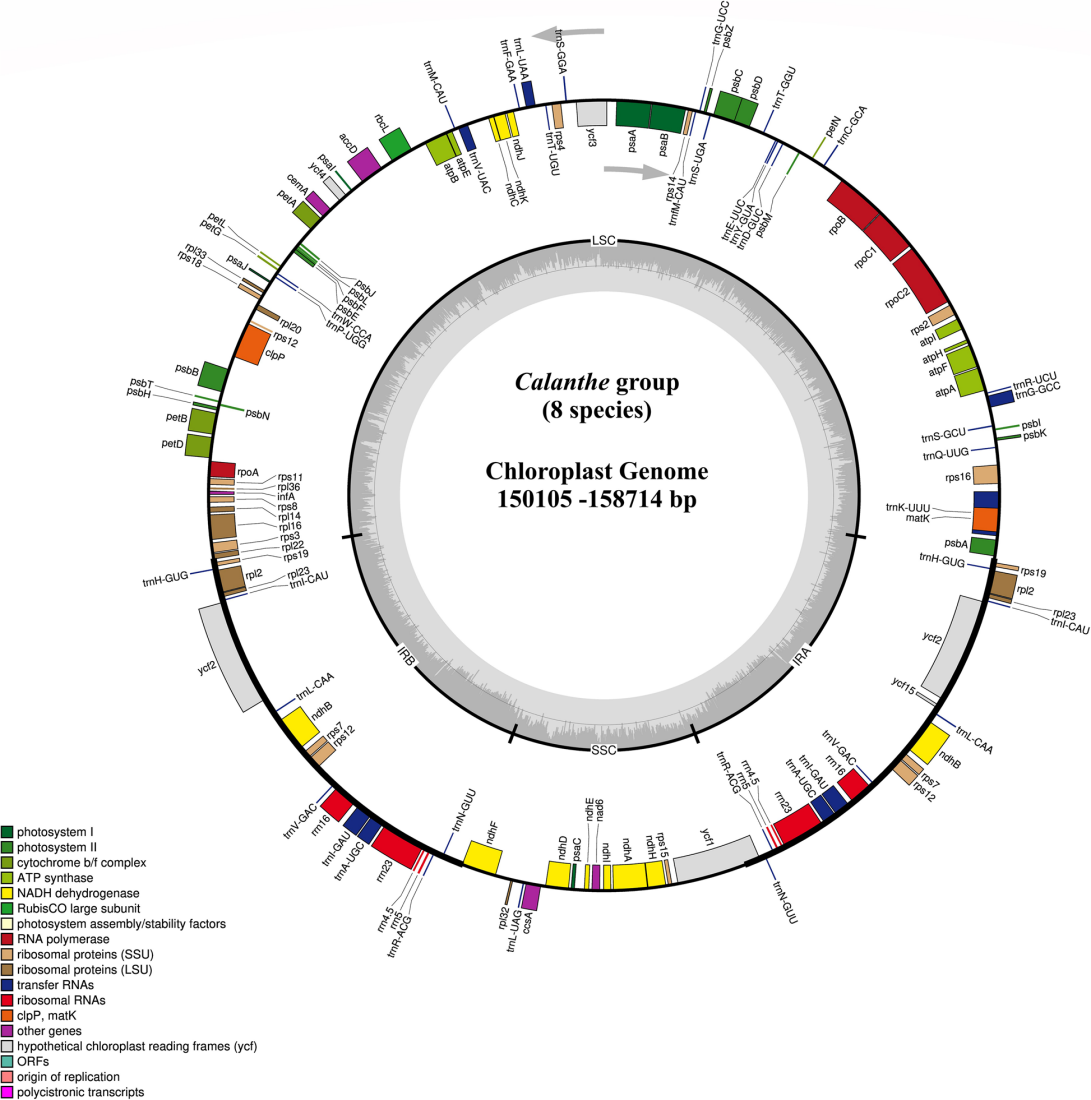
Gene map of the complete chloroplast genomes of 8 species of the *Calanthe* group. Annotated genes are colored according to functional categories whereby the genes outside the circle were transcribed clockwise, while the genes placed inside the circle were transcribed counterclockwise. The dark grey color in the inner circle represents GC content, whereas the light grey color corresponds to AT content

The complete chloroplast genomes of 8 species of the *Calanthe* group display a common quadripartite structure consisting of two Inverted Repeat (IR) regions (IRa and IRb), a Large Single Copy (LSC) region, and a Small Single Copy (SSC) region. Their sizes range as follows: IRs (25,222bp-26,430bp), LSC (83,364bp-87,450bp), and an SSC (16,297bp-18,586bp) (Fig. 1; Table 1).

**Table 1:** Basic features of the chloroplast genome of the 8 *Calanthe* group species

Species	*Calanthe alpina*	*Calanthe brevicornu*	*Calanthe ecarinata*	*Calanthe nipponica*	*Calanthe taibaishanensis*	*Calanthe tricarinata*	*Phaius delavayi*	*Phaius flavus*
**Accession no.**	OL322023	OL348396	OL348397	OL348398	OL351366	OL351367	OL351368	OL351369
**Total Length (bp)**	156591	158384	158329	158714	157959	158343	150105	158556
**LSC Size (bp)**	85489	87155	87293	87450	87129	87285	83364	87216
**LSC GC%**	34.3	34.3	34.3	34.2	34.2	34.3	34.5	34.6
**LSC Length (%)**	54.6	55.0	55.1	55.1	55.2	55.1	55.5	55.0
**SSC Size (bp)**	18436	18531	18500	18404	18424	18522	16297	18586
**SSC GC%**	29.5	29.6	29.7	29.7	29.6	29.7	29.4	29.8
**SSC Length (%)**	13.1	11.7	11.7	11.6	11.7	12.0	11.0	11.7
**IR Size (bp)**	26333	26349	26268	26430	26203	26268	25222	26377
**IR GC%**	43.1	43.1	43.1	43.0	43.1	43.1	43.3	43.0
**IR Length (%)**	16.2	16.6	16.6	16.7	16.6	16.4	16.8	16.6
**PCGs region size (bp)**	78678	79083	79083	79275	79251	79083	73473	79353
**tRNA size (bp)**	2870	2870	2870	2870	2870	2870	2870	2812
**rRNA size (bp)**	9042	9042	9042	9042	9042	9042	9042	9042
**GC content (%)**	36.7	36.7	36.7	36.6	36.6	36.7	36.9	36.8
**No. of PCGs**	88	88	88	88	88	88	85	88
**No. of tRNA**	38	38	38	38	38	38	38	37
**No. of rRNA**	8(4)	8(4)	8(4)	8(4)	8(4)	8(4)	8(4)	8(4)
**No. of genes**	134	134	134	134	134	134	131	133

The GC content was varied within the LSC, SSC, and IR regions, ranging between 34.2-34.6%, 29.4-29.8%, and 43.0-43.1%, respectively, in the regions (Table 1).

Most of the genomes encoded 134 differential genes, containing 88 CDS and 38 tRNA. However, only 131 and 133 genes, 85 and 88 CDs, and 38 and 37 tRNA genes were recorded in *P. delavayi* and *P. flavus*, respectively. All of these species encoded eight rRNA genes. The 8 *Calanthe* group plastomes had identical numbers, order, and names except for the three genes lost in *P. delavayi*, namely, *ndhC*, *ndhF*, and *ndhK.* In addition, the *Calanthe* group plastome contained 6 *tRNA* genes (*trnA-UGC*, *trnI-GAU*, *trnG-UCC*, *trnK-UUU*, *trnL-UAA* and *trnV-UAC*) and nine protein-coding genes (*rpl2*, *rpl16*, *rps16*, *rpoC1*, *ndhA*, *ndhB*, *atpF*, *petB* and *petD*) having one intron and three genes (*ycf3*, *clpP*, and *rps12*) containing two introns. A total of 19 genes were duplicated in the IR regions, including three types of genes, namely, seven coding genes (*rps12*, *rps19*, *rps7*, *rpl2*, *rpl23*, *ndhB*, *ycf2*), eight tRNA (*trnH-GUG*, *trnI-CAU*, *trnL-CAA*, *trnV-GAC*, *trnI-GAU*, *trnA-UGC*, *trnR-ACG*, *trnN-GUU*) genes and four rRNA (*rrn16*, *rrn23*, *rrn4.5*, *rrn5*) genes (Table 2). The *rps12* gene was trans-spliced*,* overlapping two regions in the cp genome whereby the 5′-end exon was found in the LSC region and the intron, 3′-end exon located in the IR region. Moreover, three pairs of genes, *trnK-UUU*/*matK*, *atpE*/*atpB*, and *psbD*/*psbC* had overlapping sequences.

**Table 2:** Group of genes encoded in the complete cp genome of the 8 *Calanthe* group species

Category for Genes	Group of Genes	Name of Genes
Self-replication	transfer RNAs	*trnK-UUU*^a^*, trnQ-UUG, trnS-GCU, trnG-GCC, trnR-UCU, trnC-GCA, trnD-GUC, trnY-GUA, trnE-UUC, trnT-GGU, trnS-UGA, trnG-UCC*^a^*, trnfM-CAU, trnS-GGA, trnT-UGU, trnL-UAA*^a^*, trnF-GAA, trnV-UAC*^a^, trnM-CAU, trnW-CCA, trnP-UGG, trnH-GUG*, trnI-CAU*, trnL-CAA*, trnV-GAC*, trnI-GAU^a^*, trnA-UGC^a^*, trnR-ACG*, trnN-GUU*, trnL-UAG
	ribosomal RNAs	rrn16*, rrn23*, rrn4.5*, rrn5*
	RNA polymerase	*rpoA, rpoB, rpoC1*^a^*, rpoC2*
	Small subunit of ribosomal proteins (SSU)	*rps11, rps12*^a^*, rps14, rps15, rps16^a^, rps18, rps19*, rps2, rps3, rps4, rps7*, rps8
	Large subunit of ribosomal proteins (LSU)	*rpl14, rpl16*^a^*, rpl2*^a^*, rpl20, rpl22, rpl23*, rpl32, rpl33, rpl36
Genes for photosynthesis	Subunits of NADH-dehydrogenase	*ndhA*^a^*, ndhB*^a^*, ndhC^b^*, ndhD, ndhE, ndhF*^b^*, ndhH, ndhI, ndhJ, ndhK*^b^
	Subunits of photosystem I	*psaA, psaB, psaC, psaI, psaJ*
	Subunits of photosystem II	*psbA, psbB, psbC, psbD, psbE, psbF, psbI, psbJ, psbK, psbL, psbM, psbN, psbT, psbZ,*
	Subunits of cytochrome b/f complex	*petA, petB*^a^*, petD*^a^*, petG, petL, petN*
	Subunits of ATP synthase	*atpA, atpB, atpE, atpF*^a^*, atpH, atpI*
	Large subunit of rubisco	*rbcL*
Other genes	Translational initiation factor	*infA*
	Protease	*clpP*^a^
	Maturase	*matK*
	Subunit of Acetyl-CoA-carboxylase	*accD*
	Envelope membrane protein	*cemA*
	C-type cytochrome synthesis gene	*ccsA*
Genes of unknown function	hypothetical chloroplast reading frames (*ycf*)	ycf1, ycf *, ycf3^a^*, ycf4, ycf15*

Note:

^a^Genes containing introns

^b^Genes lost in *P. delavayi*

^*^Duplicated genes

### Contraction and expansion of IR regions

**Fig. 2:**
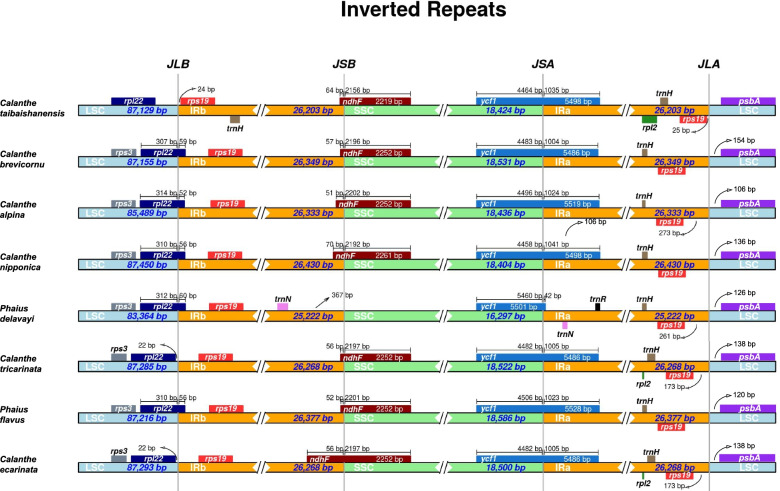
Comparison for border positions of LSC, SSC, and IR regions among the 8 *Calanthe* group species. The boxes denote genes, and the gap between the genes and the boundaries is indicated by the number of bases unless the gene coincides with the boundary. Extensions of genes are shown above the boxes

The chloroplast genome structure and the junction positions between IR regions among the eight species exhibited several structural variations in the LSC/IRb, IRb/SSC, SSC/IRa, and IRa/LSC borders (Fig. 2). Three different occurrences were observed in the LSC/IRb border. First, in *Calanthe ecarinata* and *C. tricarinata*, the *rpl22* gene was found in the LSC region 22bp away from the IRb region. Secondly, in 5 species, namely: *C. brevicornu*, *C. alpina*, *P. flavus*, *P. delavayi,* and *C. nipponica*, the *rpl22* gene overlapped in the LSC/IRb region by 52-60 bp in the IRb region. The third occurrence was observed in *C. taibaishanensis* whereby the *rps19* gene was 24 bp away from the LSC/IRb instead of *rpl22*. The IRb/SSC junction regions were relatively conserved in 7 species whereby the *ndhF* gene crossed over to the IRb region by 51-70 bp except in *P. delavayi* due to its *ndhF* gene loss. In this regard, the nearest gene *trnN* in IRb, was 367 bp away from the SSC region in *P. delavayi*. Both the SSC/IRa and IRa/LSC are well conserved among the 8 *Calanthe* group genomes whereby the *ycf1* gene crossed over the SSC/IRa boundary having 42-1035 bp into the IRa section. Furthermore, in the IRa/LSC junction of 7 species, the *psbA* gene is found in the LSC region, 106-154 bp away from the IRa. The IRa/LSC junction of *C. taibaishanensis* is distinct in that the *rps19* gene occurs in the IRa, 25 bp away from the LSC.

### Comparative genomic analysis

**Fig. 3:**
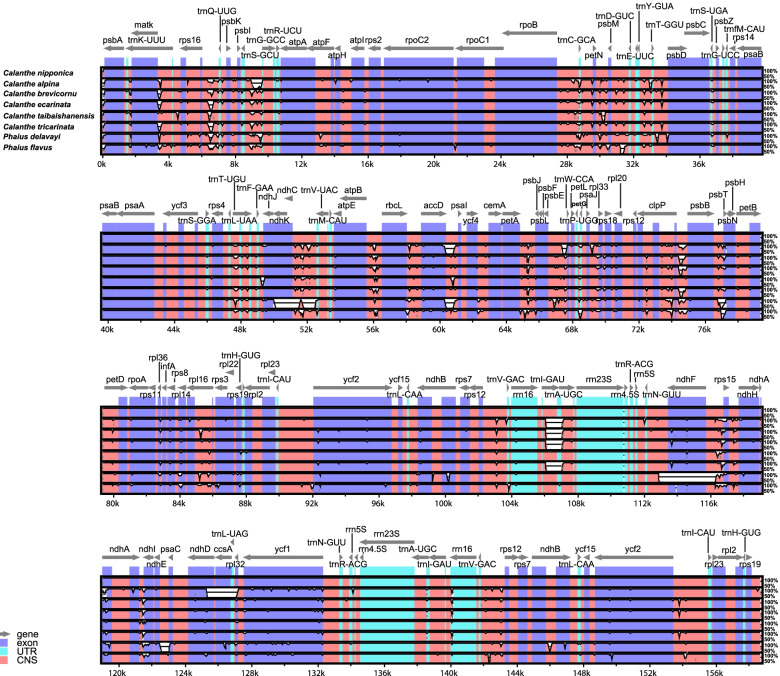
Global alignment of chloroplast genomes of the 8 *Calanthe* group species by mVISTA using *C. nipponica* as the reference. The top line shows the orientation of genes. A cut-off of 70% identity was used for the plots, and the Y-scale represents the percentage identity ranging from 50 to 100%

The mVISTA-based identity plot revealed the DNA sequence and gene synteny conservation across the eight plastomes and showed the regions with increased genetic variations (Fig. 3). The number, order, and orientation of genes were relatively conserved. Distinct sequence variations were recorded in several gene regions including *psbA*-*trnK-UUU*, *rps16*-*trnQ-UUG*, *matK-trnK-UUU-rps16*, *trnS-GCU-trnG*-*GCC*, *rpoB-trnC*-*GCA*, *petN-psbM*, *psbM-trnD*-*GUC*, *trnE*-*UUC-trnT*-*GGU*, *trnT*-*GGU*-*psbD*, *ndhK-trnM*-*CAU*, *atpB-rbcL*, *rbcL-accD*, *accD-psaI*, *petA-psbJ*, *psbE-petL*, *trnV*-*GAC-rps12*, *ccsA-ndhD*, *trnL*-*UAA*, *trnL*-*GAU*, *ndhF*, *ndhI*, *rps15*, *trnP-UGG*, *rpl33*, *clpP*, *psbT*, *rpl16*, *rpl14*, *rps8* and *rpl32.* Higher genetic variability was observed in the LSC and SSC regions than in the IR regions and in non-coding regions than in the conserved protein-coding regions. The rRNA genes were highly conserved with almost no variation in terms of their numbers among the plastomes. Moreover, greater variation was recorded in the IGS regions than those in the gene regions.

**Fig. 4:**
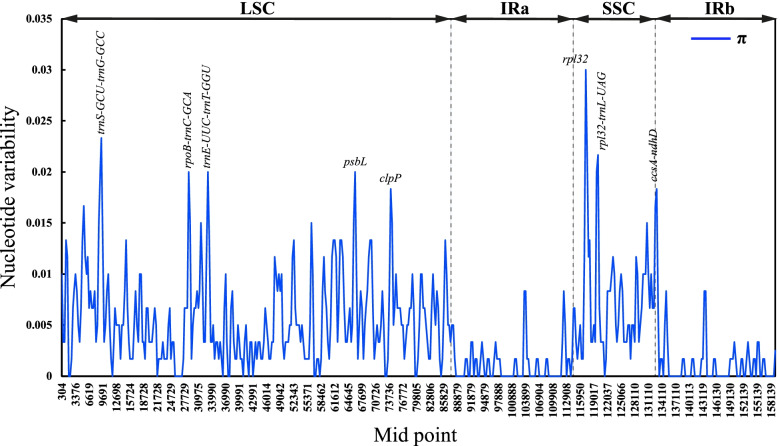
Comparative analysis of the nucleotide diversity values among the 8 *Calanthe* group chloroplast genomes. The X-axis represents the position of the midpoint of a window (kb), while the Y-axis indicates the nucleotide diversity of each window

The sliding window analysis identified three highly variable regions in the 8 *Calanthe* group plastomes with a nucleotide diversity (*Pi*) cut-off point set at *Pi* ≥0.03 (Fig. 4). The highly variable regions were mainly found in the LSC and SSC region compared to IR regions and in non-coding regions than coding regions. The highly variable regions were identified as follows; *trnS-GCU-trnG-GCC*, *rpoB-trnC-GCA*, *trnE-UCC-trnT-GGU*, *rpl32-trnL-UAG*, *ccsA-ndhD* and *psbL*, *clpP* and *rpl32* genes of the chloroplast genomes. These findings were consistent with the mVISTA results, whereby the variation in the IR regions of the chloroplast genomes was relatively lower than that in the LSC and SSC sections.

### Sequence repeats

**Fig. 5:**
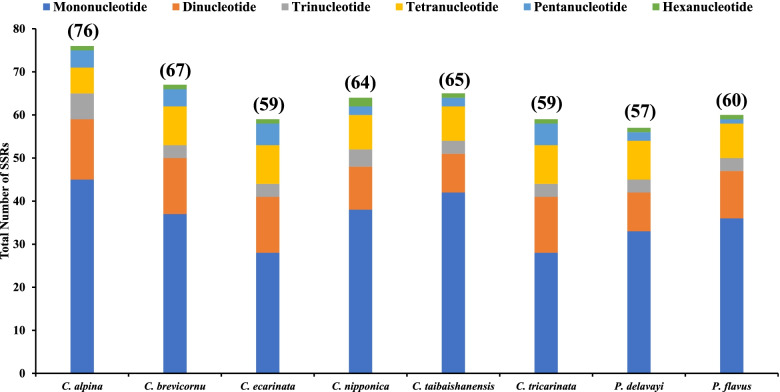
The total number of SSRs recorded in the cp genomes of 8 *Calanthe* group species

**Fig. 6:**
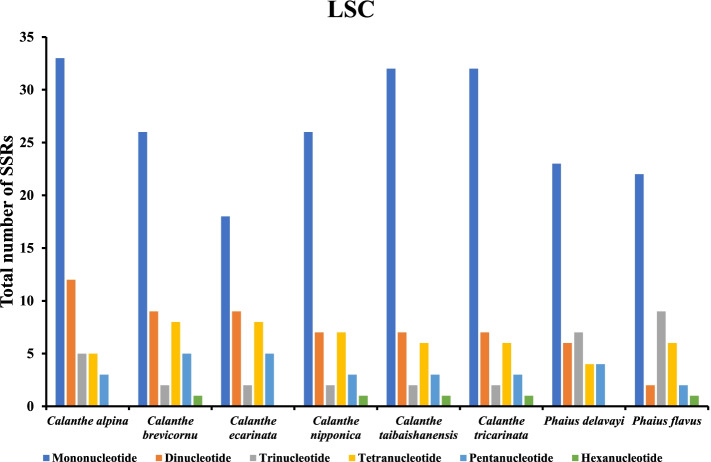
The total number of SSRs identified in the LSC regions of 8 *Calanthe* group species

**Fig. 7:**
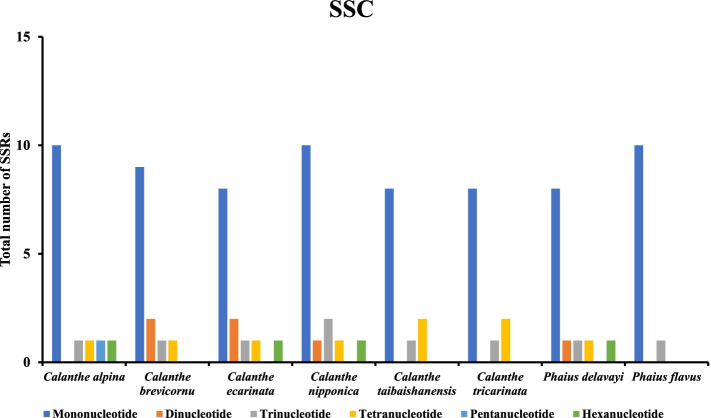
The total number of SSRs identified in the SSC regions of 8 *Calanthe* group species

**Fig. 8:**
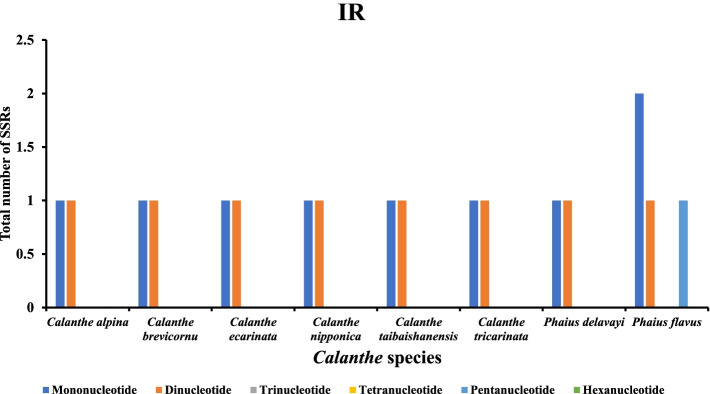
The total number of SSRs identified in the IRs regions of 8 *Calanthe* group species

A total of 507 SSRs were recorded in this study, and the chloroplast genomes of the eight species contained nearly similar numbers of SSRs (57-76) (Fig. 5). *Calanthe alpina* had the highest number of simple repeats (76), while *Phaius delavayi* had the least SSRs (57). Additionally, a base preference was recorded in the base composition of the repeating motifs from mononucleotide SSRs to trinucleotide SSRs, mainly A-T rich repeating motifs. Mononucleotide repeats were the most abundant SSRs (28-45), while hexanucleotide repeats were the least (1-2) in the 8 cp genomes (Table S4). A/T repeats were the most abundant mononucleotide repeats (287), followed by dinucleotide repeat sequences (92) which predominantly consisted of AT/AT repeats and all trinucleotide repeats were AAT/ATT. The least abundant repeats were AAG/CTT, AAATAT/ATATTT, AAGTAT/ACTTAT, ACATAT/ATATGT, and AGATAT/ATATCT (Table S4). The LSC of the eight complete genomes had the highest number of SSRs (385). Mononucleotide repeats were the most abundant ranging from 18-33 (LSC), 8-10 (SSC), and (1-2) IR. Hexanucleotides were the least SSRs in all the regions, with none occurring in the IRs of all the species (Figs. 6, 7, 8, and Table S4).

**Fig. 9:**
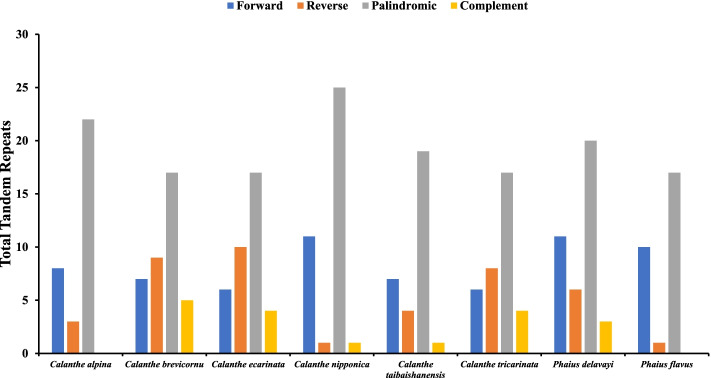
The total number of tandem repeats recorded in the cp genomes of 8 *Calanthe* group species

**Fig. 10:**
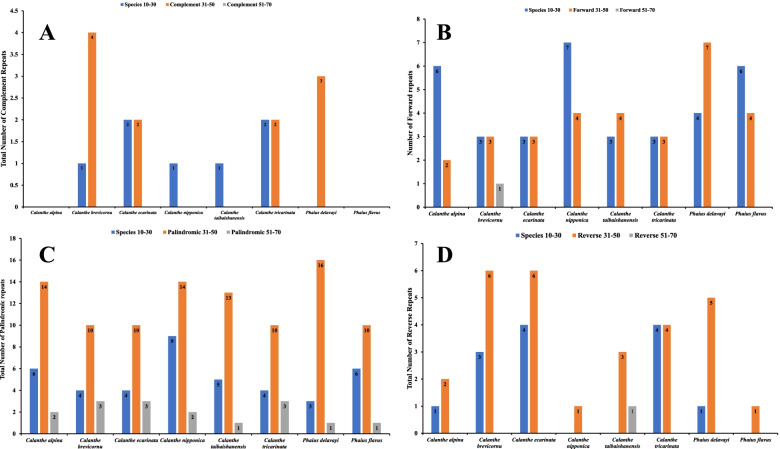
Different types of tandem repeats identified in the cp genomes of 8 *Calanthe* group species. **A** The total number of complement repeats, **B** the total number of forward repeats, **C** the total number of palindromic repeats, and **D** the total number of reverse repeats identified from the cp genomes of 8 *Calanthe* group species

Tandem repeats were detected and are classified as forward (F), palindrome (P), reverse (R), or complement (C), with each repeat having a length of ≥30 bp sequence similarity of ≥90%. A total of 28-40 repeat sequences were identified, and the highest number of repeats were recorded in *P. delavayi *(Fig. 9). Palindromic repeats were the most abundant in all the 8 *Calanthe *group plastomes (17-25), whereas complement repeats were the least abundant (1-5). No complement repeats were found in the chloroplast genomes of *C. alpina* and *P. flavus*. The length of the repeat sequences detected predominantly varied between 31 to 50 bp. Additionally, there were no complement repeat sequences in all the eight cp genomes having 51–70 bp in length (Fig. 10 and Table S5). Overall, the SSRs and tandem repeats in the 8 *Calanthe* group cp genomes showed no significant differences (Kruskal-Wallis,* P *< 0.05; Table S6). The numbers, types and sizes of SSRs and tandem repeats, however, varied greatly across different structural and functional regions of the cp genomes whereby these repeats were abundant in non-coding regions than in the coding regions (Table S3).

### Codon Usage

**Fig. 11:**
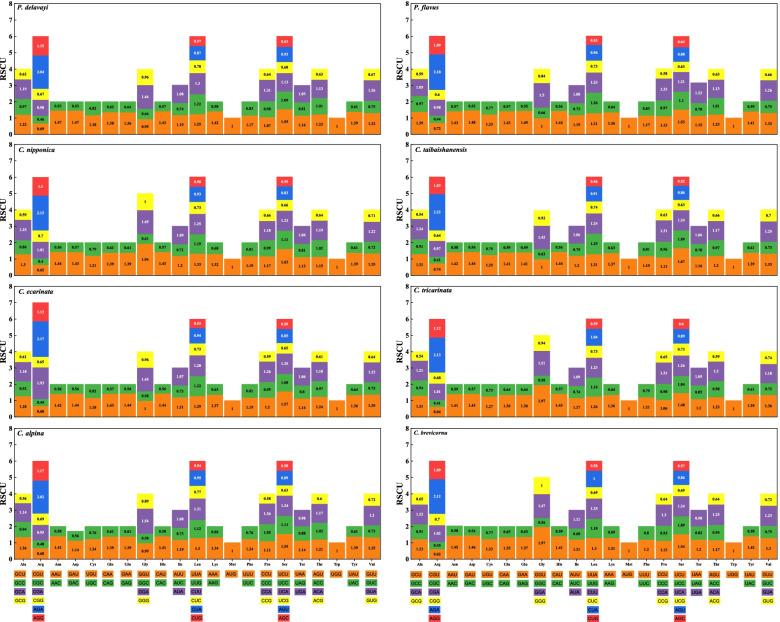
The codon usage distribution in all the protein-coding genes of the complete chloroplast genome of the 8 *Calanthe* group species

The RSCU of the chloroplast genomes of the 8 *Calanthe* group species was calculated using all protein-coding genes, and a total of 50,035-52,904 codons were recorded. The RSCU values for each species displayed an identical codon preference in the 64 codons of the protein-coding genes. In this regard, 30, 31, 32, and 33 codons from *Calanthe taibaishanensis*; *C. alpina*, *C. brevicornu*, *Phaius delavayi*, *Phaius flavus*, and *Calanthe ecarinata*; *C. tricarinata*; and *C. nipponica* respectively exhibited greater preference (RSCU > 1). Two of them, tryptophan (Trp) and methionine (Met), displayed no preferences (RSCU = 1) in all the species. The rest of the codons were least preferred (RSCU < 1). There were no rare codons (RSCU < 0.1) recorded in the CDS genes of the 8 cp genomes of the *Calanthe* group (Fig. 11).

Leucine (Leu), encoded by UUA, UUG, CUU, CUC, CUA, and CUG was the most abundant amino acid, with a proportion of 9.70-10.56 %, which consist 4,993-5,374 of the total number of codons. Serine (Ser), encoded by UCU, UCC, UCA, UCG, AGU, and AGC was the second most plentiful amino with a proportion of 8.93-9.81 % (4,661-5,187codons), whereas tryptophan (Trp), encoded by UGG was the least abundant amino acid encoded, with a proportion of 1.22-1.47 % (641-759 codons) (Table S7). Statistical analysis of the RSCU values in the 8 *Calanthe* group cp genomes did not vary significantly (Kruska-Wallis, *P* < 0.05; Table S8).

### Phylogenetic analysis

**Fig. 12:**
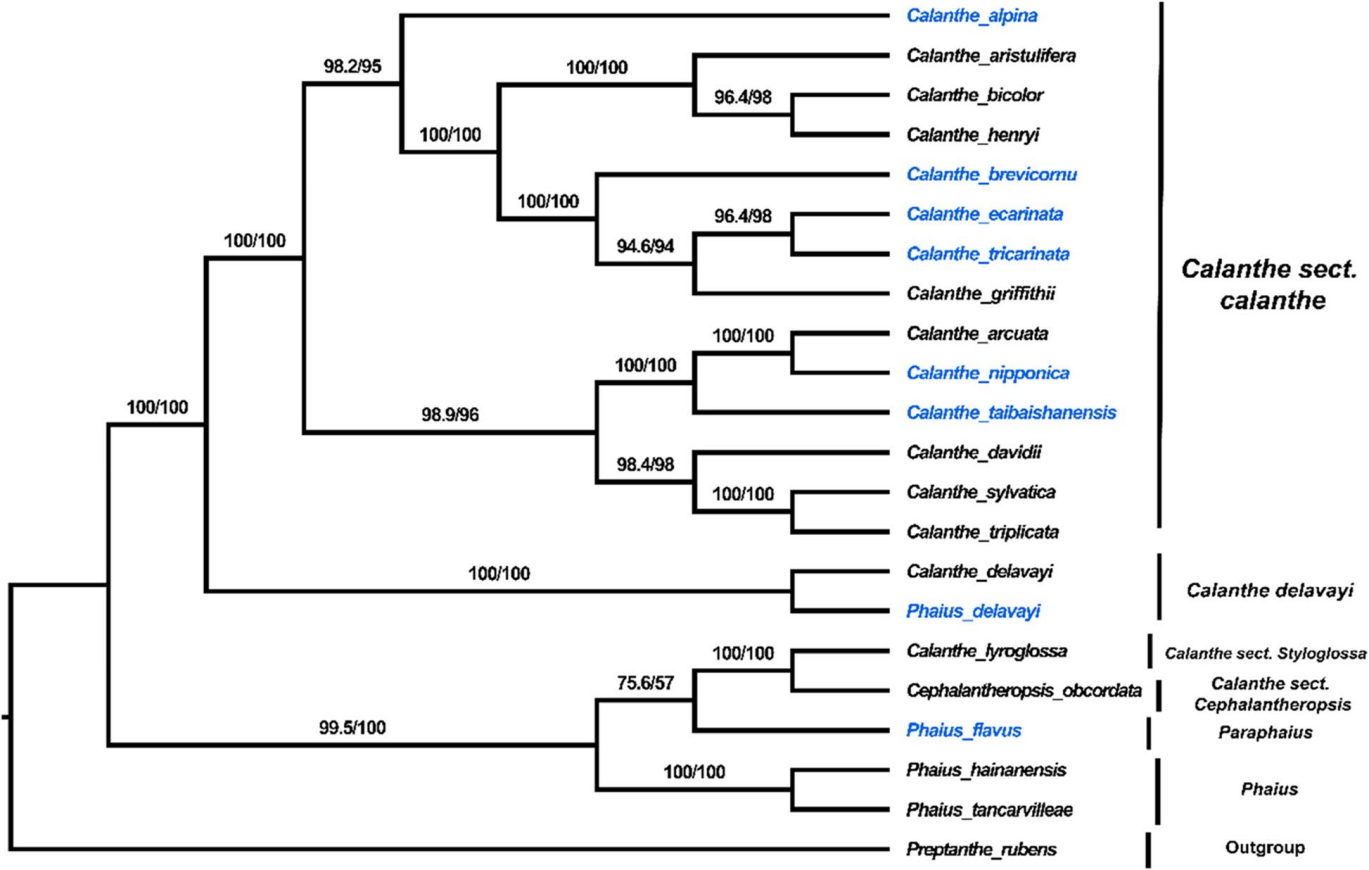
Maximum likelihood tree of the *Calanthe* group reconstructed based on 73 protein-coding genes. The bootstrap proportion values are indicated on the respective nodes. The different sections of the *Calanthe* group are also indicated. The species names colored blue represent our sequenced species plastomes and the species in black represent the species plastome sequences downloaded from the NCBI

The application of high-throughput sequencing technology has enhanced the availability of whole plastid genomes, leading to the resolution of closely related taxa using plastome sequences [24]. The phylogenetic positions of the eight newly sequenced *Calanthe* and *Phaius* species were inferred using a matrix of 64,593 characters (nucleotides). These characters represent the 73 protein-coding genes shared among the eight species in the *Calanthe* group, combined with 14 species of the *Calanthe* alliance, for which their complete chloroplast genome sequences had been officially published in the NCBI database. The ML and BI trees exhibited similar phylogenetic topologies with high bootstrap values and posterior probabilities (Fig. 12; Figure S1). *Phaius* species (excluding *P. delavayi*) form two clades in the phylogenetic tree (Fig. 12; Figure S1). The first clade includes *P. tankervilleae*, and *P. hainanensis* [BP_(ML)_ = 100%, PP = 1.00]. The second clade consists of only *P. flavus* [BP_(ML)_ =75.6/57%, PP =0.9851], closely related to *Cephalantheropsis,* and *Styloglossum*. The third clade was composed of two sister groups: *Cephalantheropsis* (*C. obcordata*) and *Styloglossum* (*C. lyroglossa*) [BP_(ML)_ = 100%, PP = 1.00]. The fourth clade consisted of two species, *P. delavayi*/*C. delavayi* [BP_(ML)_ = 100%, PP = 1.00], while the last clade was made of the rest of the species of section *Calanthe*. All the species of *sect. Calanthe* clustered together in a super clade [BP_(ML)_ = 100%, PP = 1.00], consisting of three lineages and includes 14 *Calanthe* group species. The first lineage includes only *C. alpina* [BP_(ML)_ = 98.2/95%, PP = 1.00], and this taxon is sister to a clade containing the remaining species of this section. The rest of the species formed the other two lineages of this section which include (*C. triplicata*, *C. sylvatica*, *C. davidii*) and (*C. taibaishanensis*, *C. nipponica*, *C. arcuata*) [BP_(ML)_ = 98.9/96%, PP = 1.00] as well as (*C. grifithii*, *C. tricarinata*, *C. ecarinata*, *C. brevicornu*) and (*C. henryi*, *C. bicolor* and *C. aristulifera*) [BP_(ML)_ = 100%, PP = 1.00].

## Discussion

### Comparison of the chloroplast genomes of the 8 *Calanthe* group species

Complete chloroplast sequencing and genomic analyses have revealed that orchid plastomes are highly conserved in terms of size, structure, gene order and content [28–31]. These findings are congruent with results from our study on the 8 plastomes of the *Calanthe* group which revealed that the cp genome of the 8 *Calanthe* group species is a quadripartite structure that varied in size among the species ranging between 150,105 bp (*P. delavayi*) and 158,714 bp (*C. nipponica*). The plastome is divided into four regions consisting of an LSC (83,364bp-87,450bp), IRs (25,222bp-26,430bp), and an SSC (16,297-18,586bp). The inferred structure and contents are consistent with previous research on orchids [32, 33]. The chloroplast genome in angiosperms has a conserved genome structure [34], including two inverted repeats (IRs) which separate a large single-copy section (LSC) and a small single-copy section (SSC). Furthermore, when compared to nuclear and certain plant mitochondrial genomes, chloroplast genomes are smaller and less prone to recombination, providing unique data for studying genome size variation and evolutionary status [35, 36]. These characteristics are useful for comparative studies because they allow researchers to investigate genome divergences across a wide range of evolutionary time, from early land plants [37] to recently domesticated plants, and to detect selection signals of genome size evolution [38].

The genome sizes of the 8 *Calanthe* group species varied in size among the species between 150,105 bp (*P. delavayi*) and 158,714 bp (*C. nipponica*). Previous studies on seed plants have proposed three important factors that cause variation in chloroplast genome size: (1) intergenic region variations, which mainly affects variation in chloroplast genome size within a genus [39, 40]; (2) variation of an IR region [41, 42]; and (3) gene loss, which is an important reason for the shrinking of chloroplast genome size in some plants [41, 42]. The length corresponds to the size range of the cp genomes of most angiosperms [43]. However, the variation in size among cp genomes in orchids been linked to the contraction and expansion of both the inverted repeat regions [4, 28, 44]**.**

Angiosperm plastomes have comparatively little variation in gene content, despite their differences in size range [45], similar to findings from our study which displayed sequence similarity in gene order and arrangement across the 8 *Calanthe* group plastomes. The plastomes’ characteristics and sequence variabilities have been linked species phylogenetic relationships and evolution; thus, closely related species are more likely to have similar plastome sizes and characteristics [46]. A previous study on the evolution of flowering plants plastome architecture revealed that the cumulative influence of transposable elements proliferation greatly dwarfs the impacts of tandem or dispersed gene duplication in increasing genomic DNA content, and the process of long-term genomic fractionation, which is associated with the loss of most gene duplications after whole genome duplication [47]. Transposable elements have been implicated as important factors in gene regulation and adaptation, particularly because gene content is fairly consistent across plants and transposable elements accumulate and degrade rapidly [48–50]. Although this pattern is now known, the underlying causes of constancy of genic content in related orchid genera despite the rapid diversification rate in the family Orchidaceae are far less well understood.

Additionally, the GC contents of the LSC and SSC regions in the 8 *Calanthe* group species were lower compared to that of the IR regions. This occurrence was possibly due to the four rRNA genes, *rrn16*, *rrn23*, *rrn4.5*, and *rrn5* sequences in the IR regions [33].

A few differences were recorded in the protein-coding genes of the complete cp genome of the 8 *Calanthe* group species, despite land plants being generally considered highly conserved [51]. We revealed that protein-coding genes in the *ndh* family differed between *Calanthe* group species. The genes *ndhC*, *ndhF*, and *ndhK* were lost in *P. delavayi* but they were retained in the other species. The loss of these three NADH dehydrogenase subunits is common in orchids and was first reported in this species by Chen in 2020 [4]. In higher plants, the cp genomes contain 11 *ndh* genes (*ndhA-ndhK*) that encode nicotinamide-adenine dinucleotide (NADH) dehydrogenase subunits that associate with nuclear-encoded subunits to form the NADH dehydrogenase-like (NDH) complex, which is involved in cyclic electron flow around photosystem I (PSI) and chlororespiration [52, 53]. Although the chloroplast NDH complex mediates cyclic electron transport in PSI, no negative effects in *ndh*-deficient mutants or transgenics have been observed under suitable growing conditions [29], suggesting that chloroplast *ndh* genes may be unnecessary in autotrophic plants. Evidently, loss or pseudogenization of plastid *ndh* genes has been observed in a variety of photoautotrophic seed plant lineages [54, 55] including *Cymbidium*, *Dendrobium*, *Phalaenopsis*, and *Ophrys* [29, 56–58]. These studies also showed that different orchid species exhibited a variable loss or retention of *the* genes; for instance, *Cymbidium* encodes the *ndhE*, *ndhJ*,and *ndhC* genes [59] while *Oncidium* only encodes the *ndhB* gene [31]. The loss of the *ndh* genes has been linked to evolutionary processes whereby several studies inferred that orchids' ancestral protein-coding *ndh* genes might have been transferred to the nucleus [28, 60]. Fungal symbionts have also been attributed to the lack of functional *ndh* genes; thus, homologous genes from these resources have been presumed to perform the functions of the lost chloroplast-encoded *ndh* genes in some orchids [32, 60]. Nevertheless, this hypothesis is yet to be tested, and the mechanisms underlying the variable loss or retention of *ndh* genes in orchid species are worthy of further research.

### Comparative analysis

DNA barcode technology has been widely used in identifying species, resource management, and phylogenetic and evolutionary studies [61, 62]. The comparative analysis of the 8 *Calanthe* group chloroplast genomes using mVISTA revealed the DNA sequence similarities among related species. No definitive rearrangements or gene inversions were recorded, indicating that the *Calanthe* group plastome was highly conserved [28]. The size of the genome and organization of the intergenic spacers correspond to previously observed variations in the size of the *Calanthe* chloroplast genomes [4].

In line with findings from other studies [63] and those from mVISTA, the LSC and SSC regions were more variable than the IR regions. The mVISTA results revealed the following highly variable regions across the 8 plastomes: *psbA*-*trnK-UUU*, *rps16*-*trnQ-UUG*, *matK-trnK-UUU-rps16*, *trnS-GCU-trnG*-*GCC*, *rpoB-trnC*-*GCA*, *petN-psbM*, *psbM-trnD*-*GUC*, *trnE*-*UUC-trnT*-*GGU*, *trnT*-*GGU*-*psbD*, *ndhK-trnM*-*CAU*, *atpB-rbcL*, *rbcL-accD*, *accD-psaI*, *petA-psbJ*, *psbE-petL*, *trnV*-*GAC-rps12*, *ccsA-ndhD*, *trnL*-*UAA*, *trnL*-*GAU*, *ndhF*, *ndhI*, *rps15*, *trnP-UGG*, *rpl33*, *clpP*, *psbT*, *rpl16*, *rpl14*, *rps8* and *rpl32.* Interestingly, these highly variable regions were mostly similar to the mutational hotspots identified in other species of the *Calanthe* alliance [4], suggesting that these variable loci can be used as important references for future studies on the evolution and diversity in the *Calanthe* alliance. The nucleotide diversity was higher in the LSC and SSC compared to SSC regions and identified the following hypervariable regions across the *Calanthe *group plastome:* trnS-GCU****-****trnG-GCC*, *rpoB****-****trnC-GCA*, *trnE-UCC****-****trnT-GGU*, *rpl32****-****trnL-UAG*, *ccsA-ndhD* and protein-coding genes *psbL*, *clpP* and *rpl32*. The markedly high divergence observed in these genes and intergenic regions is similar to that observed in other angiosperms [4, 64, 65] and may be attributed to rapid genome evolution due to higher mutation rates compared to other regions [66].

The IR regions are relatively conserved compared to the SSC and LSC regions in the *Calanthe* group plastomes. Significant variation was only observed in the LSC/IRb junction, which displayed three occurrences in the eight species. At the same time, the remaining three (IRb/SSC, SSC/IRa, and IRa/LSC) are conservative and stable. Contraction in the IR was detected due to the loss of the *ndhF* gene in *P. delavayi*. Previous studies have highlighted that the loss of *ndh* genes significantly contributes to the instability of the IR/SSC borders in orchids [58, 67]. The variation in size and evolutionary events in different plants may also be linked to the expansion and contraction of the junctions in the different regions of the chloroplast plastomes [23, 68, 69]. The location of the boundary, particularly the expansion and contraction, has been successfully used to infer phylogenetic relationships and provide insights on the evolution of the lineages in Apiaceae [70], ferns [71], Poaceae [72], Pinaceae [28], and many monocots [73]. Nevertheless, even though overall genomic structures and gene orders are highly conserved, orchid plastomes exhibited clear differences at the IR/SSC boundaries, which cannot readily be used in a phylogenetic study. Furthermore, the *ndh* genes in SSC regions have been lost independently across orchid genera [58, 67], corroborating the findings by Kim et al. (2015) [30] which proposed that the instability of orchid IR/SSC junctions was highly related to the loss of the *ndhF* gene. Even so, the mechanism underlying the variations in the sequences flanking the IR/SSC junctions of orchid plastomes remains unknown. Therefore, our findings from the present study on the IR boundary does not provide the necessary information to elucidate the evolutionary relationships within the *Calanthe* group, thus, additional sampling of *Calanthe* spp. and related genera will allow for clear and specific tests [74].

### Molecular Markers

Simple sequence repeats have distinct features that make them efficient genetic markers such as abundance in number, highly repetitive, a simple structure, maternal inheritance of chloroplast genomes, and relatively conserved [75]. SSRs and repeat sequences have been extensively used in identifying species, phylogenetic analysis, population evolution studies, and system geography of various species [76]. In this regard, the variation in the number and distribution of SSRs and tandem repeats in all the 8 *Calanthe* group genomes and different regions of the whole plastomes were detected. Repeats were widespread in the non-coding regions compared to the coding regions, consistent with previous reports on other species [30, 77]. The chloroplast genome rearrangement and nucleotide substitution can be attributed to the differential distribution of these repeats [78].

Additionally, the SSRs were mainly distributed in LSC, compared to the SSC and IR regions illustrating that the distribution of SSRs was dependent on their locations in the chloroplast genome [79]. These repeats can therefore be used to develop genetic markers for phylogenetic studies. The identified SSR and tandem repeats can also be used to investigate the genetic structure, diversity, phylogeny, and differentiation of species in the *Calanthe* alliance and other orchid species.

### Relative Synonymous Codon Usage

The RSCU value is the ratio of the usage frequency of a specific codon to the expected frequency and can eradicate the influence of amino acid composition on the codon usage [80]. Additionally, RSCU promotes the detection of synonymous codons [81]. Most codons with RSCU values greater than 1 ended with A or U, whereas those ending with a C or G had RSCU values of less than 1. These findings are consistent with previous studies [82, 83].

Compositional constraints and translational selection are presumed as the main factors that result in the codon usage variation among protein-coding genes in and across the plastomes [84]. Moreover, compositional bias has been shown to determine the codon usage variation amidst genes in most AT or GC-rich organisms [85]. Analysis of RSCU may provide a basis for studying specific mechanisms causing biased preference of synonymous codons in different species [86]. In addition, it plays a crucial role in both practical and theoretical studies on the basics of molecular biology [87].

### Phylogenetic and taxonomic implications

Phylogenetic analyses using chloroplast genome data have been used successfully to infer the evolutionary relationships among angiosperms [30, 81, 83]. Phylogenetic studies of Orchidaceae using complete plastomes are in a rather early stage due to paucity of plastome sequences. However, the relationships among major orchid lineages determined using whole plastomes (species tree) agree well to the large-scale phylogenetic studies of Orchidaceae using two or three genes (gene tree). Therefore, by sequencing more Orchidaceae complete plastomes can help resolve the pressing phylogenetic problem. Molecular datasets comprising of protein-coding genes, non-coding regions, and hypervariable regions have been used to infer major phylogenetic relationships between major orchids clades [88]. However, there are numerous uncertainties about the phylogenetic placement of several subtribes and genera. This knowledge gap is caused by a lack of both taxonomic and genomic sampling efforts required to cover all major orchid clades (subtribes/groups of genera) [89]. In this study the relationships among the *Calanthe* alliance genera included in our phylogenetic assessment are generally consistent with recent studies [6, 15], although there are a few differences.

Previous studies on the *Calanthe* group based on morphological characteristics (adnation of the lip to the column) recognized *Calanthe* and *Phaius* as paraphyletic [21, 90, 91]. In addition, *P. delavayi*, which was previously included in genus *Phaius* based on its floral morphology by Pridgeon [14], was later classified as a member of genus *Calanthe* based on molecular evidence (ITS and cpDNA) by Zhai [15]. These findings are in agreement with results from our study as further discussed in the subsequent section.

In the present study, *Phaius* species (excluding *P. delavayi*) form two clades. The first clade includes *Phaius*: *P. tankervilleae* and *P. hainanensis,* while the second divergent clade comprised only one species of *Phaius*: *P. flavus.* These results are consistent with those of Zhai [15], who were the first to report the divergence within *Phaius*, excluding *C. delavayi*/ *P. delavayi* based on ITS and cpDNA data*.* Therefore, we strongly support the proposal by Zhai’s study that *Phaius* is restricted to the lineage that includes species such as *P. tankervilleae* and *P. hainanensis*. The clade consisting of *P. tankervilleae* and *P. hainanensis* is characterized by caducous floral bracts and eight pollinia in two groups separated from each other. In contrast, the second clade, which includes species such as *P. flavus*, has distinct features, including persistent floral bracts, pollinia which occur in two categories attached to a sticky substance by caudicles [21, 92]. Zhai’s study suggested the inclusion of a new genus, *Paraphaius*, to encompass the lineage consisting of *P. flavus*. Results from our study on the phylogenetic position of *P. flavus* [BP_(ML)_ =75.6/57%, PP =0.9851] are consistent with Zhai's in 2014. This species was clustered together with the clade supporting *Calanthe lyroglossa *of *Calanthe sect. Styloglossa* and *Cephalantheropsis obcordata *of* Calanthe sect. Cephalantheropsis*, supporting Zhai’s proposal of recognizing subsection *Paraphaius* to encompass *P. flavus*. However, more sampling is required to help further resolve the phylogeny of this species adequately.

**Fig. 13:**
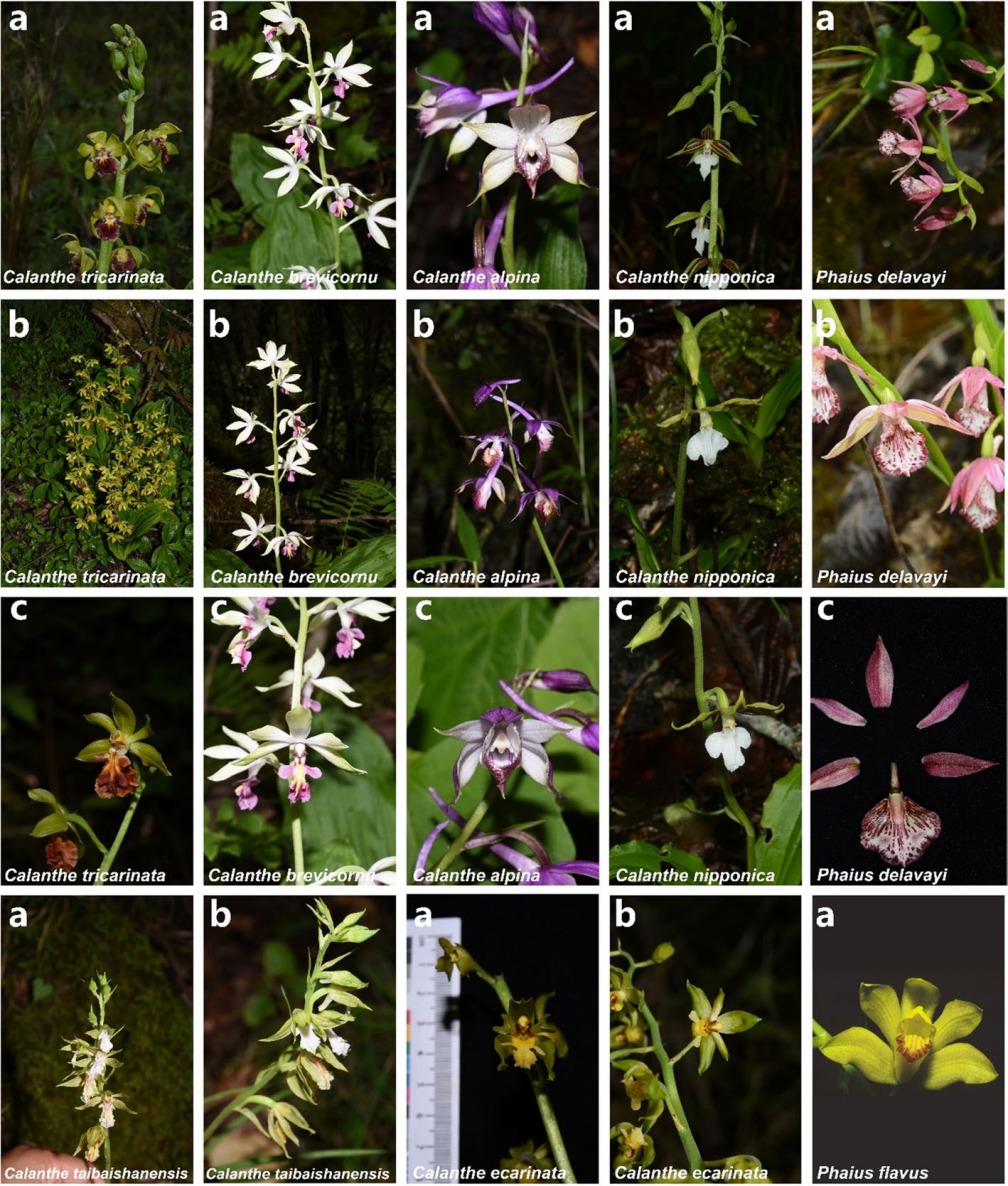
Morphological characteristics of the *Calanthe* group species. Photos taken by Guangwan Hu. The picture of *P. flavus* was taken by Lourens Grobler (http://www.orchidspecies.com/phaiusflavus.html)

Presently, *Phaius delavayi* has become a vital species in the taxonomic studies of the *Calanthe* alliance due to its complex taxonomic history between *Calanthe* and *Phaius* [1, 17]. Previous studies have identified *Phaius delavayi* as a link between *Calanthe* and *Phaius* [16]*.* It is Morphologically identical to *Calanthe* due to its relatively small individual, basal leaves, elongated column, and inconspicuous pseudobulbs [22]. Nevertheless, it has similar morphological features characterized in *Phaius* because of its long labellum embracing its column (Fig. 13) [15]. Previously, based on morphological characteristics, Chen in 1999 [21], treated this species as a member of the *sect. Calanthe*, although in their work, the Flora of China project in 2009 [91], they accepted a taxonomic placement of this species within *Phaius* as earlier proposed by Perner and Cribb [90]. However, Zhai’s study on the phylogenetic relationships in the *Calanthe* alliance in China suggested that *P. delavayi* should be retained within *Calanthe* rather than within *Phaius*. In addition, this study proposed the inclusion of a new section, *Alpinocalanthe*, to accommodate this unique taxon due to its phylogenetic placement and distinct morphological characteristics, namely: small plants, persistent bracts in flowers; labellum adnate to column wings at the basal area, a slender column; a somewhat 3-lobed labellum, circumjacent column and a disk-shaped labellum having three shortly hairy ridges. Based on the ML and BI trees using coding sequences, our results indicate *P. delavayi* [BP_(ML)_ = 100%, PP = 1.00], is closely related to *Calanthe* than to *Phaius*, consistent with findings by Zhai [15]. In this regard, we also support the use of the name *Calanthe delavayi* instead of *Phaius delavayi*.

*Calanthe sect. Calanthe* is the largest infrageneric group of the genus *Calanthe*, including approximately 140 species worldwide, out of which 50 species occur in China [15, 93]. In the current study, an independent super clade encompassing all the 14 *Calanthe* group species of this section was identified. The results revealed that the primary relationship was consistent with other studies among the *Calanthe section Calanthe* group [4, 5, 94]. The results obtained from the Maximum Likelihood (ML)/Bayesian Inference (BI) analyses revealed that the genus *Calanthe* forms a high support clade as a paraphyletic group [BP_(ML)_ = 100%, PP = 1.00].

Our conclusion, however, on the paraphyly of the *Calanthe* group differs from that of the latest study by Chen et al. (2020) on the plastome structure and adaptive evolution of *Calanthe* s.l. which revealed the monophyly of *Calanthe* s.l. species [4]. This rigorous but taxonomically under-sampled study completely distinguished the seven species of the *Calanthe* group in their phylogenetic inference with high bootstrap support values. The discrepancies noted between this study and our present study is most likely due to large variation in the number of characters and taxa included [95]. Regardless of the fact that our matrix is character-rich and thus less prone to error caused by individual genes [96], we must highlight that our taxon sampling is highly fragmentary, and supplementary plastome sequences from poorly sampled and/or unsampled genera in the *Calanthe* alliance may result in topological changes.

Based on protein coding genes shared among all the target *Calanthe* group species, our study on the *calanthe* group chloroplast genome provides valuable genetic information on the eight newly sequenced species, highlights the power of using plastome data to resolve phylogenetic relationships between closely related species, and will facilitate future phylogenetic studies on orchids.

## Conclusions

In conclusion, the complete plastomes can provide relevant information for resolving evolutionary disputes between closely related taxa. In this study, the complete chloroplast genome of 8 *Calanthe* group species were sequenced and compared. In addition, phylogenetic relationships in the *Calanthe* group were resolved with high or moderate support values. The highly divergent genes and regions of cp genomes identified in this study can be used as effective DNA barcodes in genetic diversity studies and in phylogenetic analyses. Further chloroplast genome sequencing of orchids is necessary to clarify the diversity of complete plastomes and to facilitate species identification, phylogenic analysis, and elucidate evolutionary relationships within orchid species.

## Materials and methods

### Sample collection

Collection permits for sample collection were granted by the Sichuan Forestry and Grassland Administration Sichuan province, China and Yunnan Forestry and Grassland Administration, Yunnan province, China. Fresh leaves from 8 *Calanthe* group species were collected from the Sichuan and Yunnan provinces of China (Table S1). Guang-Wan Hu, Jiaxin Yang and Xiang Dong performed formal identification of the samples after collection whereby the leaf samples of *Calanthe tricarinata*, *Calanthe alpina*, *Calanthe nipponica*, *Calanthe taibaishanensis*, *Calanthe ecarinata*, *Calanthe brevicornu*, *Phaius delavayi*, and *Phaius flavus* and stored in seal-bags containing silica gel before DNA extraction. The sample specimen of each species was then deposited at the Herbarium of Wuhan Botanical Garden (HIB) with specific voucher numbers (Table S1).

### Chloroplast Genome Sequencing and Assembly

The genomic DNA was extracted from about 100 micrograms of the leaves using a modified cetyltrimethylammonium bromide (CTAB) method [97]. Genome sequencing was performed using the Illumina platform at Novo gene Company (Beijing, China), followed by filtration of low-quality data and adaptors and assembly of the clean data was obtained using GetOrganelle-1.6.2 software [98], using *Calanthe triplicata* (NC_024544) as the reference genome. Bandage software was then used to check the final results of the assembled genome after manual corrections. The optimal result was selected, after which manual adjustment of these results was also made. Lastly, inverted repeat regions were identified using Geneious Prime 2019.2.1 (https://www.geneious.com).

### Genome annotation

The annotation of the assembled genomes was performed using GeSeq online tool with default settings [99], followed by confirmation of tRNAs annotations using the tRNAscan-SE [100]. The Plastid Genome Annotator (PGA), a standalone command-line annotation tool, validated the *Calanthe* cp genomes [101]. The gene map of the complete cp genomes was drawn using OrganellarGenome DRAW software [102] (Fig. 1). The annotated complete chloroplast genomes were submitted to the GenBank database with accession numbers as follows: *Calanthe alpina* (OL322023), *C. brevicornu* (OL348396), *C. ecarinata* (OL348397), *C. nipponica* (OL348398), *C. taibaishanensis* (OL351366), *C. tricarinata* (OL351367), *Phaius delavayi* (OL351368), and *P. flavus* (OL351369).

### Genome comparison and sequence divergence

The IRscope [103] was used in the comparison of the border junctions of inverted repeat (IR), small single copy (SSC), and large single copy (LSC) regions. Using Shuffle-LAGAN mode, the mVISTA software [104] was used to compare and visualize the complete chloroplast genomes of the eight species with *C. nipponica* as the reference. Additionally, all the 8 *Calanthe* alliance cp genome sequences were aligned using MAFFT v7.409 [105]. Further, we performed a sliding window analysis to evaluate the variability (*Pi*) over the plastomes using DnaSP v5.10 [106] at 600 base pairs window length and 200 base-pair step size.

### Repeat structure and Simple Sequence Repeats (SSRs) analysis

The visualization of forward, palindrome, reverse, and complement repeats in the *Calanthe* group genome was conducted using REPuter [107], with the minimum repeat size being set at 30 bp, maximum at 50 bp and sequence identity of no less than 90% (hamming distance= 3). Identification of simple sequence repeats (SSRs) was performed using MISA (https://webblast.ipk-gatersleben.de/misa/index.php) [108], with the minimum number of repeats as follows: 10 for mono-, 5 for di-, 4 for tri-, and 3 for tetra-, 3 for penta-, and 3 for hexanucleotide SSRs.

### Relative synonymous codon usage

All the protein-coding genes for the combined genomes were extracted using MEGA 7 [109] software which was then used to calculate the relative synonymous codon usage (RSCU) ratio. RSCU values >1 represent frequently used codons than expected, while values <1 signify the opposite. Codons having no preference value are set to 1.00.

### Phylogenetic analysis

Phylogenetic relationship analysis was conducted using 73 PCGs extracted from the complete cp genome sequences of the 8 *Calanthe* group taxa mentioned above, with one outgroup, *Preptanthe rubens* (NC_050869) and thirteen previously sequenced members of the *Calanthe* alliance downloaded from the NCBI database (Table S2). Multiple sequence alignment of the 22 complete cp genome sequences was performed using MAFFT with default parameters. The best fit model was identified using the Model Finder program [110] integrated into Phylosuite. The best-fit models for the phylogenetic analysis were GTR GTR+G, GTR+I+G, and setting (rcluster) for the concatenated alignment as implemented in ModelFinder. Phylogenetic reconstructions were performed using the maximum likelihood (ML) method using the IQ-Tree integrated in Phylosuite [111]: a GUI-based software written in python 3.6.7. The analyses were run with 1000 bootstrap replicates. Phylogenies were then inferred by Bayesian Inference using MrBayes 3.2.6 [112] under the GTR+G+F model (2 parallel runs, 10,000,000 generations and sampled at a frequency of 1000 generations), in which the first 25% of the sampled trees were discarded as burn-in. The remaining trees were used to build a majority rule consensus tree and establish posterior probability values for each branch. Finally, the trees were refined and visualized using FigTree v1.4.4 and later combined using AI software.

## Supplementary Information

**Additional file 1.**

**Additional file 2.**

**Additional file 3.**

**Additional file 4.**

**Additional file 5.**

**Additional file 6.**

**Additional file 7.**

**Additional file 8.**

**Additional file 9.**

## Data Availability

The complete chloroplast genomes generated in this study were submitted to the NCBI database (https://www.ncbi.nlm.nih.gov/) with GenBank accession numbers as follows; *Calanthe alpina* (OL322023), *C. brevicornu* (OL348396), *C. ecarinata* (OL348397), *C. nipponica* (OL348398), *C. taibaishanensis* (OL351366), *C. tricarinata* (OL351367), *Phaius delavayi* (OL351368), and *P. flavus* (OL351369). All other data and material analyzed in the current study are included in the manuscript and the supplementary information files.

## Abbreviations

AT: Adenine-Thymine
Bp: Base Pair
CTAB: Cetyl Trimethyl Ammonium Bromide
cp: Chloroplast
cpDNA: Chloroplast DNA
DNA: Deoxyribonucleic Acid
GUI-based software: Graphical User Interface based software
GC: Guanine-Cytosine
IRs: Inverted Repeats
LSC: Large Single Copy
MAFFT: Multiple Alignment using Fast Fourier Transform
MEGA: Molecular Evolutionary Genetic Analysis
MISA: Microsatellite Identification Tool
ML: Maximum Likelihood
NGS: Next Generation Sequencing
NCBI: National Centre for Biotechnology Information
PCG: Protein Coding Gene
RSCU: Relative Synonymous Codon Usage
rRNA: ribosomal RNA
RNA: Ribonucleic Acid
SSC: Small Single Copy
SSR: Simple Sequence Repeat
tRNA: transfer RNA
