# Supplementary material for: Plastome structure of 8 Calanthe s.l. species (Orchidaceae): comparative genomics, phylogenetic analysis
Source: BMC Plant Biol. 2022 Aug 3;22:387. doi: 10.1186/s12870-022-03736-0 (PMC9347164; doi:10.1186/s12870-022-03736-0)
Supplement: Supplementary file 1 — Additional file 1. [file 12870_2022_3736_MOESM1_ESM.docx]

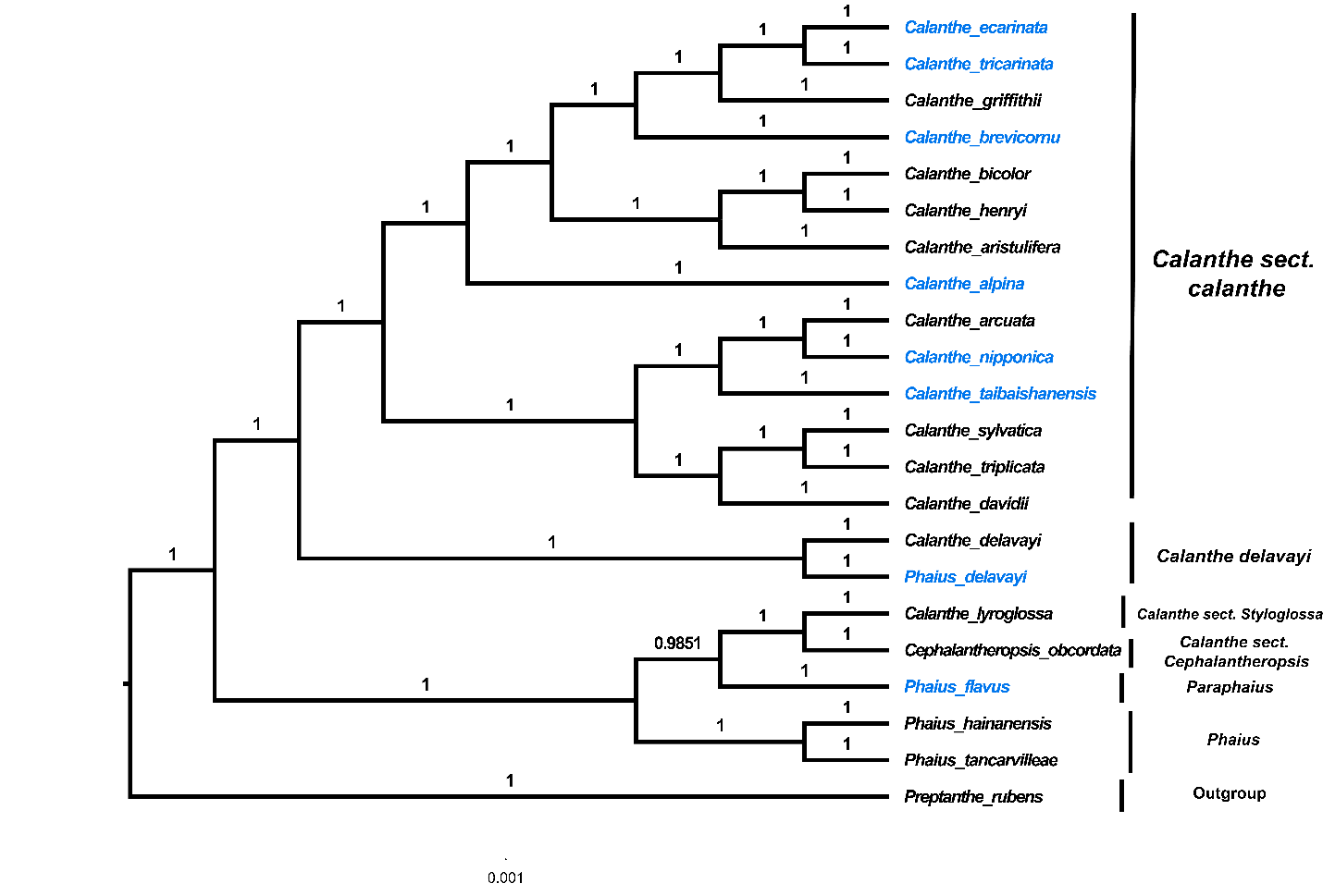


**Figure S1.** BI tree based on the sequences of 73 shared protein-coding genes of the *Calanthe* group plastomes. Posterior probability values are indicated on the respective nodes. The different sections of the *Calanthe* group are also indicated.
