# Supplementary material for: Plastome structure of 8 Calanthe s.l. species (Orchidaceae): comparative genomics, phylogenetic analysis
Source: BMC Plant Biol. 2022 Aug 3;22:387. doi: 10.1186/s12870-022-03736-0 (PMC9347164; doi:10.1186/s12870-022-03736-0)
Supplement: Supplementary file 3 — Additional file 3. [file 12870_2022_3736_MOESM3_ESM.docx]

Table S2:*Calanthe* species used in phylogenetic analysis including the newly sequenced 8 *Calanthe* group species in the current study.

| **Species name** | **Accession number** |
| --- | --- |
| *Calanthe arcuata* | MK934523 |
| *Calanthe aristulifera* | NC_046812 |
| *Calanthe bicolor* | NC_046813 |
| *Calanthe davidii* | NC_037438 |
| *Calanthe delavayi* | NC_046576 |
| *Calanthe griffithii* | MZ474966 |
| *Calanthe henryi* | NC_054177 |
| *Calanthe lyroglossa* | NC_050867 |
| *Calanthe sylvatica* | NC_044633 |
| *Calanthe triplicata* | NC_024544 |
| *Cephalantheropsis obcordata* | MN708351 |
| *Phaius hainanensis* | NC_057607 |
| *Phaius tancarvilleae* | MN708349 |
| *Calanthe alpina* | OL322023 |
| *Calanthe brevicornu* | OL348396 |
| *Calanthe ecarinata* | OL348397 |
| *Calanthe nipponica* | OL348398 |
| *Calanthe taibaishanensis* | OL351366 |
| *Calanthe tricarinata* | OL351367 |
| *Phaius delavayi* | OL351368 |
| *Phaius flavus* | OL351369 |
| *Preptanthe rubens* | NC_050869 |
