# Supplementary material for: Plastome structure of 8 Calanthe s.l. species (Orchidaceae): comparative genomics, phylogenetic analysis
Source: BMC Plant Biol. 2022 Aug 3;22:387. doi: 10.1186/s12870-022-03736-0 (PMC9347164; doi:10.1186/s12870-022-03736-0)
Supplement: Supplementary file 4 — Additional file 4. [file 12870_2022_3736_MOESM4_ESM.docx]

Tandem repeats in *C. alpina*

| **Species** | **Size** | **Location 1** | **Type** | **Size** | **Location 2** | **Region** | **Gene region** | |
| --- | --- | --- | --- | --- | --- | --- | --- | --- |
| *Calanthe alpina* | 59 | 2 | P | 59 | 2 | LSC | | IGS (*rps19_psbA*) |
| *Calanthe alpina* | 65 | 63602 | P | 65 | 63602 | LSC | | *cemA* |
| *Calanthe alpina* | 48 | 29656 | P | 48 | 29656 | LSC | | *petN* |
| *Calanthe alpina* | 46 | 42248 | P | 46 | 42248 | LSC | | *psaA* |
| *Calanthe alpina* | 34 | 118825 | P | 34 | 118825 | SSC | | *ndhA* |
| *Calanthe alpina* | 39 | 3662 | R | 39 | 3662 | LSC | | IGS (*trnK-UUU*) |
| *Calanthe alpina* | 39 | 43728 | F | 39 | 100717 | LSC/IR | | IGS (*trnK-UUU_rps16*, *rps12_trnV-GAC*) |
| *Calanthe alpina* | 39 | 43728 | P | 39 | 141324 | LSC/IR | | IGS (*trnK-UUU_rps16*, *rrn16_rps12*) |
| *Calanthe alpina* | 32 | 27851 | P | 32 | 27851 | LSC | | IGS (*rpoB_trnC-GCA*) |
| *Calanthe alpina* | 32 | 55084 | P | 32 | 55084 | LSC | | *atpB* |
| *Calanthe alpina* | 36 | 28744 | P | 36 | 28744 | LSC | | IGS (*trnC-GCA*) |
| *Calanthe alpina* | 33 | 6866 | P | 33 | 6866 | LSC | | IGS (*rps16_trnQ-UUG*) |
| *Calanthe alpina* | 37 | 116486 | P | 37 | 116486 | SSC | | IGS (*rps15_ycf1*) |
| *Calanthe alpina* | 34 | 63874 | P | 34 | 63880 | LSC | | *petA* |
| *Calanthe alpina* | 30 | 8374 | P | 30 | 45171 | LSC | | IGS (*psbI_trnS-GCU*, *trnS-GGA*) |
| *Calanthe alpina* | 30 | 104030 | P | 30 | 104069 | IR | | IGS *(rrn16_trnL-GAU)* |
| *Calanthe alpina* | 30 | 104030 | F | 30 | 137981 | IR | | IGS (*rrn16_trnL-GAU, trnL-GAU_rrn16*) |
| *Calanthe alpina* | 30 | 104069 | F | 30 | 138020 | IR | | IGS (*rrn16_trnL-GAU*) |
| *Calanthe alpina* | 30 | 137981 | P | 30 | 138020 | IR | | IGS (*trnL-GAU_rrn16*) |
| *Calanthe alpina* | 34 | 83787 | R | 34 | 83792 | LSC | | *rpl16* |
| *Calanthe alpina* | 31 | 124634 | P | 31 | 125023 | SSC | | ndhD, IGS (*ndhD_ccsA*) |
| *Calanthe alpina* | 33 | 12591 | P | 33 | 12591 | LSC | | IGS (*ndhD_ccsA*) |
| *Calanthe alpina* | 30 | 5102 | P | 30 | 5102 | LSC | | *rps16* |
| *Calanthe alpina* | 30 | 5102 | F | 30 | 5106 | LSC | | *rps16* |
| *Calanthe alpina* | 30 | 36651 | P | 30 | 36651 | LSC | | IGS (*trnG-UCC_trnfM-CAU*) |
| *Calanthe alpina* | 32 | 35816 | P | 32 | 45171 | LSC | | IGS (*psbC_trnS-UGA*, *ycf3_trnS-GGA*) |
| *Calanthe alpina* | 32 | 48526 | P | 32 | 48562 | LSC | | IGS (*trnF-GAA_ndhJ*) |
| *Calanthe alpina* | 32 | 54960 | F | 32 | 72683 | LSC | | IGS (*atpB_rbcL, psbB_clpP*) |
| *Calanthe alpina* | 31 | 71844 | P | 31 | 71853 | LSC | | *clpP* |
| *Calanthe alpina* | 30 | 9851 | F | 30 | 36576 | LSC | | *trnG-GCC*, IGS (*psbZ_trnG-UUC*) |
| *Calanthe alpina* | 30 | 31818 | R | 30 | 46724 | LSC | | IGS (*trnE-UUC_trnT-GGU*, *trnt-UGU_trnL-UAA*) |
| *Calanthe alpina* | 30 | 38827 | F | 30 | 41051 | LSC | | *psbB,psA* |
| *Calanthe alpina* | 30 | 43740 | F | 30 | 100729 | LSC/IR | | *ycf3*, IGS (*rps12_trnV-GAC*) |
| *Calanthe alpina* | 30 | 43740 | P | 30 | 141321 | LSC/IR | | *ycf3*, IGS *(trnV-GAC_rps12*) |

Tandem repeats in *C. brevicornu*

| **Species** | **Size** | **Location 1** | **Type** | **Size** | **Location 2** | **Region** | **Gene region** |
| --- | --- | --- | --- | --- | --- | --- | --- |
| *Calanthe brevicornu* | 59 | 23 | P | 59 | 23 | LSC | IGS (*psbA_rps19*) |
| *Calanthe brevicornu* | 52 | 13139 | F | 52 | 13193 | LSC | *atpF* |
| *Calanthe brevicornu* | 48 | 30368 | P | 48 | 30368 | LSC | IGS (*petN_psbM*) |
| *Calanthe brevicornu* | 57 | 65065 | P | 57 | 65065 | LSC | IGS (*petA_psbJ*) |
| *Calanthe brevicornu* | 55 | 49364 | P | 55 | 49364 | LSC | IGS (*trnf-GAA_ndhJ*) |
| *Calanthe brevicornu* | 40 | 116324 | F | 40 | 116343 | SSC | IGS (*ndhF_rpl32*) |
| *Calanthe brevicornu* | 34 | 124983 | P | 34 | 124983 | SSC | *ndhA* |
| *Calanthe brevicornu* | 39 | 3616 | R | 39 | 3616 | LSC | *psbC* |
| *Calanthe brevicornu* | 39 | 44552 | F | 39 | 102297 | LSC, IR | *ycf3* |
| *Calanthe brevicornu* | 39 | 44552 | P | 39 | 143203 | LSC, IR | IGS (*rps12_trnV-GAC*) |
| *Calanthe brevicornu* | 32 | 28431 | P | 32 | 28431 | LSC | IGS (*trnC-GCA_rpoB*) |
| *Calanthe brevicornu* | 36 | 29411 | P | 36 | 29411 | LSC | IGS (*trnC-GCA_petN*) |
| *Calanthe brevicornu* | 37 | 127348 | P | 37 | 127348 | SSC | IGS (*rps15_ycf1*) |
| *Calanthe brevicornu* | 37 | 128718 | P | 37 | 128718 | SSC | *ycf1* |
| *Calanthe brevicornu* | 30 | 8418 | P | 30 | 45993 | LSC | *trnS-GCU*, IGS (*ycf3_trS-GGA*) |
| *Calanthe brevicornu* | 32 | 47416 | R | 32 | 47416 | LSC | IGS (*trnT-UGU_trnL-UAA*) |
| *Calanthe brevicornu* | 31 | 47433 | C | 31 | 62304 | LSC | IGS (*trnT-UGU_trnL-UAA*, *ycf4_cemA*) |
| *Calanthe brevicornu* | 31 | 51288 | R | 31 | 51288 | LSC | IGS (*ndhC_trnV-UAC*) |
| *Calanthe brevicornu* | 31 | 116336 | F | 31 | 116355 | SSC | IGS (*ndhF_rpl32*) |
| *Calanthe brevicornu* | 31 | 118786 | P | 31 | 119177 | SSC | IGS (*ccsA_ndhD*), *ndhD* |
| *Calanthe brevicornu* | 33 | 4629 | R | 33 | 4633 | LSC | IGS (*rps16_trnK-UUU*) |
| *Calanthe brevicornu* | 30 | 37473 | P | 30 | 37473 | LSC | IGS (*trnG-UCC_trnfM-CAU*) |
| *Calanthe brevicornu* | 30 | 102731 | R | 30 | 102731 | IR | IGS (*rps12_trnV-GAC*) |
| *Calanthe brevicornu* | 30 | 102731 | C | 30 | 142778 | IR | IGS (*rps12_trnV-GAC*) |
| *Calanthe brevicornu* | 30 | 128549 | P | 30 | 128549 | SSC | *ycf1* |
| *Calanthe brevicornu* | 30 | 142778 | R | 30 | 142778 | IR | IGS (*trnV-GAC_rps12*) |
| *Calanthe brevicornu* | 32 | 36638 | P | 32 | 45993 | LSC | IGS (*psbC_trnS-UGA*), *trnS-GGA* |
| *Calanthe brevicornu* | 32 | 102714 | R | 32 | 102723 | IR | IGS (*rps12_trnV-GAC*) |
| *Calanthe brevicornu* | 32 | 102714 | C | 32 | 142784 | IR | IGS (*rps12_trnV-GAC*) |
| *Calanthe brevicornu* | 32 | 102723 | C | 32 | 142793 | IR | IGS (*rps12_trnV-GAC*) |
| *Calanthe brevicornu* | 32 | 142784 | R | 32 | 142793 | IR | IGS *(rps12_trnV-GAC*) |
| *Calanthe brevicornu* | 31 | 73679 | P | 31 | 73688 | LSC | *clpP* |
| *Calanthe brevicornu* | 31 | 102707 | C | 31 | 142807 | IR | IGS (*rps12_trnV-GAC*) |
| *Calanthe brevicornu* | 30 | 10341 | F | 30 | 37398 | LSC | *trnG-GCC*, *trnG-UCC* |
| *Calanthe brevicornu* | 30 | 32563 | R | 30 | 47576 | LSC | IGS (*trnE-UuC_trnT-GGU*) |
| *Calanthe brevicornu* | 30 | 39649 | F | 30 | 41873 | LSC | *psaB*, *psaA* |
| *Calanthe brevicornu* | 30 | 44564 | F | 30 | 102309 | LSC, IR | ycf3, IGS (*rps12_trnV-GAC*) |
| *Calanthe brevicornu* | 30 | 44564 | P | 30 | 143200 | LSC, IR | *ycf3*, IGS (*rps12_trnV-GAC*) |

Table S3: Tandem repeats for *C. ecarinata*

| **Species** | **Size** | **Location 1** | **Type** | **Size** | **Location 2** | **Region** | **Gene region** |
| --- | --- | --- | --- | --- | --- | --- | --- |
| *Calanthe ecarinata* | 59 | 7 | P | 59 | 7 | LSC | IGS (*psbA_rps19*) |
| *Calanthe ecarinata* | 48 | 30331 | P | 48 | 30331 | LSC | IGS (*petN_psbM*) |
| *Calanthe ecarinata* | 57 | 65005 | P | 57 | 65005 | LSC | IGS (*petA­_psbJ*) |
| *Calanthe ecarinata* | 55 | 49301 | P | 55 | 49301 | LSC | IGS (*trnf-GAA_ndhJ*) |
| *Calanthe ecarinata* | 35 | 55802 | F | 35 | 55819 | LSC | IGS (*atpB_rbcL*) |
| *Calanthe ecarinata* | 33 | 6556 | R | 33 | 6556 | LSC | IGS (*rps16_trnQ-UUG*) |
| *Calanthe ecarinata* | 39 | 3614 | R | 39 | 3614 | LSC | *trnK-UUU* |
| *Calanthe ecarinata* | 39 | 44503 | F | 39 | 102363 | LSC, IR | *ycf3*, *rps12* |
| *Calanthe ecarinata* | 39 | 44503 | P | 39 | 143220 | LSC, IR | *ycf3*, IGS (*trnV-GAC_rps12*) |
| *Calanthe ecarinata* | 32 | 28395 | P | 32 | 28395 | LSC | IGS (*rpoB_trnC-GCA*) |
| *Calanthe ecarinata* | 36 | 29374 | P | 36 | 29374 | LSC | IGS (*trnC-GCA_petN*) |
| *Calanthe ecarinata* | 36 | 47367 | R | 36 | 47367 | LSC | IGS (*trnT-UGU_trnL-UAA*) |
| *Calanthe ecarinata* | 37 | 116841 | P | 37 | 116841 | SSC | *ycf1* |
| *Calanthe ecarinata* | 37 | 118211 | P | 37 | 118211 | SSC | IGS (*ycf1_rps15*) |
| *Calanthe ecarinata* | 34 | 120578 | P | 34 | 120578 | SSC | *ndhA* |
| *Calanthe ecarinata* | 30 | 8423 | P | 30 | 45947 | LSC | IGS (*psbI_trnS-GCU*), *trnS-GGA* |
| *Calanthe ecarinata* | 31 | 126387 | P | 31 | 126778 | SSC | *ndhD*, IGS (*ndhD_ccsA*) |
| *Calanthe ecarinata* | 33 | 4625 | R | 33 | 4629 | LSC | IGS (*trnK-UUU_rps16*) |
| *Calanthe ecarinata* | 30 | 37424 | P | 30 | 37424 | LSC | IGS (*trnG-UCC_trnfM-CAU*) |
| *Calanthe ecarinata* | 30 | 51225 | R | 30 | 51225 | LSC | IGS (*ndhC-trnV-UAC*) |
| *Calanthe ecarinata* | 30 | 102788 | R | 30 | 102788 | IR | IGS (*rps12_trnV-GAC*) |
| *Calanthe ecarinata* | 30 | 102788 | C | 30 | 142804 | IR | IGS (*rps12_trnV-GAC*) |
| *Calanthe ecarinata* | 30 | 117017 | P | 30 | 117017 | SSC | *ycf1* |
| *Calanthe ecarinata* | 30 | 142804 | R | 30 | 142804 | IR | IGS (*rps12_trnV-GAC*) |
| *Calanthe ecarinata* | 32 | 36589 | P | 32 | 45947 | LSC | IGS (*psbC_trnS-UGA*), *trnS-GGA* |
| *Calanthe ecarinata* | 32 | 55792 | F | 32 | 55914 | LSC | IGS (*atpB_rbcL*) |
| *Calanthe ecarinata* | 32 | 102771 | R | 32 | 102780 | IR | IGS (*rps12_trnV-GAC*) |
| *Calanthe ecarinata* | 32 | 102771 | C | 32 | 142810 | IR | IGS (*rps12_trnV-GAC*) |
| *Calanthe ecarinata* | 32 | 102780 | C | 32 | 142819 | IR | IGS (*rps12_trnV-GAC*) |
| *Calanthe ecarinata* | 32 | 142810 | R | 32 | 142819 | IR | IGS (*rps12_trnV-GAC*) |
| *Calanthe ecarinata* | 31 | 62276 | P | 31 | 73687 | LSC | IGS (*ycf4_cemA*), *clpP* |
| *Calanthe ecarinata* | 30 | 10340 | F | 30 | 37349 | LSC | *trnG-GCC*, *trnG-UCC* |
| *Calanthe ecarinata* | 30 | 32518 | R | 30 | 47513 | LSC | IGS (*trnT-UGU_trnL-UAA*), *trnE-UUU*, *trnC-GGU,* |
| *Calanthe ecarinata* | 30 | 39600 | F | 30 | 41824 | LSC | *psaB*, *psaA* |
| *Calanthe ecarinata* | 30 | 44515 | F | 30 | 102375 | LSC, IR | *ycf3*, IGS (*rps12_trnV-GAC*) |
| *Calanthe ecarinata* | 30 | 44515 | P | 30 | 143217 | LSC, IR | IGS (*rps12_trnV-GAC*) |
| *Calanthe ecarinata* | 30 | 102764 | C | 30 | 142833 | IR | IGS (*rps12_trnV-GAC*) |

Tandem repeats in *C. nipponica*

| **Species** | **Size** | **Location 1** | **Type** | **Size** | **Location 2** | **Region** | | **Gene region** |
| --- | --- | --- | --- | --- | --- | --- | --- | --- |
| *Calanthe nipponica* | 58 | 5126 | P | 58 | 5126 | LSC | *rps16* | |
| *Calanthe nipponica* | 59 | 20 | P | 59 | 20 | LSC | IGS (*psbA_rps19*) | |
| *Calanthe nipponica* | 48 | 30445 | P | 48 | 30445 | LSC | IGS (*petN_psbM*) | |
| *Calanthe nipponica* | 46 | 43229 | P | 46 | 43229 | LSC | IGS (*psbA_ycf3*) | |
| *Calanthe nipponica* | 38 | 51752 | F | 38 | 51772 | LSC | IGS (*ndhC_trnV-UAC*) | |
| *Calanthe nipponica* | 39 | 3698 | R | 39 | 3698 | LSC | *trnK-UUU* | |
| *Calanthe nipponica* | 39 | 44710 | F | 39 | 102656 | LSC, IR | *ycf3*, IGS (*rps12_trnV-GAC*) | |
| *Calanthe nipponica* | 39 | 44710 | P | 39 | 143469 | LSC, IR | *ycf3*, IGS (*rps12_trnV-GAC*) | |
| *Calanthe nipponica* | 32 | 28462 | P | 32 | 28462 | LSC | IGS (*rpoB_trnC-GCA*) | |
| *Calanthe nipponica* | 41 | 65061 | P | 41 | 65061 | LSC | IGS (*petA_psbJ*) | |
| *Calanthe nipponica* | 36 | 29509 | P | 36 | 29509 | LSC | IGS (*trnC-GCA_petN*) | |
| *Calanthe nipponica* | 37 | 125275 | P | 37 | 125275 | SSC | *ndhA* | |
| *Calanthe nipponica* | 37 | 127622 | P | 37 | 127622 | SSC | IGS (*rps15_ycf1*) | |
| *Calanthe nipponica* | 37 | 128992 | P | 37 | 128992 | SSC | *ycf1* | |
| *Calanthe nipponica* | 31 | 95262 | F | 31 | 95280 | IR | *ycf2* | |
| *Calanthe nipponica* | 31 | 95262 | P | 31 | 150853 | SSC, IR | *ycf2* | |
| *Calanthe nipponica* | 31 | 95280 | P | 31 | 150871 | IR | *ycf2* | |
| *Calanthe nipponica* | 31 | 150853 | F | 31 | 150871 | IR | *ycf2* | |
| *Calanthe nipponica* | 30 | 8472 | P | 30 | 46154 | LSC | IGS (*psbI_trnS-GCU*) | |
| *Calanthe nipponica* | 30 | 106046 | P | 30 | 106085 | IR | IGS (*rrn16_trnL-GAU*) | |
| *Calanthe nipponica* | 30 | 106046 | F | 30 | 140049 | IR | IGS (*rrn16_trnL-GAU*) | |
| *Calanthe nipponica* | 30 | 106085 | F | 30 | 140088 | IR | IGS (*rrn16_trnL-GAU*) | |
| *Calanthe nipponica* | 30 | 140049 | P | 30 | 140088 | IR | IGS (*rrn16_trnL-GAU*) | |
| *Calanthe nipponica* | 32 | 121455 | P | 32 | 121455 | SSC | IGS (*psaC_ndhE*) | |
| *Calanthe nipponica* | 31 | 65049 | P | 31 | 65083 | LSC | IGS (*petA_psbJ*) | |
| *Calanthe nipponica* | 33 | 56174 | F | 33 | 116516 | LSC,SSC | IGS (*atpB_rbcL, ndhF_rpl32*) | |
| *Calanthe nipponica* | 30 | 9291 | P | 30 | 9291 | LSC | IGS (*trnS-GCU_trnG-GCC*) | |
| *Calanthe nipponica* | 30 | 37636 | P | 30 | 37636 | LSC | IGS (*trnG-UCC_trnfM-CAU*) | |
| *Calanthe nipponica* | 30 | 103079 | F | 30 | 103111 | IR | IGS (*rps12_trnV-GAC*) | |
| *Calanthe nipponica* | 30 | 103079 | P | 30 | 143023 | IR | IGS (*rps12_trnV-GAC*) | |
| *Calanthe nipponica* | 30 | 103111 | P | 30 | 143055 | IR | IGS (*rps12_trnV-GAC*) | |
| *Calanthe nipponica* | 30 | 128823 | P | 30 | 128823 | SSC | *ycf1* | |
| *Calanthe nipponica* | 30 | 143023 | F | 30 | 143055 | IR | IGS (*rps12_trnV-GAC*) | |
| *Calanthe nipponica* | 32 | 36801 | P | 32 | 46154 | LSC | *trnS-UGA*, *trnS-GGA* | |
| *Calanthe nipponica* | 30 | 10428 | F | 30 | 37561 | LSC | IGS (*trnG-GCC_trnR-UCU*), *trnG-UCC* | |
| *Calanthe nipponica* | 30 | 39809 | F | 30 | 42033 | LSC | *psaB*, *psaA* | |
| *Calanthe nipponica* | 30 | 44722 | F | 30 | 102668 | LSC, IR | *ycf3*, IGS (*rps12_trnV-GAC*) | |
| *Calanthe nipponica* | 30 | 44722 | P | 30 | 143466 | LSC, IR | *ycf3*, IGS (*rps12_trnV-GAC*) | |
| *Calanthe nipponica* | 30 | 103057 | C | 30 | 143082 | IR | IGS (*rps12_trnV-GAC*) | |

Tandem repeats in *C. taibaishanensis*

| **Species** | **Size** | **Location 1** | **Type** | **Size** | **Location 2** | **Region** | **Gene Region** |
| --- | --- | --- | --- | --- | --- | --- | --- |
| *Calanthe taibaishanensis* | 59 | 24 | P | 59 | 24 | LSC | IGS (*rps19_psbA*) |
| *Calanthe taibaishanensis* | 48 | 30138 | P | 48 | 30138 | LSC | IGS (*petN_psbN*) |
| *Calanthe taibaishanensis* | 46 | 42912 | P | 46 | 42912 | LSC | IGS (*psaA_ycf3*) |
| *Calanthe taibaishanensis* | 34 | 124713 | P | 34 | 124713 | LSC | *ndhA* |
| *Calanthe taibaishanensis* | 39 | 44413 | F | 39 | 102128 | LSC | *ycf3*, IGS (*rps12_trnV-GAC*) |
| *Calanthe taibaishanensis* | 39 | 44413 | P | 39 | 142921 | LSC | *ycf3*, IGS (*trnV-GAC_rps12*) |
| *Calanthe taibaishanensis* | 32 | 28288 | P | 32 | 28288 | LSC | IGS (*rpoB_trnC-GCA*) |
| *Calanthe taibaishanensis* | 41 | 64498 | P | 41 | 64498 | LSC | IGS (*petA_psbJ*) |
| *Calanthe taibaishanensis* | 38 | 76410 | R | 38 | 76410 | LSC | IGS (*psbB_psbN*) |
| *Calanthe taibaishanensis* | 30 | 128289 | P | 30 | 128289 | LSC | *ycf1* |
| *Calanthe taibaishanensis* | 36 | 29357 | P | 36 | 29357 | LSC | IGS (*trnC-GCA_petN*) |
| *Calanthe taibaishanensis* | 37 | 127088 | P | 37 | 127088 | LSC | IGS (*rps15_ycf1*) |
| *Calanthe taibaishanensis* | 37 | 128458 | P | 37 | 128458 | LSC | *ycf1* |
| *Calanthe taibaishanensis* | 31 | 94725 | F | 31 | 94743 | IR | *ycf2, ycf2* |
| *Calanthe taibaishanensis* | 31 | 94725 | P | 31 | 150314 | IR | *ycf2, ycf2* |
| *Calanthe taibaishanensis* | 31 | 94743 | P | 31 | 150332 | IR | *ycf2, ycf2* |
| *Calanthe taibaishanensis* | 31 | 150314 | F | 31 | 150332 | IR | *ycf2, ycf2* |
| *Calanthe taibaishanensis* | 30 | 8285 | P | 30 | 45854 | LSC | IGS (*psbI_trnS-GCU*), *trnS-GGA* |
| *Calanthe taibaishanensis* | 34 | 51198 | R | 34 | 51202 | LSC | IGS (*ndhC_trnV-UAC*), IGS (*ndhC_trnV-UAC*) |
| *Calanthe taibaishanensis* | 31 | 64486 | P | 31 | 64520 | LSC | IGS (*petA_psbJ*), IGS (*petA_psbJ*) |
| *Calanthe taibaishanensis* | 30 | 9100 | P | 30 | 9100 | LSC | IGS (*trnS-GCU_trnG-GCC*) |
| *Calanthe taibaishanensis* | 30 | 37318 | P | 30 | 37318 | LSC | IGS (*trnG-UCC_trnfM-CAU*) |
| *Calanthe taibaishanensis* | 32 | 36483 | P | 32 | 45854 | LSC | IGS (*psbC_trnS-UGA*), *trnS-GGA* |
| *Calanthe taibaishanensis* | 31 | 3748 | R | 31 | 3756 | LSC | *trnK-UUU, trnK-UUU* |
| *Calanthe taibaishanensis* | 31 | 14699 | F | 31 | 14721 | LSC | IGS (*atpH_atpI*), IGS (*atpH_atpI*) |
| *Calanthe taibaishanensis* | 30 | 10243 | F | 30 | 37243 | LSC | *trnG-GCC*, *trnG-UCC* |
| *Calanthe taibaishanensis* | 30 | 39492 | F | 30 | 41716 | LSC | *psaB*, *psaA* |
| *Calanthe taibaishanensis* | 30 | 44425 | F | 30 | 102140 | LSC, IR | *ycf3*, IGS (*rps12_trnV-GAC*) |
| *Calanthe taibaishanensis* | 30 | 44425 | P | 30 | 142918 | LSC, IR | *ycf3*, IGS (*trnV-GAC_rps12*) |
| *Calanthe taibaishanensis* | 30 | 102529 | C | 30 | 142534 | IR | IGS (*rps12_trnV-GCA*), IGS (*rrn16_rps12*) |

Tandem repeats in *C. tricarinata*

| **Species** | **Size** | **Location 1** | **Type** | **Size** | **Location 2** | **Region** | **Gene Region** |
| --- | --- | --- | --- | --- | --- | --- | --- |
| *Calanthe tricarinata* | 59 | 7 | P | 59 | 7 | LSC | IGS (*rps19_psbA*) |
| *Calanthe tricarinata* | 48 | 30326 | P | 48 | 30326 | LSC | IGS (*petN_psbM*) |
| *Calanthe tricarinata* | 57 | 65013 | P | 57 | 65013 | LSC | IGS (*petA_psbJ*) |
| *Calanthe tricarinata* | 55 | 49294 | P | 55 | 49294 | LSC | IGS (*trnF-GAA_ndhJ*) |
| *Calanthe tricarinata* | 40 | 129199 | F | 40 | 129218 | LSC | IGS (*rpl32_ndhF*), IGS (*rpl32_ndhF*) |
| *Calanthe tricarinata* | 33 | 6557 | R | 33 | 6557 | LSC | IGS (*rps16_trnP-UUG*) |
| *Calanthe tricarinata* | 39 | 3614 | R | 39 | 3614 | LSC | *trnK-UUU* |
| *Calanthe tricarinata* | 39 | 44499 | F | 39 | 102355 | LSC’ IR | *ycf3*, IGS (*rps12_trnV-GAC* |
| *Calanthe tricarinata* | 39 | 44499 | P | 39 | 143234 | LSC, IR | *ycf3*, IGS (*rps12_trnV-GAC*) |
| *Calanthe tricarinata* | 32 | 28389 | P | 32 | 28389 | LSC | IGS (*rps12_trnV-GAC*) |
| *Calanthe tricarinata* | 36 | 29369 | P | 36 | 29369 | LSC | IGS (*trnC-GCA_petN*) |
| *Calanthe tricarinata* | 36 | 47362 | R | 36 | 47362 | LSC | IGS (*trnT-UGU_trnL-UAA*) |
| *Calanthe tricarinata* | 37 | 116833 | P | 37 | 116833 | LSC | *ycf1* |
| *Calanthe tricarinata* | 37 | 118203 | P | 37 | 118203 | LSC | IGS (*ycf1_rps15*) |
| *Calanthe tricarinata* | 34 | 120570 | P | 34 | 120570 | LSC | ndhA |
| *Calanthe tricarinata* | 30 | 8417 | P | 30 | 45942 | LSC | IGS (*psbL_trnS-GCU), trnS-GGA* |
| *Calanthe tricarinata* | 31 | 126383 | P | 31 | 126773 | LSC | IGS (*ndhD_ccsA*)) |
| *Calanthe tricarinata* | 33 | 4626 | R | 33 | 4630 | LSC | IGS (*trnK-UUU_rps16*), IGS (*trnK-UUU_rps16*) |
| *Calanthe tricarinata* | 30 | 37420 | P | 30 | 37420 | LSC | IGS (*trnG-UCC_trnfM-CAU*) |
| *Calanthe tricarinata* | 30 | 51218 | R | 30 | 51218 | LSC | IGS (*ndhC_trnV-UAC*) |
| *Calanthe tricarinata* | 30 | 102780 | R | 30 | 102780 | IR | IGS (*rps12_trnV-GAC*) |
| *Calanthe tricarinata* | 30 | 102780 | C | 30 | 142818 | IR | IGS (*rps12_trnV-GAC*), IGS (*trnV-GAC_rps12*) |
| *Calanthe tricarinata* | 30 | 117009 | P | 30 | 117009 | LSC | *ycf1* |
| *Calanthe tricarinata* | 30 | 142818 | R | 30 | 142818 | IR | IGS (*trnV-GAC_rps12*) |
| *Calanthe tricarinata* | 32 | 36585 | P | 32 | 45942 | LSC | IGS (*psbC_trnS-UGA*), *trnS-GGA* |
| *Calanthe tricarinata* | 32 | 55816 | F | 32 | 55921 | LSC | IGS (*atpB_rbcL*), IGS (*atpB_rbcL*) |
| *Calanthe tricarinata* | 32 | 102763 | R | 32 | 102772 | IR | IGS (*rps12_trnV-GAC*), IGS (*rps12_trnV-GAC*) |
| *Calanthe tricarinata* | 32 | 102763 | C | 32 | 142824 | IR | IGS (*rps12_trnV-GAC*), IGS (*trnV-GAC_rps12*) |
| *Calanthe tricarinata* | 32 | 102772 | C | 32 | 142833 | IR | IGS (*rps12_trnV-GAC*), IGS (*trnV-GAC_rps12*) |
| *Calanthe tricarinata* | 32 | 142824 | R | 32 | 142833 | IR | IGS (*trnV-GAC_rps12*), IGS (*trnV-GAC_rps12*) |
| *Calanthe tricarinata* | 31 | 62283 | P | 31 | 73695 | LSC | IGS (*ycf4_cemA*), *clpP* |
| *Calanthe tricarinata* | 30 | 10334 | F | 30 | 37345 | LSC | *trnG-GCC*, *trnG-UCC* |
| *Calanthe tricarinata* | 30 | 32513 | R | 30 | 47508 | LSC | IGS (*trnE-UUC*_*trnT-GGU*), IGS (*trnT-UGU_trnL-UAA*) |
| *Calanthe tricarinata* | 30 | 39596 | F | 30 | 41820 | LSC | *psaB*, *psaA* |
| *Calanthe tricarinata* | 30 | 44511 | F | 30 | 102367 | LSC, IR | *ycf3*, IGS (*rps12_trnV-GAC*) |
| *Calanthe tricarinata* | 30 | 44511 | P | 30 | 143231 | LSC, IR | *ycf3*, IGS (*trnV-GAC_rps12*) |
| *Calanthe tricarinata* | 30 | 102756 | C | 30 | 142847 | IR | IGS (*rps12_trnV-GAC*), IGS (*trnV-GAC_rps12*) |

Table S3: Tandem repeats for *Phaius delavayi*

| **Species** | **Size** | **Location 1** | **Type** | **Size** | **Location 2** | **Region** | **Gene Region** |
| --- | --- | --- | --- | --- | --- | --- | --- |
| *Phaius delavayi* | 65 | 61451 | P | 65 | 61451 | LSC | IGS (*petA_psbJ*) |
| *Phaius delavayi* | 48 | 30020 | P | 48 | 30020 | LSC | IGS (*petN_psbM*) |
| *Phaius delavayi* | 37 | 124578 | F | 37 | 124615 | LSC | IGS (*rpl32_trnN-GUU*), IGS (*rpl32_trnN-GUU*) |
| *Phaius delavayi* | 41 | 98805 | R | 41 | 98805 | IR | IGS (*rps12_trnV-GAC*) |
| *Phaius delavayi* | 41 | 98805 | C | 41 | 134623 | IR | IGS (*rps12_trnV-GAC*), IGS (*trnV-GAC_rps12*), |
| *Phaius delavayi* | 41 | 134623 | R | 41 | 134623 | IR | IGS (*trnV-GAC_rps12*), |
| *Phaius delavayi* | 34 | 116489 | P | 34 | 116489 | LSC | *ndhA* |
| *Phaius delavayi* | 39 | 3674 | R | 39 | 3674 | LSC | *trnk-UUU* |
| *Phaius delavayi* | 39 | 43947 | F | 39 | 98390 | LSC, IR | *ycf3*, IGS (*rps12_trnV-GAC*), |
| *Phaius delavayi* | 39 | 43947 | P | 39 | 135040 | LSC, IR | *ycf3*, IGS (*trnV-GAC_rps12*) |
| *Phaius delavayi* | 32 | 28231 | P | 32 | 28231 | LSC | IGS (*rpoB_trnC-GCA*) |
| *Phaius delavayi* | 32 | 61780 | P | 32 | 61780 | LSC | IGS (*petA_psbJ*) |
| *Phaius delavayi* | 31 | 91223 | F | 31 | 91241 | IR | *ycf2*, *ycf2* |
| *Phaius delavayi* | 31 | 91223 | P | 31 | 142197 | IR | *ycf2*, *ycf2* |
| *Phaius delavayi* | 31 | 91241 | P | 31 | 142215 | IR | *ycf2*, *ycf2* |
| *Phaius delavayi* | 31 | 142197 | F | 31 | 142215 | IR | *ycf2*, *ycf2* |
| *Phaius delavayi* | 36 | 29163 | P | 36 | 29163 | LSC | IGS (*trnC-GCA_petN*) |
| *Phaius delavayi* | 37 | 112841 | P | 37 | 112841 | LSC | *ycf1* |
| *Phaius delavayi* | 37 | 114214 | P | 37 | 114214 | LSC | IGS (*ycf1_rps15*) |
| *Phaius delavayi* | 30 | 8420 | P | 30 | 45389 | LSC | IGS (*psbI_trnS-GCU*), *trnS-GGA* |
| *Phaius delavayi* | 32 | 98770 | F | 32 | 98784 | IR | IGS (*rps12_trnV-GAC*), IGS (*rps12_trnV-GAC*) |
| *Phaius delavayi* | 32 | 98770 | P | 32 | 134653 | IR | IGS (*rps12_trnV-GAC*), IGS (*trnV-GAC_rps12*) |
| *Phaius delavayi* | 32 | 98784 | P | 32 | 134667 | IR | IGS (*rps12_trnV-GAC*), IGS (*trnV-GAC_rps12*) |
| *Phaius delavayi* | 32 | 134653 | F | 32 | 134667 | IR | IGS (*trnV-GAC_rps12*), IGS (*trnV-GAC_rps12*) |
| *Phaius delavayi* | 31 | 61718 | F | 31 | 61751 | LSC | IGS (*petA_psbL*), IGS (*petA_psbL*) |
| *Phaius delavayi* | 31 | 98824 | R | 31 | 98824 | IR | IGS (*rps12_trnV-GAC*) |
| *Phaius delavayi* | 31 | 98824 | C | 31 | 134614 | IR | IGS (*rps12_trnV-GAC*), IGS (*trnV-GAC_rps12*) |
| *Phaius delavayi* | 31 | 134614 | R | 31 | 134614 | IR | IGS (*trnV-GAC_rps12*) |
| *Phaius delavayi* | 33 | 61773 | C | 33 | 61774 | LSC | IGS (*petA_psbL*), IGS (*petA_psbL*) |
| *Phaius delavayi* | 30 | 124643 | R | 30 | 124643 | LSC | IGS (*rpl32_trnN-GUU*) |
| *Phaius delavayi* | 32 | 36092 | P | 32 | 45389 | LSC | IGS (*psbC_trnS-UGA*), *trnS-GGA* |
| *Phaius delavayi* | 32 | 48694 | P | 32 | 48730 | LSC | IGS (*trnF-GAA_ndhJ*), IGS (*trnF-GAA_ndhJ*) |
| *Phaius delavayi* | 32 | 49385 | P | 32 | 53201 | LSC | IGS (*ndhJ_trnV-UAC*), IGS (*atpB_rbcL*) |
| *Phaius delavayi* | 31 | 1612 | P | 31 | 1612 | LSC | *trnK-UUU* |
| *Phaius delavayi* | 30 | 10265 | F | 30 | 36853 | LSC | *trnG-GCC*, *trnG-UCC* |
| *Phaius delavayi* | 30 | 29577 | F | 30 | 119564 | LSC | IGS (*petN-psbM*), IGS (*ndhE_psaC*) |
| *Phaius delavayi* | 30 | 39074 | F | 30 | 41298 | LSC | *psaB*, *psaA* |
| *Phaius delavayi* | 30 | 43959 | F | 30 | 98402 | LSC, IR | *ycf3*, IGS (*rps12_trnV-GAC*) |
| *Phaius delavayi* | 30 | 43959 | P | 30 | 135037 | LSC, IR | *ycf3*, IGS (*trnV-GAC_rps12*) |
| *Phaius delavayi* | 30 | 52768 | P | 30 | 77124 | LSC | IGS (*atpB_rbcL*), IGS (*petD_rpoA*) |

Table S3: Tandem repeats for *Phaius flavus*

| **Species** | **Size** | **Location 1** | **Type** | **Size** | **Location 2** | **Region** | **Gene Region** |
| --- | --- | --- | --- | --- | --- | --- | --- |
| *Phaius flavus* | 59 | 14 | P | 59 | 14 | LSC | IGS (*rps19_psbA*) |
| *Phaius flavus* | 46 | 29645 | P | 46 | 29645 | LSC | IGS (*trnC-GCA_petN*) |
| *Phaius flavus* | 48 | 30576 | P | 48 | 30576 | LSC | IGS (*petN_psbM*) |
| *Phaius flavus* | 46 | 43080 | P | 46 | 43080 |  | IGS (*psaA_ycf3*) |
| *Phaius flavus* | 39 | 3801 | R | 39 | 3801 | LSC | IGS (*matK_rps16*) |
| *Phaius flavus* | 39 | 44557 | F | 39 | 102466 | LSF | ycf3, IGS (*rps12_trnV-GAC*) |
| *Phaius flavus* | 39 | 44557 | P | 39 | 143267 | LSC | ycf3, IGS (*trnV-GAC_rps12*) |
| *Phaius flavus* | 32 | 28621 | P | 32 | 28621 | LSC | IGS (*rpoB_trnC-GCA*) |
| *Phaius flavus* | 34 | 125095 | P | 34 | 125095 | LSC | *ndhA* |
| *Phaius flavus* | 31 | 95094 | F | 31 | 95112 | IR | *ycf2*, *ycf2* |
| *Phaius flavus* | 31 | 95094 | P | 31 | 150629 | IR | *ycf2*, *ycf2* |
| *Phaius flavus* | 31 | 95112 | P | 31 | 150647 | IR | *ycf2*, *ycf2* |
| *Phaius flavus* | 31 | 150629 | F | 31 | 150647 | IR | *ycf2*, *ycf2* |
| *Phaius flavus* | 30 | 8658 | P | 30 | 45998 | LSC | IGS (*psbI_trnS-GCU*), *trnS-GGA* |
| *Phaius flavus* | 30 | 45675 | F | 30 | 67533 | LSC | IGS (*ycf3_trnS-GGA*), IGS (*psbE_petL*) |
| *Phaius flavus* | 30 | 51928 | P | 30 | 51928 | LSC | IGS (*ndhC_trnV-UAC*) |
| *Phaius flavus* | 32 | 36645 | P | 32 | 45998 | LSC | IGS (*psbC_trnS-GCU*), *trnS-GGA* |
| *Phaius flavus* | 31 | 6668 | F | 31 | 102994 | LSC, IR | IGS (*rps16_trnQ-UUG*), IGS (*rps12_trnV-GAC*) |
| *Phaius flavus* | 31 | 6668 | P | 31 | 142747 | LSC, IR | IGS (*rps16_trnQ-UUG*), IGS (*trnV-GAC_rps12*) |
| *Phaius flavus* | 30 | 6635 | P | 30 | 85605 | LSC | IGS (*rps16_trnQ-UUG*), *rpI16* |
| *Phaius flavus* | 30 | 10606 | F | 30 | 37409 | LSC | *trnG-GCC*, *trnG-UCC* |
| *Phaius flavus* | 30 | 39661 | F | 30 | 41885 | LSC | *psaB*, *psaA* |
| *Phaius flavus* | 30 | 44569 | F | 30 | 102478 | LSC, IR | *ycf3*, IGS (*rps12_trnV-GAC*) |
| *Phaius flavus* | 30 | 44569 | P | 30 | 143264 | LSC, IR | ycf3, IGS (*rrn16_rps12*) |
| *Phaius flavus* | 30 | 91144 | F | 30 | 91162 | IR | *ycf2*, *ycf2* |
| *Phaius flavus* | 30 | 91144 | P | 30 | 154580 | IR | *ycf2*, *ycf2* |
| *Phaius flavus* | 30 | 91162 | P | 30 | 154598 | IR | *ycf2*, *ycf2* |
| *Phaius flavus* | 30 | 154580 | F | 30 | 154598 | IR | *ycf2* |

Table S3: SSRs in *Calanthe alpina*

| **ID** | **SSR nr.** | **SSR type** | **SSR** | **size** | **start** | **end** | **Region** | **Gene Region** |
| --- | --- | --- | --- | --- | --- | --- | --- | --- |
| *Calanthe alpina* | 1 | p1 | (T)11 | 11 | 1391 | 1401 | LSC | IGS (*psbA_trnK-UUU*) |
| *Calanthe alpina* | 2 | p3 | (AAT)4 | 12 | 3470 | 3481 | LSC | *trnK-UUU* |
| *Calanthe alpina* | 3 | c | (TCTA)3 | 33 | 5103 | 5135 | LSC | *rps16* |
| *Calanthe alpina* | 4 | p2 | (AT)5 | 10 | 5307 | 5316 | LSC | rps16 |
| *Calanthe alpina* | 5 | c | (A)10 | 84 | 6289 | 6372 | LSC | IGS (*rps16_trnQ-UUG*) |
| *Calanthe alpina* | 6 | p1 | (T)10 | 10 | 6670 | 6679 | LSC | IGS (*rps16_trnQ-UUG*) |
| *Calanthe alpina* | 7 | c | (TAA)5 | 36 | 6830 | 6865 | LSC | IGS (*rps16_trnQ-UUG*) |
| *Calanthe alpina* | 8 | c | (T)10 | 101 | 7998 | 8098 | LSC | IGS (*psbK_psbI*) |
| *Calanthe alpina* | 9 | p1 | (A)10 | 10 | 10152 | 10161 | LSC | IGS (*trnR-UCU_atpA*) |
| *Calanthe alpina* | 10 | p1 | (A)14 | 14 | 14416 | 14429 | LSC | IGS (*atpH_atpI*) |
| *Calanthe alpina* | 11 | c | (T)10 | 117 | 18318 | 18434 | LSC | *rpoC2* |
| *Calanthe alpina* | 12 | p2 | (AT)5 | 10 | 19815 | 19824 | LSC | *rpoC2* |
| *Calanthe alpina* | 13 | p1 | (A)10 | 10 | 27979 | 27988 | LSC | IGS (*rpoB_trnC-GCA*) |
| *Calanthe alpina* | 14 | c | (AT)7 | 27 | 31868 | 31894 | LSC | IGS (*trnE-UUC_trnT-GGU*) |
| *Calanthe alpina* | 15 | p1 | (A)10 | 10 | 44382 | 44391 | LSC | *ycf3* |
| *Calanthe alpina* | 16 | p1 | (A)11 | 11 | 45361 | 45371 | LSC | IGS (*trnS-GGA_rps4*) |
| *Calanthe alpina* | 17 | p1 | (A)11 | 11 | 46134 | 46144 | LSC | IGS (*rps4_trnT-UGU*) |
| *Calanthe alpina* | 18 | p1 | (A)11 | 11 | 46777 | 46787 | LSC | IGS (*trnT-UGU_trnL-UAA*) |
| *Calanthe alpina* | 19 | p1 | (T)10 | 10 | 47789 | 47798 | LSC | IGS (*trnL-UAA-trnF-GAA*) |
| *Calanthe alpina* | 20 | p2 | (AT)6 | 12 | 47937 | 47948 | LSC | IGS (*trnL-UAA-trnF-GAA*) |
| *Calanthe alpina* | 21 | p1 | (T)14 | 14 | 48456 | 48469 | LSC | IGS (*trnF-GAA_ndhJ*) |
| *Calanthe alpina* | 22 | p1 | (T)11 | 11 | 49868 | 49878 | LSC | *ndhK* |
| *Calanthe alpina* | 23 | p1 | (T)10 | 10 | 50470 | 50479 | LSC | IGS (*ndhC_trnV-UAC*) |
| *Calanthe alpina* | 24 | c | (CTA)4 | 37 | 51575 | 51611 | LSC | IGS (*ndhC_trnV-UAC*) |
| *Calanthe alpina* | 25 | p2 | (TA)10 | 20 | 55091 | 55110 | LSC | IGS (*atpB_rbcL*) |
| *Calanthe alpina* | 26 | c | (TATT)3 | 121 | 55245 | 55365 | LSC | IGS (*atpB_rbcL*) |
| *Calanthe alpina* | 27 | p1 | (T)12 | 12 | 61269 | 61280 | LSC | IGS (*ycf4-cemA*) |
| *Calanthe alpina* | 28 | p4 | (AATG)3 | 12 | 62194 | 62205 | LSC | *cemA* |
| *Calanthe alpina* | 29 | p1 | (T)13 | 13 | 63440 | 63452 | LSC | IGS (*petA-psbJ*) |
| *Calanthe alpina* | 30 | p2 | (TA)5 | 10 | 63890 | 63899 | LSC | IGS (*petA-psbJ*) |
| *Calanthe alpina* | 31 | p2 | (TA)8 | 16 | 66672 | 66687 | LSC | IGS (*trnW-CCA_trnP-UGG*) |
| *Calanthe alpina* | 32 | p4 | (ATTA)3 | 12 | 67477 | 67488 | LSC | IGS (*trnW-CCA_trnP-UGG*) |
| *Calanthe alpina* | 33 | p2 | (TG)5 | 10 | 67977 | 67986 | LSC | IGS (*rpl33_rps18*) |
| *Calanthe alpina* | 34 | p1 | (T)11 | 11 | 68736 | 68746 | LSC | IGS (*rps18_rpl20*) |
| *Calanthe alpina* | 35 | p1 | (T)10 | 10 | 71445 | 71454 | LSC | *clpP* |
| *Calanthe alpina* | 36 | p1 | (T)11 | 11 | 71692 | 71702 | LSC | *clpP* |
| *Calanthe alpina* | 37 | p1 | (T)10 | 10 | 72037 | 72046 | LSC | *clpP* |
| *Calanthe alpina* | 38 | p2 | (TA)6 | 12 | 72546 | 72557 | LSC | IGS (*clpP_psbB*) |
| *Calanthe alpina* | 39 | p5 | (TATTC)3 | 15 | 72707 | 72721 | LSC | IGS (*clpP_psbB*) |
| *Calanthe alpina* | 40 | p2 | (TA)6 | 12 | 75263 | 75274 | LSC | IGS (*psbB_psbT*) |
| *Calanthe alpina* | 41 | p1 | (A)10 | 10 | 76350 | 76359 | LSC | *petB* |
| *Calanthe alpina* | 42 | p1 | (A)10 | 10 | 76599 | 76608 | LSC | *petB* |
| *Calanthe alpina* | 43 | p1 | (T)13 | 13 | 77999 | 78011 | LSC | *petD* |
| *Calanthe alpina* | 44 | c | (T)16 | 27 | 81066 | 81092 | LSC | IGS (*rpl36_infA*) |
| *Calanthe alpina* | 45 | p1 | (T)14 | 14 | 81975 | 81988 | LSC | IGS (*rps8_rpl14*) |
| *Calanthe alpina* | 46 | p2 | (TA)5 | 10 | 82126 | 82135 | LSC | IGS (*rps8_rpl14*) |
| *Calanthe alpina* | 47 | c | (T)10 | 33 | 82615 | 82647 | LSC | IGS (*rpl14_rpl16*) |
| *Calanthe alpina* | 48 | p1 | (T)12 | 12 | 84229 | 84240 | LSC | *rpl16* |
| *Calanthe alpina* | 49 | p2 | (GA)5 | 10 | 91526 | 91535 | IR | *ycf2* |
| *Calanthe alpina* | 50 | p1 | (T)10 | 10 | 101731 | 101740 | IR | IGS (*rps12_trnV-GAC*) |
| *Calanthe alpina* | 51 | c | (T)12 | 40 | 111949 | 111988 | LSC | *ycf1* |
| *Calanthe alpina* | 52 | p1 | (A)10 | 10 | 112112 | 112121 | LSC | *ycf1* |
| *Calanthe alpina* | 53 | c | (A)14 | 120 | 112229 | 112348 | LSC | *ycf1* |
| *Calanthe alpina* | 54 | p1 | (T)10 | 10 | 113568 | 113577 | LSC | *ycf1* |
| *Calanthe alpina* | 55 | p1 | (A)10 | 10 | 115494 | 115503 | LSC | *ycf1* |
| *Calanthe alpina* | 56 | p1 | (T)10 | 10 | 118945 | 118954 | LSC | *ndhA* |
| *Calanthe alpina* | 57 | p1 | (T)13 | 13 | 119417 | 119429 | LSC | *ndhA* |
| *Calanthe alpina* | 58 | p5 | (ATAAT)3 | 15 | 121836 | 121850 | LSC | *nad6* |
| *Calanthe alpina* | 59 | p4 | (ATCA)3 | 12 | 122299 | 122310 | LSC | *ndhE* |
| *Calanthe alpina* | 60 | p3 | (TTA)4 | 12 | 122462 | 122473 | LSC | IGS (*ndhE_phaC*) |
| *Calanthe alpina* | 61 | p1 | (A)10 | 10 | 124878 | 124887 | LSC | IGS (*ndhD_ccsA*) |
| *Calanthe alpina* | 62 | p1 | (A)14 | 14 | 127578 | 127591 | LSC | IGS (*rpl32_ndhF*) |
| *Calanthe alpina* | 63 | p1 | (A)10 | 10 | 140341 | 140350 | LSC | IGS (*trnV-GAC_rps12*) |
| *Calanthe alpina* | 64 | p2 | (TC)5 | 10 | 150546 | 150555 | IR | *ycf2* |

Table S3: SSRs in *Calanthe brevicornu*

| **ID** | **SSR nr.** | **SSR type** | **SSR** | **size** | **start** | **end** | **Region** | **Gene Region** |
| --- | --- | --- | --- | --- | --- | --- | --- | --- |
| *Calanthe_brevicornu* | 1 | p5 | (TCATA)3 | 15 | 90 | 104 | LSC | IGS (*rps19_psbA*) |
| *Calanthe_brevicornu* | 2 | p1 | (T)14 | 14 | 1439 | 1452 | LSC | IGS (*psbA_trnK-UUU*) |
| *Calanthe_brevicornu* | 3 | p3 | (AAT)4 | 12 | 3428 | 3439 | LSC | *trnK-UUU* |
| *Calanthe_brevicornu* | 4 | c | (A)10 | 67 | 4473 | 4539 | LSC | IGS (*trnK-UUU_rps16*) |
| *Calanthe_brevicornu* | 5 | c | (TCTA)3 | 49 | 5076 | 5124 | LSC | *rps16* |
| *Calanthe_brevicornu* | 6 | p2 | (TA)6 | 12 | 5301 | 5312 | LSC | *rps16* |
| *Calanthe_brevicornu* | 7 | p1 | (A)10 | 10 | 6886 | 6895 | LSC | IGS (*rps16_trnQ-UUG*) |
| *Calanthe_brevicornu* | 8 | c | (T)10 | 106 | 8027 | 8132 | LSC | IGS (*psbK_psbI*) |
| *Calanthe_brevicornu* | 9 | c | (A)10 | 93 | 9382 | 9474 | LSC | IGS (*trnS-GCU_trnG-GCC*) |
| *Calanthe_brevicornu* | 10 | p1 | (A)12 | 12 | 10490 | 10501 | LSC | IGS (*trnG-GCC_trnR-UCU*) |
| *Calanthe_brevicornu* | 11 | p4 | (GTCT)3 | 12 | 11759 | 11770 | LSC | *atpA* |
| *Calanthe_brevicornu* | 12 | p1 | (T)10 | 10 | 16773 | 16782 | LSC | IGS (*rps2_rpoC2*) |
| *Calanthe_brevicornu* | 13 | c | (T)10 | 117 | 18872 | 18988 | LSC | *rpoC2* |
| *Calanthe_brevicornu* | 14 | p2 | (AT)5 | 10 | 20357 | 20366 | LSC | *rpoC2* |
| *Calanthe_brevicornu* | 15 | p4 | (TTTA)3 | 12 | 28700 | 28711 | LSC | IGS (*rpoB_trnC-GCA*) |
| *Calanthe_brevicornu* | 16 | c | (TA)5 | 36 | 32595 | 32630 | LSC | IGS (*trnE-UUC_trnT-GGU*) |
| *Calanthe_brevicornu* | 17 | p1 | (T)10 | 10 | 32868 | 32877 | LSC | IGS (*trnE-UUC_trnT-GGU*) |
| *Calanthe_brevicornu* | 18 | p1 | (A)14 | 14 | 33256 | 33269 | LSC | IGS (*trnT-GGU_psbD*) |
| *Calanthe_brevicornu* | 19 | p4 | (TAAA)3 | 12 | 33464 | 33475 | LSC | IGS (*trnT-GGU_psbD*) |
| *Calanthe_brevicornu* | 20 | p1 | (T)12 | 12 | 43113 | 43124 | LSC | IGS (*psaA_ycf3*) |
| *Calanthe_brevicornu* | 21 | p1 | (T)10 | 10 | 45154 | 45163 | LSC | *ycf3* |
| *Calanthe_brevicornu* | 22 | p1 | (T)10 | 10 | 48625 | 48634 | LSC | IGS (*trnL-UAA_trnF-GAA*) |
| *Calanthe_brevicornu* | 23 | p2 | (AT)7 | 14 | 48773 | 48786 | LSC | IGS (*trnL-UAA_trnF-GAA*) |
| *Calanthe_brevicornu* | 24 | p1 | (T)10 | 10 | 50700 | 50709 | LSC | *ndhK* |
| *Calanthe_brevicornu* | 25 | p1 | (T)14 | 14 | 51301 | 51314 | LSC | IGS (*ndhC_trnV-UAC*) |
| *Calanthe_brevicornu* | 26 | c | (CTA)4 | 37 | 52480 | 52516 | LSC | IGS (*ndhC_trnV-UAC*) |
| *Calanthe_brevicornu* | 27 | c | (TCTATA)3 | 108 | 55919 | 56026 | LSC | IGS (*atpB_rbcL*) |
| *Calanthe_brevicornu* | 28 | p1 | (T)10 | 10 | 62733 | 62742 | LSC | IGS (*ycf4_cemA*) |
| *Calanthe_brevicornu* | 29 | p4 | (AATG)3 | 12 | 63656 | 63667 | LSC | *cemA* |
| *Calanthe_brevicornu* | 30 | p1 | (T)10 | 10 | 64902 | 64911 | LSC | IGS (*petA_psbJ*) |
| *Calanthe_brevicornu* | 31 | p4 | (ATTA)3 | 12 | 69276 | 69287 | LSC | IGS (*psaJ_rpl33*) |
| *Calanthe_brevicornu* | 32 | p2 | (TG)5 | 10 | 69785 | 69794 | LSC | IGS (*rpl33_rps18*) |
| *Calanthe_brevicornu* | 33 | p4 | (AAAT)3 | 12 | 70295 | 70306 | LSC | IGS (*rps18_rpl20*) |
| *Calanthe_brevicornu* | 34 | p1 | (T)10 | 10 | 70583 | 70592 | LSC | IGS (*rps18_rpl20*) |
| *Calanthe_brevicornu* | 35 | p1 | (T)11 | 11 | 73280 | 73290 | LSC | *clpP* |
| *Calanthe_brevicornu* | 36 | p1 | (T)10 | 10 | 76495 | 76504 | LSC | IGS (*psbB_psbT*) |
| *Calanthe_brevicornu* | 37 | c | (AT)6 | 87 | 76906 | 76992 | LSC | IGS (*psbB_psbT*) |
| *Calanthe_brevicornu* | 38 | p1 | (A)10 | 10 | 78118 | 78127 | LSC | *petB* |
| *Calanthe_brevicornu* | 39 | c | (TAGAA)3 | 69 | 79706 | 79774 | LSC | *petD* |
| *Calanthe_brevicornu* | 40 | p1 | (T)11 | 11 | 82866 | 82876 | LSC | IGS (*rpl36_infA*) |
| *Calanthe_brevicornu* | 41 | p2 | (GA)5 | 10 | 93130 | 93139 | IR | *ycf2* |
| *Calanthe_brevicornu* | 42 | p1 | (A)10 | 10 | 111017 | 111026 | IR | IGS (*rrn4.5_rrn5*) |
| *Calanthe_brevicornu* | 43 | p1 | (T)10 | 10 | 115718 | 115727 | LSC | IGS (*ndhF_rpl32*) |
| *Calanthe_brevicornu* | 44 | c | (TA)5 | 87 | 116312 | 116398 | LSC | IGS (*ndhF_rpl32*) |
| *Calanthe_brevicornu* | 45 | p1 | (A)12 | 12 | 119057 | 119068 | LSC | IGS (*ccsA_ndhD*) |
| *Calanthe_brevicornu* | 46 | p3 | (AAT)4 | 12 | 121376 | 121387 | LSC | IGS (*psaC_ndhE*) |
| *Calanthe_brevicornu* | 47 | p4 | (TTGA)3 | 12 | 121533 | 121544 | LSC | *ndhE* |
| *Calanthe_brevicornu* | 48 | p1 | (A)10 | 10 | 121976 | 121985 | LSC | IGS (*ndhE_nad6*) |
| *Calanthe_brevicornu* | 49 | p1 | (A)11 | 11 | 124896 | 124906 | LSC | *ndhA* |
| *Calanthe_brevicornu* | 50 | p1 | (T)10 | 10 | 128369 | 128378 | LSC | *ycf1* |
| *Calanthe_brevicornu* | 51 | p1 | (A)10 | 10 | 130301 | 130310 | LSC | *ycf1* |
| *Calanthe_brevicornu* | 52 | p1 | (T)14 | 14 | 131615 | 131628 | LSC | *ycf1* |
| *Calanthe_brevicornu* | 53 | c | (T)10 | 40 | 131869 | 131908 | LSC | *ycf1* |
| *Calanthe_brevicornu* | 54 | p1 | (T)10 | 10 | 134514 | 134523 | IR | IGS (*rrn5_rrn4.5*) |
| *Calanthe_brevicornu* | 55 | p2 | (TC)5 | 10 | 152401 | 152410 | IR | *ycf2* |

SSRs in *Calanthe nipponica*

| **ID** | **SSR nr.** | **SSR type** | **SSR** | **size** | **start** | **end** | **Region** | **Gene Region** |
| --- | --- | --- | --- | --- | --- | --- | --- | --- |
| *Calanthe nipponica* | 1 | p1 | (T)10 | 10 | 1420 | 1429 | LSC | IGS (*psbA_trnK-UUU*) |
| *Calanthe nipponica* | 2 | p3 | (ATA)4 | 12 | 3390 | 3401 | LSC | *trnK-UUU* |
| *Calanthe nipponica* | 3 | c | (TCTA)3 | 39 | 5136 | 5174 | LSC | *rps16* |
| *Calanthe nipponica* | 4 | p2 | (TA)5 | 10 | 5351 | 5360 | LSC | *rps16* |
| *Calanthe nipponica* | 5 | p1 | (A)10 | 10 | 6335 | 6344 | LSC | IGS (*rps16_trnQ-UUU*) |
| *Calanthe nipponica* | 6 | p5 | (ATCTT)3 | 15 | 8183 | 8197 | LSC | IGS (*psbK_psbI*) |
| *Calanthe nipponica* | 7 | p1 | (A)11 | 11 | 9475 | 9485 | LSC | IGS (*trnS-GCU_trnG-GCC*) |
| *Calanthe nipponica* | 8 | p4 | (GTCT)3 | 12 | 11837 | 11848 | LSC | *atpA* |
| *Calanthe nipponica* | 9 | p1 | (A)10 | 10 | 13139 | 13148 | LSC | *atpF* |
| *Calanthe nipponica* | 10 | p1 | (A)10 | 10 | 14687 | 14696 | LSC | IGS (*atpH_atpI*) |
| *Calanthe nipponica* | 11 | p1 | (T)12 | 12 | 16793 | 16804 | LSC | IGS (*rps2_rpoC2*) |
| *Calanthe nipponica* | 12 | c | (T)10 | 117 | 18894 | 19010 | LSC | *rpoC2* |
| *Calanthe nipponica* | 13 | p2 | (AT)5 | 10 | 20379 | 20388 | LSC | *rpoC2* |
| *Calanthe nipponica* | 14 | c | (AAAT)3 | 125 | 28679 | 28803 | LSC | IGS (*rpoB_trnC-GCA*) |
| *Calanthe nipponica* | 15 | p2 | (AT)6 | 12 | 32679 | 32690 | LSC | IGS (*trnE-UUC_trnT-GGU*) |
| *Calanthe nipponica* | 16 | p1 | (A)10 | 10 | 33209 | 33218 | LSC | IGS (*trnE-UUC_trnT-GGU*) |
| *Calanthe nipponica* | 17 | p1 | (A)10 | 10 | 33359 | 33368 | LSC | IGS (*trnT-GGU_psbD*) |
| *Calanthe nipponica* | 18 | p1 | (T)10 | 10 | 43273 | 43282 | LSC | IGS (*psaA_ycf3*) |
| *Calanthe nipponica* | 19 | p1 | (A)10 | 10 | 45365 | 45374 | LSC | *ycf3* |
| *Calanthe nipponica* | 20 | p1 | (A)10 | 10 | 47121 | 47130 | LSC | IGS (*rps4_trnT-UGU*) |
| *Calanthe nipponica* | 21 | p4 | (TATT)3 | 12 | 47606 | 47617 | LSC | IGS (*trnT-UGU_trnL-UAA*) |
| *Calanthe nipponica* | 22 | p1 | (A)10 | 10 | 47784 | 47793 | LSC | IGS (*trnT-UGU_trnL-UAA*) |
| *Calanthe nipponica* | 23 | p1 | (T)11 | 11 | 48779 | 48789 | LSC | IGS (*trnL-UAA_trnF-GAA*) |
| *Calanthe nipponica* | 24 | p1 | (T)10 | 10 | 50849 | 50858 | LSC | *ndhK* |
| *Calanthe nipponica* | 25 | c | (CTA)5 | 40 | 52561 | 52600 | LSC | IGS (*ndhC_trnV-UAC*) |
| *Calanthe nipponica* | 26 | p1 | (A)10 | 10 | 60299 | 60308 | LSC | *accD* |
| *Calanthe nipponica* | 27 | p1 | (T)10 | 10 | 64890 | 64899 | LSC | IGS (*petA_psbJ*) |
| *Calanthe nipponica* | 28 | p2 | (TA)7 | 14 | 65336 | 65349 | LSC | IGS (*petA_psbJ*) |
| *Calanthe nipponica* | 29 | p1 | (A)12 | 12 | 69203 | 69214 | LSC | IGS (*psaJ_rpl33*) |
| *Calanthe nipponica* | 30 | p4 | (ATTA)3 | 12 | 69385 | 69396 | LSC | IGS (*psaJ_rpl33*) |
| *Calanthe nipponica* | 31 | p2 | (TG)5 | 10 | 69883 | 69892 | LSC | IGS (*rpl33_rps18*) |
| *Calanthe nipponica* | 32 | p1 | (T)10 | 10 | 70648 | 70657 | LSC | IGS (*rps18_rpl20*) |
| *Calanthe nipponica* | 33 | p1 | (A)10 | 10 | 73320 | 73329 | LSC | *clpP* |
| *Calanthe nipponica* | 34 | p1 | (T)10 | 10 | 73943 | 73952 | LSC | *clpP* |
| *Calanthe nipponica* | 35 | p1 | (T)12 | 12 | 76768 | 76779 | LSC | IGS (*psbB_psbT*) |
| *Calanthe nipponica* | 36 | p2 | (TA)5 | 10 | 77187 | 77196 | LSC | IGS (*psbB_psbT*) |
| *Calanthe nipponica* | 37 | p1 | (T)13 | 13 | 79947 | 79959 | LSC | *petD* |
| *Calanthe nipponica* | 38 | p6 | (AGATAC)3 | 18 | 82790 | 82807 | LSC | IGS (*rps11_rpl36*) |
| *Calanthe nipponica* | 39 | p1 | (T)10 | 10 | 83048 | 83057 | LSC | IGS (*rpl36_infA*) |
| *Calanthe nipponica* | 40 | p1 | (T)10 | 10 | 83946 | 83955 | LSC | IGS (*rps8_rpl14*) |
| *Calanthe nipponica* | 41 | p2 | (GA)5 | 10 | 93464 | 93473 | IR | *ycf2* |
| *Calanthe nipponica* | 42 | p1 | (T)11 | 11 | 103743 | 103753 | IR | IGS (*rps12_trnV-GAC*) |
| *Calanthe nipponica* | 43 | p1 | (T)14 | 14 | 116537 | 116550 | LSC | IGS (*ndhF_rpl32*) |
| *Calanthe nipponica* | 44 | c* | (TAT)3 |  |  |  |  |  |

SSR in *Calanthe taibaishanensis*

| **ID** | **SSR nr.** | **SSR type** | **SSR** | **size** | **start** | **end** | **Region** | **Gene Region** |
| --- | --- | --- | --- | --- | --- | --- | --- | --- |
| *Calanthe taibaishanensis* | 1 | c | (A)10 | 31 | 1409 | 1439 | LSC | IGS (*psbA_trnK-UUU*) |
| *Calanthe taibaishanensis* | 2 | p3 | (ATA)4 | 12 | 3436 | 3447 | LSC | *trnK-UUU* |
| *Calanthe taibaishanensis* | 3 | c | (TCTA)3 | 32 | 5114 | 5145 | LSC | *rps16* |
| *Calanthe taibaishanensis* | 4 | p1 | (A)11 | 11 | 6158 | 6168 | LSC | IGS (*rps16_trnQ-UUG*) |
| *Calanthe taibaishanensis* | 5 | p1 | (A)10 | 10 | 6766 | 6775 | LSC | IGS (*rps16_trnQ-UUG*) |
| *Calanthe taibaishanensis* | 6 | c | (T)10 | 101 | 7910 | 8010 | LSC | IGS (*psbK_psbI*) |
| *Calanthe taibaishanensis* | 7 | c | (A)15 | 97 | 9279 | 9375 | LSC | IGS (*trnS-GCU_trnG-GCC*) |
| *Calanthe taibaishanensis* | 8 | p4 | (GTCT)3 | 12 | 11647 | 11658 | LSC | *atpA* |
| *Calanthe taibaishanensis* | 9 | c | (T)10 | 117 | 18722 | 18838 | LSC | *rpoC2* |
| *Calanthe taibaishanensis* | 10 | p2 | (AT)5 | 10 | 20207 | 20216 | LSC | *rpoC2* |
| *Calanthe taibaishanensis* | 11 | c | (TATAAG)3 | 87 | 28550 | 28636 | LSC | IGS (*rpoB_trnC-GCA*) |
| *Calanthe taibaishanensis* | 12 | p2 | (AT)6 | 12 | 32359 | 32370 | LSC | IGS (*trnE-UUC_trnT-GGU*) |
| *Calanthe taibaishanensis* | 13 | p1 | (A)12 | 12 | 32889 | 32900 | LSC | IGS (*trnE-UUC_trnT-GGU*) |
| *Calanthe taibaishanensis* | 14 | p1 | (A)10 | 10 | 33041 | 33050 | LSC | IGS (*trnT-GGU_psbD*) |
| *Calanthe taibaishanensis* | 15 | p1 | (T)10 | 10 | 38079 | 38088 | LSC | IGS (*rps14_psaB*) |
| *Calanthe taibaishanensis* | 16 | p1 | (T)10 | 10 | 42956 | 42965 | LSC | IGS (*psaA_ycf3*) |
| *Calanthe taibaishanensis* | 17 | p1 | (A)11 | 11 | 46801 | 46811 | LSC | IGS (*rps4_trnT-UGU*) |
| *Calanthe taibaishanensis* | 18 | p4 | (TATT)3 | 12 | 47287 | 47298 | LSC | IGS (*trnT-UGU_trnL-UAA*) |
| *Calanthe taibaishanensis* | 19 | c | (A)10 | 87 | 47465 | 47551 | LSC | IGS (*trnT-UGU_trnL-UAA*) |
| *Calanthe taibaishanensis* | 20 | p1 | (T)10 | 10 | 48462 | 48471 | LSC | IGS (*trnL-UAA_trnF-GAA*) |
| *Calanthe taibaishanensis* | 21 | p1 | (T)10 | 10 | 50315 | 50324 | LSC | *ndhK* |
| *Calanthe taibaishanensis* | 22 | p1 | (A)11 | 11 | 51900 | 51910 | LSC | IGS (*ndhC_trnV-UAC*) |
| *Calanthe taibaishanensis* | 23 | c | (CTA)5 | 40 | 52074 | 52113 | LSC | IGS (*ndhC_trnV-UAC*) |
| *Calanthe taibaishanensis* | 24 | p1 | (A)10 | 10 | 58325 | 58334 | LSC | IGS (*rbcL_accD*) |
| *Calanthe taibaishanensis* | 25 | p1 | (A)10 | 10 | 60247 | 60256 | LSC | IGS (*accD_psaI*) |
| *Calanthe taibaishanensis* | 26 | p1 | (T)10 | 10 | 62163 | 62172 | LSC | IGS (*ycf4_cemA*) |
| *Calanthe taibaishanensis* | 27 | p1 | (T)14 | 14 | 64323 | 64336 | LSC | IGS (*petA_psbJ*) |
| *Calanthe taibaishanensis* | 28 | p2 | (TA)6 | 12 | 64773 | 64784 | LSC | IGS (*petA_psbJ*) |
| *Calanthe taibaishanensis* | 29 | p1 | (A)11 | 11 | 66051 | 66061 | LSC | IGS (*psbE_petL*) |
| *Calanthe taibaishanensis* | 30 | p1 | (A)11 | 11 | 68646 | 68656 | LSC | IGS (*psaJ_rpl33*) |
| *Calanthe taibaishanensis* | 31 | p4 | (ATTA)3 | 12 | 68827 | 68838 | LSC | IGS (*psaJ_rpl33*) |
| *Calanthe taibaishanensis* | 32 | p2 | (TG)5 | 10 | 69321 | 69330 | LSC | IGS (*rpl33_rps18*) |
| *Calanthe taibaishanensis* | 33 | p1 | (T)11 | 11 | 70078 | 70088 | LSC | *rpl20* |
| *Calanthe taibaishanensis* | 34 | p1 | (T)11 | 11 | 72788 | 72798 | LSC | *clpP* |
| *Calanthe taibaishanensis* | 35 | p1 | (T)10 | 10 | 73893 | 73902 | LSC | IGS (*clpP_psbB*) |
| *Calanthe taibaishanensis* | 36 | p1 | (T)12 | 12 | 76210 | 76221 | LSC | IGS (*psbB_psbT*) |
| *Calanthe taibaishanensis* | 37 | p2 | (TA)5 | 10 | 76659 | 76668 | LSC | IGS (*psbB_psbT*) |
| *Calanthe taibaishanensis* | 38 | p1 | (A)10 | 10 | 77781 | 77790 | LSC | *petB* |
| *Calanthe taibaishanensis* | 39 | p1 | (A)10 | 10 | 78030 | 78039 | LSC | *petB* |
| *Calanthe taibaishanensis* | 40 | p1 | (T)12 | 12 | 79420 | 79431 | LSC | *petD* |
| *Calanthe taibaishanensis* | 41 | p1 | (T)11 | 11 | 82508 | 82518 | LSC | IGS (*rpl36_infA*) |
| *Calanthe taibaishanensis* | 42 | p2 | (GA)5 | 10 | 92927 | 92936 | IR | *ycf2* |
| *Calanthe taibaishanensis* | 43 | p1 | (T)11 | 11 | 103204 | 103214 | IR | IGS (*rps12_trnV-GAC*) |
| *Calanthe taibaishanensis* | 44 | p1 | (T)10 | 10 | 115965 | 115974 | LSC | IGS (*ndhF_rpl32*) |
| *Calanthe taibaishanensis* | 45 | p4 | (AATT)3 | 12 | 118714 | 118725 | LSC | IGS (*ccsA_ndhD*) |
| *Calanthe taibaishanensis* | 46 | p3 | (AAT)4 | 12 | 121093 | 121104 | LSC | IGS (*psaC_ndhE*) |
| *Calanthe taibaishanensis* | 47 | p4 | (TTGA)3 | 12 | 121255 | 121266 | LSC | *ndhE* |
| *Calanthe taibaishanensis* | 48 | p1 | (A)17 | 17 | 124612 | 124628 | LSC | *ndhA* |
| *Calanthe taibaishanensis* | 49 | p1 | (T)10 | 10 | 128109 | 128118 | LSC | *ycf1* |
| *Calanthe taibaishanensis* | 50 | p1 | (A)10 | 10 | 130041 | 130050 | LSC | *ycf1* |
| *Calanthe taibaishanensis* | 51 | p1 | (T)14 | 14 | 131355 | 131368 | LSC | *ycf1* |
| *Calanthe taibaishanensis* | 52 | p1 | (T)10 | 10 | 131476 | 131485 | LSC | *ycf1* |
| *Calanthe taibaishanensis* | 53 | c | (T)11 | 40 | 131609 | 131648 | LSC | *ycf1* |
| *Calanthe taibaishanensis* | 54 | p1 | (A)11 | 11 | 141875 | 141885 | IR | IGS (*trnV-GAC_rps12*) |
| *Calanthe taibaishanensis* | 55 | p2 | (TC)5 | 10 | 152153 | 152162 | IR | *ycf2* |

SSR in *Calanthe tricarinata*

| **ID** | **SSR nr.** | | **SSR type** | **SSR** | **size** | **start** | **end** | **Region** | **Gene Region** |
| --- | --- | --- | --- | --- | --- | --- | --- | --- | --- |
| *Calanthe tricarinata* | | 1 | p5 | (TCATA)3 | 15 | 74 | 88 | LSC | IGS (*rps19_psbA*) |
| *Calanthe tricarinata* | | 2 | p3 | (AAT)4 | 12 | 3426 | 3437 | LSC | *trnK-UUU* |
| *Calanthe tricarinata* | | 3 | p1 | (A)12 | 12 | 4525 | 4536 | LSC | IGS (*trnK-UUU_rps16*) |
| *Calanthe tricarinata* | | 4 | c | (TCTA)3 | 49 | 5073 | 5121 | LSC | *rps16* |
| *Calanthe tricarinata* | | 5 | p2 | (TA)5 | 10 | 5297 | 5306 | LSC | *rps16* |
| *Calanthe tricarinata* | | 6 | p5 | (ATCTT)3 | 15 | 8117 | 8131 | LSC | IGS (*psbK_psbI*) |
| *Calanthe tricarinata* | | 7 | p1 | (A)11 | 11 | 9381 | 9391 | LSC | IGS (*trnS-GCU_trnG-GCC*) |
| *Calanthe tricarinata* | | 8 | p4 | (GTCT)3 | 12 | 11763 | 11774 | LSC | *atpA* |
| *Calanthe tricarinata* | | 9 | p1 | (A)10 | 10 | 13065 | 13074 | LSC | *atpF* |
| *Calanthe tricarinata* | | 10 | p1 | (T)10 | 10 | 16731 | 16740 | LSC | IGS (*rps2_rpoC2*) |
| *Calanthe tricarinata* | | 11 | c | (T)10 | 117 | 18830 | 18946 | LSC | *rpoC2* |
| *Calanthe tricarinata* | | 12 | p2 | (AT)5 | 10 | 20315 | 20324 | LSC | *rpoC2* |
| *Calanthe tricarinata* | | 13 | p4 | (TTTA)3 | 12 | 28658 | 28669 | LSC | IGS (*rpoB_trnC-GCA*) |
| *Calanthe tricarinata* | | 14 | c | (TA)5 | 36 | 32545 | 32580 | LSC | IGS (*trnE-UUC_trnT-GGU*) |
| *Calanthe tricarinata* | | 15 | p1 | (T)10 | 10 | 32930 | 32939 | LSC | IGS (*trnE-UUC_trnT-GGU*) |
| *Calanthe tricarinata* | | 16 | p1 | (A)10 | 10 | 33207 | 33216 | LSC | IGS (*trnT-GGU_psbD*) |
| *Calanthe tricarinata* | | 17 | p4 | (TAAA)3 | 12 | 33411 | 33422 | LSC | IGS (*trnT-GGU_psbD*) |
| *Calanthe tricarinata* | | 18 | p1 | (T)12 | 12 | 43060 | 43071 | LSC | IGS (*psaA_ycf3*) |
| *Calanthe tricarinata* | | 19 | p1 | (A)10 | 10 | 45153 | 45162 | LSC | *ycf3* |
| *Calanthe tricarinata* | | 20 | p2 | (AT)7 | 14 | 48703 | 48716 | LSC | IGS (*trnL-UAA_trnF-GAA*) |
| *Calanthe tricarinata* | | 21 | p5 | (TATAT)3 | 15 | 49171 | 49185 | LSC | IGS (*trnF-GAA_ndhJ*) |
| *Calanthe tricarinata* | | 22 | p1 | (T)10 | 10 | 50630 | 50639 | LSC | *ndhK* |
| *Calanthe tricarinata* | | 23 | p1 | (T)13 | 13 | 51231 | 51243 | LSC | IGS (*ndhC_trnV-UAC*) |
| *Calanthe tricarinata* | | 24 | c | (CTA)4 | 37 | 52410 | 52446 | LSC | IGS (*ndhC_trnV-UAC*) |
| *Calanthe tricarinata* | | 25 | p2 | (TA)9 | 18 | 55939 | 55956 | LSC | IGS (*atpB_rbcL*) |
| *Calanthe tricarinata* | | 26 | p1 | (T)10 | 10 | 62687 | 62696 | LSC | IGS (*ycf4_cemA*) |
| *Calanthe tricarinata* | | 27 | p4 | (AATG)3 | 12 | 63602 | 63613 | LSC | *cemA* |
| *Calanthe tricarinata* | | 28 | p1 | (T)12 | 12 | 64848 | 64859 | LSC | IGS (*petA_psbJ*) |
| *Calanthe tricarinata* | | 29 | p1 | (T)10 | 10 | 67464 | 67473 | LSC | IGS (*psbE_petL*) |
| *Calanthe tricarinata* | | 30 | p4 | (ATTA)3 | 12 | 69225 | 69236 | LSC | IGS (*psaJ_rpl33*) |
| *Calanthe tricarinata* | | 31 | p2 | (TG)5 | 10 | 69734 | 69743 | LSC | IGS (*rpl33_rps18*) |
| *Calanthe tricarinata* | | 32 | p4 | (AAAT)3 | 12 | 70244 | 70255 | LSC | IGS (*rps18_rps20*) |
| *Calanthe tricarinata* | | 33 | p1 | (T)11 | 11 | 73228 | 73238 | LSC | *clpP* |
| *Calanthe tricarinata* | | 34 | p1 | (T)10 | 10 | 73477 | 73486 | LSC | *clpP* |
| *Calanthe tricarinata* | | 35 | c | (AT)7 | 89 | 76840 | 76928 | LSC | IGS (*psbB_psbT*) |
| *Calanthe tricarinata* | | 36 | p1 | (A)10 | 10 | 78054 | 78063 | LSC | *petB* |
| *Calanthe tricarinata* | | 37 | c | (TAGAA)3 | 68 | 79642 | 79709 | LSC | *petD* |
| *Calanthe tricarinata* | | 38 | p2 | (GA)5 | 10 | 93180 | 93189 | IR | *ycf2* |
| *Calanthe tricarinata* | | 39 | p1 | (A)10 | 10 | 111066 | 111075 | IR | IGS (*rrn4.5_rrn5*) |
| *Calanthe tricarinata* | | 40 | c | (T)12 | 40 | 113681 | 113720 | LSC | *ycf1* |
| *Calanthe tricarinata* | | 41 | p1 | (A)14 | 14 | 113961 | 113974 | LSC | *ycf1* |
| *Calanthe tricarinata* | | 42 | p1 | (T)10 | 10 | 115279 | 115288 | LSC | *ycf1* |
| *Calanthe tricarinata* | | 43 | p1 | (A)10 | 10 | 117211 | 117220 | LSC | *ycf1* |
| *Calanthe tricarinata* | | 44 | p2 | (AG)5 | 10 | 119216 | 119225 | LSC | *ndhH* |
| *Calanthe tricarinata* | | 45 | p1 | (T)10 | 10 | 120682 | 120691 | LSC | *ndhA* |
| *Calanthe tricarinata* | | 46 | p6 | (CATATG)4 | 24 | 121271 | 121294 | LSC | *ndhA* |
| *Calanthe tricarinata* | | 47 | p4 | (ATCA)3 | 12 | 124047 | 124058 | LSC | *ndhE* |
| *Calanthe tricarinata* | | 48 | p3 | (TTA)4 | 12 | 124203 | 124214 | LSC | IGS (*ndhE_psaC*) |
| *Calanthe tricarinata* | | 49 | p1 | (T)11 | 11 | 126524 | 126534 | LSC | IGS (*ndhD_ccsA*) |
| *Calanthe tricarinata* | | 50 | p2 | (AT)5 | 10 | 129185 | 129194 | LSC | IGS (*rpl32_ndhF*) |
| *Calanthe tricarinata* | | 51 | p1 | (T)10 | 10 | 134554 | 134563 | IR | IGS (*rrn5_rrn4.5*) |
| *Calanthe tricarinata* | | 52 | p2 | (TC)5 | 10 | 152440 | 152449 | IR | *ycf2* |

SSR in *Calanthe ecarinata*

| **ID** | **SSR nr.** | **SSR type** | **SSR** | **size** | **start** | **end** | **Region** | **Gene Region** |
| --- | --- | --- | --- | --- | --- | --- | --- | --- |
| *Calanthe ecarinata* | 1 | p5 | (TCATA)3 | 15 | 74 | 88 | LSC | IGS (*rps19_psbA*) |
| *Calanthe ecarinata* | 2 | p3 | (AAT)4 | 12 | 3426 | 3437 | LSC | *trnK-UUU* |
| *Calanthe ecarinata* | 3 | p1 | (A)12 | 12 | 4524 | 4535 | LSC | IGS (*trnK-UUU_rps16*) |
| *Calanthe ecarinata* | 4 | c | (TCTA)3 | 49 | 5072 | 5120 | LSC | *rps16* |
| *Calanthe ecarinata* | 5 | p2 | (TA)5 | 10 | 5296 | 5305 | LSC | *rps16* |
| *Calanthe ecarinata* | 6 | p5 | (ATCTT)3 | 15 | 8123 | 8137 | LSC | IGS (*psbK_psbI*) |
| *Calanthe ecarinata* | 7 | p1 | (A)11 | 11 | 9387 | 9397 | LSC | IGS (*trnS-GCU_trnG-GCC*) |
| *Calanthe ecarinata* | 8 | p4 | (GTCT)3 | 12 | 11769 | 11780 | LSC | *atpA* |
| *Calanthe ecarinata* | 9 | p1 | (A)10 | 10 | 13071 | 13080 | LSC | *atpF* |
| *Calanthe ecarinata* | 10 | p1 | (T)10 | 10 | 16737 | 16746 | LSC | IGS (*rps2_rpoC2*) |
| *Calanthe ecarinata* | 11 | c | (T)10 | 117 | 18836 | 18952 | LSC | *rpoC2* |
| *Calanthe ecarinata* | 12 | p2 | (AT)5 | 10 | 20321 | 20330 | LSC | *rpoC2* |
| *Calanthe ecarinata* | 13 | p4 | (TTTA)3 | 12 | 28664 | 28675 | LSC | IGS (*rpoB_trnC-GCA*) |
| *Calanthe ecarinata* | 14 | c | (TA)5 | 36 | 32550 | 32585 | LSC | IGS (*trnE-UUC_trnT-GGU*) |
| *Calanthe ecarinata* | 15 | p1 | (A)10 | 10 | 33211 | 33220 | LSC | IGS (*trnE-UUC_trnT-GGU*) |
| *Calanthe ecarinata* | 16 | p4 | (TAAA)3 | 12 | 33415 | 33426 | LSC | IGS (*trnT-GGU_psbD*) |
| *Calanthe ecarinata* | 17 | p1 | (T)12 | 12 | 43064 | 43075 | LSC | IGS (*trnT-GGU_psbD*) |
| *Calanthe ecarinata* | 18 | p1 | (A)11 | 11 | 45157 | 45167 | LSC | IGS (*psaA_ycf3*) |
| *Calanthe ecarinata* | 19 | p2 | (AT)8 | 16 | 48708 | 48723 | LSC | *ycf3* |
| *Calanthe ecarinata* | 20 | p5 | (TATAT)3 | 15 | 49178 | 49192 | LSC | IGS (*trnL-UAA_trnF-GAA*) |
| *Calanthe ecarinata* | 21 | p1 | (T)10 | 10 | 50637 | 50646 | LSC | IGS (*trnF-GAA_ndhJ*) |
| *Calanthe ecarinata* | 22 | p1 | (T)13 | 13 | 51238 | 51250 | LSC | *ndhK* |
| *Calanthe ecarinata* | 23 | c | (CTA)4 | 37 | 52386 | 52422 | LSC | IGS (*ndhC_trnV-UAC*) |
| *Calanthe ecarinata* | 24 | p2 | (TA)9 | 18 | 55932 | 55949 | LSC | IGS (*ndhC_trnV-UAC*) |
| *Calanthe ecarinata* | 25 | p4 | (AATG)3 | 12 | 63594 | 63605 | LSC | IGS (*atpB_rbcL*) |
| *Calanthe ecarinata* | 26 | p1 | (T)12 | 12 | 64840 | 64851 | LSC | IGS (*ycf4_cemA*) |
| *Calanthe ecarinata* | 27 | p1 | (T)10 | 10 | 67456 | 67465 | LSC | *cemA* |
| *Calanthe ecarinata* | 28 | p4 | (ATTA)3 | 12 | 69217 | 69228 | LSC | IGS (*petA_psbJ*) |
| *Calanthe ecarinata* | 29 | p2 | (TG)5 | 10 | 69726 | 69735 | LSC | IGS (*psbE_petL*) |
| *Calanthe ecarinata* | 30 | p4 | (AAAT)3 | 12 | 70236 | 70247 | LSC | IGS (*psaJ_rpl33*) |
| *Calanthe ecarinata* | 31 | p1 | (T)11 | 11 | 73220 | 73230 | LSC | IGS (*rpl33_rps18*) |
| *Calanthe ecarinata* | 32 | p1 | (T)10 | 10 | 73469 | 73478 | LSC | *clpP* |
| *Calanthe ecarinata* | 33 | c | (AT)7 | 89 | 76850 | 76938 | LSC | IGS (*psbB_psbT*) |
| *Calanthe ecarinata* | 34 | p1 | (A)10 | 10 | 78064 | 78073 | LSC | *petB* |
| *Calanthe ecarinata* | 35 | c | (TAGAA)4 | 74 | 79652 | 79725 | LSC | *petD* |
| *Calanthe ecarinata* | 36 | p1 | (A)10 | 10 | 85993 | 86002 | LSC | *rpl16* |
| *Calanthe ecarinata* | 37 | p2 | (GA)5 | 10 | 93188 | 93197 | IR | *ycf2* |
| *Calanthe ecarinata* | 38 | p1 | (A)10 | 10 | 111074 | 111083 | IR | IGS (rrn4.5_rrn5) |
| *Calanthe ecarinata* | 39 | c | (T)12 | 40 | 113689 | 113728 | LSC | *ycf1* |
| *Calanthe ecarinata* | 40 | p1 | (A)14 | 14 | 113969 | 113982 | LSC | *ycf1* |
| *Calanthe ecarinata* | 41 | p1 | (T)10 | 10 | 115287 | 115296 | LSC | *ycf1* |
| *Calanthe ecarinata* | 42 | p1 | (A)10 | 10 | 117219 | 117228 | LSC | *ycf1* |
| *Calanthe ecarinata* | 43 | p2 | (AG)5 | 10 | 119224 | 119233 | LSC | *ndhH* |
| *Calanthe ecarinata* | 44 | p1 | (T)11 | 11 | 120690 | 120700 | LSC | *ndhA* |
| *Calanthe ecarinata* | 45 | p6 | (CATATG)3 | 18 | 121280 | 121297 | LSC | *ndhA* |
| *Calanthe ecarinata* | 46 | p1 | (T)10 | 10 | 123611 | 123620 | LSC | *ndhE* |
| *Calanthe ecarinata* | 47 | p4 | (ATCA)3 | 12 | 124051 | 124062 | LSC | *ndhE* |
| *Calanthe ecarinata* | 48 | p3 | (TTA)4 | 12 | 124207 | 124218 | LSC | IGS (*ndhE_psaC*) |
| *Calanthe ecarinata* | 49 | p1 | (T)12 | 12 | 126528 | 126539 | LSC | IGS (*ndhD_ccsA*) |
| *Calanthe ecarinata* | 50 | p2 | (AT)5 | 10 | 129190 | 129199 | LSC | IGS (*rpl32_ndhF*) |
| *Calanthe ecarinata* | 51 | p1 | (T)10 | 10 | 134540 | 134549 | IR | IGS (*rrn5_rrn4.5*) |
| *Calanthe ecarinata* | 52 | p2 | (TC)5 | 10 | 152426 | 152435 | IR | *ycf2* |

SSRs in *Phaius delavayi*

| **ID** | **SSR nr.** | **SSR type** | **SSR** | **size** | **start** | **end** | **Region** | **Gene Region** |
| --- | --- | --- | --- | --- | --- | --- | --- | --- |
| *Phaius delavayi* | 1 | p1 | (T)10 | 10 | 1411 | 1420 | LSC | IGS (*psbA_trnK-UUU*) |
| *Phaius delavayi* | 2 | p1 | (T)10 | 10 | 1677 | 1686 | LSC | *trnK-UUU* |
| *Phaius delavayi* | 3 | c | (TCTA)3 | 36 | 5120 | 5155 | LSC | *rps16* |
| *Phaius delavayi* | 4 | p1 | (A)10 | 10 | 6901 | 6910 | LSC | IGS (*rps16_trnQ-UUG*) |
| *Phaius delavayi* | 5 | p1 | (T)10 | 10 | 8046 | 8055 | LSC | IGS (*psbK_psbI*) |
| *Phaius delavayi* | 6 | p1 | (A)10 | 10 | 10551 | 10560 | LSC | IGS (*trnR-UCU_atpA*) |
| *Phaius delavayi* | 7 | p4 | (GTCT)3 | 12 | 11676 | 11687 | LSC | *atpA* |
| *Phaius delavayi* | 8 | p1 | (T)10 | 10 | 13828 | 13837 | LSC | IGS (*atpF_atpH*) |
| *Phaius delavayi* | 9 | p1 | (T)10 | 10 | 16589 | 16598 | LSC | IGS (*rps2_rpoC2*) |
| *Phaius delavayi* | 10 | c | (T)10 | 117 | 18688 | 18804 | LSC | *rpoC2* |
| *Phaius delavayi* | 11 | p2 | (AT)5 | 10 | 20173 | 20182 | LSC | *rpoC2* |
| *Phaius delavayi* | 12 | p4 | (TTTA)3 | 12 | 28455 | 28466 | LSC | IGS (*rpoB_trnC-GCA*) |
| *Phaius delavayi* | 13 | p3 | (TAT)4 | 12 | 29586 | 29597 | LSC | IGS (*petN_psbM*) |
| *Phaius delavayi* | 14 | p4 | (AGAA)3 | 12 | 30498 | 30509 | LSC | IGS (*psbM_trnD-GUC*) |
| *Phaius delavayi* | 15 | p1 | (T)12 | 12 | 32624 | 32635 | LSC | IGS (*trnT-GGU_psbD*) |
| *Phaius delavayi* | 16 | p1 | (T)10 | 10 | 42538 | 42547 | LSC | IGS (*psaA_ycf3*) |
| *Phaius delavayi* | 17 | p1 | (A)10 | 10 | 46965 | 46974 | LSC | IGS (*trnT-UGU_ trnL-UAA*) |
| *Phaius delavayi* | 18 | p1 | (T)10 | 10 | 47961 | 47970 | LSC | IGS (*trnL-UAA_trnF-GAA*) |
| *Phaius delavayi* | 19 | p2 | (AT)7 | 14 | 48109 | 48122 | LSC | IGS (*trnL-UAA_trnF-GAA*) |
| *Phaius delavayi* | 20 | p3 | (CTA)4 | 12 | 49543 | 49554 | LSC | IGS (*ndhC_trnV-UAC*) |
| *Phaius delavayi* | 21 | p1 | (T)11 | 11 | 52783 | 52793 | LSC | IGS (*atpB_rbcL*) |
| *Phaius delavayi* | 22 | p2 | (TA)9 | 18 | 53016 | 53033 | LSC | IGS (*atpB_rbcL*) |
| *Phaius delavayi* | 23 | p1 | (T)11 | 11 | 59128 | 59138 | LSC | IGS (*ycf4_cemA*) |
| *Phaius delavayi* | 24 | p4 | (AATG)3 | 12 | 60044 | 60055 | LSC | *cemA* |
| *Phaius delavayi* | 25 | p1 | (T)12 | 12 | 61290 | 61301 | LSC | IGS (*petA_psbJ*) |
| *Phaius delavayi* | 26 | p2 | (AT)11 | 22 | 61786 | 61807 | LSC | IGS (*petA_psbJ*) |
| *Phaius delavayi* | 27 | p1 | (A)10 | 10 | 62728 | 62737 | LSC | *psbF* |
| *Phaius delavayi* | 28 | p1 | (A)11 | 11 | 63102 | 63112 | LSC | IGS (*psbE_petL*) |
| *Phaius delavayi* | 29 | p4 | (ATTA)3 | 12 | 65676 | 65687 | LSC | IGS (*psaJ_rpl33*) |
| *Phaius delavayi* | 30 | p2 | (TG)5 | 10 | 66181 | 66190 | LSC | IGS (*rpl33_rps18*) |
| *Phaius delavayi* | 31 | p4 | (ATAA)3 | 12 | 69402 | 69413 | LSC | *clpP* |
| *Phaius delavayi* | 32 | p1 | (T)10 | 10 | 69638 | 69647 | LSC | *clpP* |
| *Phaius delavayi* | 33 | p1 | (T)10 | 10 | 69892 | 69901 | LSC | *clpP* |
| *Phaius delavayi* | 34 | p1 | (T)10 | 10 | 72907 | 72916 | LSC | IGS (*psbB-psbT*) |
| *Phaius delavayi* | 35 | p1 | (T)14 | 14 | 75899 | 75912 | LSC | *petD* |
| *Phaius delavayi* | 36 | p1 | (A)10 | 10 | 77131 | 77140 | LSC | IGS (*petD_rpoA*) |
| *Phaius delavayi* | 37 | p2 | (TA)7 | 14 | 80045 | 80058 | LSC | IGS (*rps8_rpl14*) |
| *Phaius delavayi* | 38 | p2 | (GA)5 | 10 | 89425 | 89434 | LSC | *ycf2* |
| *Phaius delavayi* | 39 | p5 | (TATTA)4 | 20 | 98816 | 98835 | LSC | IGS (*rps12_trnV-GAC*) |
| *Phaius delavayi* | 40 | p1 | (T)11 | 11 | 99452 | 99462 | LSC | IGS (*rps12_trnV-GAC*) |
| *Phaius delavayi* | 41 | p1 | (T)12 | 12 | 109689 | 109700 | LSC | *ycf1* |
| *Phaius delavayi* | 42 | p1 | (A)10 | 10 | 109852 | 109861 | LSC | *ycf1* |
| *Phaius delavayi* | 43 | p1 | (T)10 | 10 | 111281 | 111290 | LSC | *ycf1* |
| *Phaius delavayi* | 44 | p1 | (A)10 | 10 | 114031 | 114040 | IR | *ycf1* |
| *Phaius delavayi* | 45 | p1 | (T)10 | 10 | 116609 | 116618 | IR | *ndhA* |
| *Phaius delavayi* | 46 | p6 | (CATATG)3 | 18 | 117193 | 117210 | LSC | *ndhA* |
| *Phaius delavayi* | 47 | p1 | (T)10 | 10 | 118740 | 118749 | LSC | IGS (*ndhI_nad6*) |
| *Phaius delavayi* | 48 | p1 | (T)10 | 10 | 118974 | 118983 | LSC | IGS (*nad6_ndhE*) |
| *Phaius delavayi* | 49 | p4 | (ATCA)3 | 12 | 119414 | 119425 | LSC | *ndhE* |
| *Phaius delavayi* | 50 | p3 | (TTA)4 | 12 | 119572 | 119583 | LSC | IGS (*ndhE_psaC*) |
| *Phaius delavayi* | 51 | p1 | (A)11 | 11 | 121976 | 121986 | LSC | IGS (*ndhD-ccsA*) |
| *Phaius delavayi* | 52 | p2 | (TA)5 | 10 | 124667 | 124676 | LSC | IGS (*rpl32_trnN-GUU*) |
| *Phaius delavayi* | 53 | p1 | (A)11 | 11 | 134008 | 134018 | IR | IGS (*trnV-GAC_rps12*) |
| *Phaius delavayi* | 54 | p5 | (ATAAT)4 | 20 | 134634 | 134653 | IR | IGS (*trnV-GAC_rps12*) |
| *Phaius delavayi* | 55 | p2 | (TC)5 | 10 | 144036 | 144045 | IR | *ycf12* |

SSR for *Phaius flavus*

| **ID** | **SSR nr.** | **SSR type** | **SSR** | **size** | **start** | **end** | **Region** | **Gene Region** |
| --- | --- | --- | --- | --- | --- | --- | --- | --- |
| *Phaius flavus* | 1 | p1 | (T)10 | 10 | 1405 | 1414 | LSC | IGS (*psbA_matK*) |
| *Phaius flavus* | 2 | p3 | (ATA)4 | 12 | 3458 | 3469 | LSC | IGS (*matK_rps16*) |
| *Phaius flavus* | 3 | p4 | (TCTA)3 | 12 | 5296 | 5307 | LSC | *rps16* |
| *Phaius flavus* | 4 | p5 | (ATCTT)3 | 15 | 8369 | 8383 | LSC | IGS (*psbK_psbI*) |
| *Phaius flavus* | 5 | p4 | (GTCT)3 | 12 | 12014 | 12025 | LSC | *atpA* |
| *Phaius flavus* | 6 | p1 | (A)10 | 10 | 13316 | 13325 | LSC | *atpF* |
| *Phaius flavus* | 7 | p1 | (T)10 | 10 | 17003 | 17012 | LSC | IGS (*rps2_rpoC2*) |
| *Phaius flavus* | 8 | c | (T)10 | 117 | 19107 | 19223 | LSC | *rpoC2* |
| *Phaius flavus* | 9 | p2 | (AT)5 | 10 | 20592 | 20601 | LSC | *rpoC2* |
| *Phaius flavus* | 10 | p2 | (AT)6 | 12 | 28908 | 28919 | LSC | IGS (*rpoB_trnC-GCA*) |
| *Phaius flavus* | 11 | p4 | (AGAA)3 | 12 | 31064 | 31075 | LSC | IGS (*psbM_trnD-GUC*) |
| *Phaius flavus* | 12 | p1 | (A)10 | 10 | 33060 | 33069 | LSC | IGS (*trnE-UUC_trnT-GGU*) |
| *Phaius flavus* | 13 | p2 | (AT)5 | 10 | 47555 | 47564 | LSC | IGS (*trnT-UGU _ trnL-UAA*) |
| *Phaius flavus* | 14 | p2 | (AT)8 | 16 | 48789 | 48804 | LSC | IGS (*trnL-UAA_trnF-GAA*) |
| *Phaius flavus* | 15 | p1 | (T)10 | 10 | 50944 | 50953 | LSC | *ndhK* |
| *Phaius flavus* | 16 | p1 | (A)10 | 10 | 51887 | 51896 | LSC | IGS (*ndhC_trnV-UAC*) |
| *Phaius flavus* | 17 | p1 | (A)10 | 10 | 52569 | 52578 | LSC | IGS (*ndhC_trnV-UAC*) |
| *Phaius flavus* | 18 | p1 | (T)10 | 10 | 55965 | 55974 | LSC | IGS (*atpB_rbcL*) |
| *Phaius flavus* | 19 | p1 | (A)11 | 11 | 60453 | 60463 | LSC | IGS (*accD_psaI*) |
| *Phaius flavus* | 20 | p4 | (AATG)3 | 12 | 63790 | 63801 | LSC | *cemA* |
| *Phaius flavus* | 21 | p1 | (T)11 | 11 | 65017 | 65027 | LSC | IGS (*petA_psbJ*) |
| *Phaius flavus* | 22 | p1 | (T)11 | 11 | 65358 | 65368 | LSC | IGS (*petA_psbJ*) |
| *Phaius flavus* | 23 | p1 | (A)11 | 11 | 67348 | 67358 | LSC | IGS (*psbE_petL*) |
| *Phaius flavus* | 24 | p1 | (T)12 | 12 | 67627 | 67638 | LSC | IGS (*psbE_petL*) |
| *Phaius flavus* | 25 | p1 | (A)11 | 11 | 69073 | 69083 | LSC | IGS (*psaJ_rpl33*) |
| *Phaius flavus* | 26 | p4 | (ATTA)3 | 12 | 69254 | 69265 | LSC | IGS (*psaJ_rpl33*) |
| *Phaius flavus* | 27 | p1 | (T)10 | 10 | 69466 | 69475 | LSC | IGS (*psaJ_rpl33*) |
| *Phaius flavus* | 28 | p2 | (TG)5 | 10 | 69765 | 69774 | LSC | IGS (*rpl33_rps18*) |
| *Phaius flavus* | 29 | p1 | (T)10 | 10 | 73227 | 73236 | LSC | *clpP* |
| *Phaius flavus* | 30 | p2 | (AT)8 | 16 | 73990 | 74005 | LSC | *clpP* |
| *Phaius flavus* | 31 | p2 | (TA)5 | 10 | 74310 | 74319 | LSC | IGS (*clpP_psbB*) |
| *Phaius flavus* | 32 | p4 | (TAGA)3 | 12 | 74510 | 74521 | LSC | IGS (*clpP_psbB*) |
| *Phaius flavus* | 33 | c | (T)10 | 69 | 76616 | 76684 | LSC | IGS (*psbB_psbT*) |
| *Phaius flavus* | 34 | p2 | (CT)5 | 10 | 78115 | 78124 | LSC | *petB* |
| *Phaius flavus* | 35 | p1 | (A)11 | 11 | 78290 | 78300 | LSC | *petB* |
| *Phaius flavus* | 36 | p1 | (T)14 | 14 | 79681 | 79694 | LSC | *petD* |
| *Phaius flavus* | 37 | c | (AAT)4 | 109 | 84855 | 84963 | LSC | *rpl36* |
| *Phaius flavus* | 38 | p1 | (T)11 | 11 | 85775 | 85785 | LSC | *rpl36* |
| *Phaius flavus* | 39 | p2 | (GA)5 | 10 | 93296 | 93305 | IR | *ycf2* |
| *Phaius flavus* | 40 | p1 | (T)12 | 12 | 103602 | 103613 | IR | IGS (*rps12_trnV-GAC*) |
| *Phaius flavus* | 41 | p1 | (G)10 | 10 | 106176 | 106185 | IR | *trnL-GAC* |
| *Phaius flavus* | 42 | c | (T)10 | 61 | 116524 | 116584 | LSC | IGS (*ndhF_rpl32*) |
| *Phaius flavus* | 43 | p1 | (T)12 | 12 | 116712 | 116723 | LSC | IGS (*ndhF_rpl32*) |
| *Phaius flavus* | 44 | p1 | (T)10 | 10 | 119064 | 119073 | LSC | IGS (*ccsA_ndhD*) |
| *Phaius flavus* | 45 | p3 | (AAT)4 | 12 | 121518 | 121529 | LSC | IGS (*psaC_ndhE*) |
| *Phaius flavus* | 46 | p4 | (TTGA)3 | 12 | 121671 | 121682 | LSC | *ndhE* |
| *Phaius flavus* | 47 | p1 | (A)10 | 10 | 125011 | 125020 | LSC | *ndhA* |
| *Phaius flavus* | 48 | p1 | (T)10 | 10 | 128112 | 128121 | LSC | *ycf1* |
| *Phaius flavus* | 49 | p1 | (T)10 | 10 | 130344 | 130353 | LSC | *ycf1* |
| *Phaius flavus* | 50 | p1 | (A)10 | 10 | 130461 | 130470 | LSC | *ycf1* |
| *Phaius flavus* | 51 | p1 | (T)14 | 14 | 131769 | 131782 | LSC | *ycf1* |
| *Phaius flavus* | 52 | p1 | (T)10 | 10 | 131890 | 131899 | LSC | *ycf1* |
| *Phaius flavus* | 53 | p1 | (A)12 | 12 | 132051 | 132062 | LSC | *ycf1* |
| *Phaius flavus* | 54 | p1 | (C)10 | 10 | 139588 | 139597 | IR | *trnI-GAU* |
| *Phaius flavus* | 55 | p1 | (A)12 | 12 | 142160 | 142171 | IR | IGS (*trnV-GAC_rps12*) |
| *Phaius flavus* | 56 | p2 | (TC)5 | 10 | 152468 | 152477 | IR | *ycf2* |
