# Supplementary material for: Plastome structure of 8 Calanthe s.l. species (Orchidaceae): comparative genomics, phylogenetic analysis
Source: BMC Plant Biol. 2022 Aug 3;22:387. doi: 10.1186/s12870-022-03736-0 (PMC9347164; doi:10.1186/s12870-022-03736-0)
Supplement: Supplementary file 7 — Additional file 7. [file 12870_2022_3736_MOESM7_ESM.docx]

**Kruskal-Wallis ANOVA Statistical test on the significance difference of SSRs across the 8 *Calanthe* group species**

*Notes*

| X-Function | Kruskal-Wallis ANOVA |
| --- | --- |
| User Name | Administrator |
| Time | 5/10/2022 10:22:25 |
| Data Filter | No |

*Input Data*

|  | Data | Range |
| --- | --- | --- |
| Group Range | [Book1]Sheet1!A"Plant Species" | [1*:8*] |
| Data Range | [Book1]Sheet1!B"SSC" | [1*:8*] |

*Descriptive Statistics*

|  |  | N | Min | Q1 | Median | Q3 | Max |
| --- | --- | --- | --- | --- | --- | --- | --- |
| "SSC" | *Calanthe alpina* | 1 | 14 | 14 | 14 | 14 | 14 |
|  | *Calanthe brevicornu* | 1 | 13 | 13 | 13 | 13 | 13 |
|  | *Calanthe ecarinata* | 1 | 13 | 13 | 13 | 13 | 13 |
|  | *Calanthe nipponica* | 1 | 15 | 15 | 15 | 15 | 15 |
|  | *Calanthe taibaishanensis* | 1 | 11 | 11 | 11 | 11 | 11 |
|  | *Calanthe tricarinata* | 1 | 11 | 11 | 11 | 11 | 11 |
|  | *Phaius delavayi* | 1 | 12 | 12 | 12 | 12 | 12 |
|  | *Phaius flavus* | 1 | 11 | 11 | 11 | 11 | 11 |

*Ranks*

|  |  | N | Mean Rank | Sum Rank |
| --- | --- | --- | --- | --- |
| "SSC" | *Calanthe alpina* | 1 | 7 | 7 |
|  | *Calanthe brevicornu* | 1 | 5.5 | 5.5 |
|  | *Calanthe ecarinata* | 1 | 5.5 | 5.5 |
|  | *Calanthe nipponica* | 1 | 8 | 8 |
|  | *Calanthe taibaishanensis* | 1 | 2 | 2 |
|  | *Calanthe tricarinata* | 1 | 2 | 2 |
|  | *Phaius delavayi* | 1 | 4 | 4 |
|  | *Phaius flavus* | 1 | 2 | 2 |

*Test Statistics*

|  | Chi-Square | DF | Prob>Chi-Square |
| --- | --- | --- | --- |
| "SSC" | 7 | 7 | 0.42888 |
| Null Hypothesis: The samples come from the same population.  Alternative Hypothesis: The samples come from different populations. **"SSC": At the 0.05 level, the populations are NOT significantly different.** | | | |

*Dunn's Test*

|  |  | Mean Rank Diff | Z | Prob | Sig |
| --- | --- | --- | --- | --- | --- |
| "SSC" | *Calanthe alpina Calanthe brevicornu* | 1.5 | 0.44651 | 1 | 0 |
|  | *Calanthe alpina Calanthe ecarinata* | 1.5 | 0.44651 | 1 | 0 |
|  | *Calanthe alpina Calanthe nipponica* | -1 | -0.29767 | 1 | 0 |
|  | *Calanthe alpina Calanthe taibaishanensis* | 5 | 1.48835 | 1 | 0 |
|  | *Calanthe alpina Calanthe tricarinata* | 5 | 1.48835 | 1 | 0 |
|  | *Calanthe alpina Phaius delavayi* | 3 | 0.89301 | 1 | 0 |
|  | *Calanthe alpina Phaius flavus* | 5 | 1.48835 | 1 | 0 |
|  | *Calanthe brevicornu Calanthe ecarinata* | 0 | 0 | 1 | 0 |
|  | *Calanthe brevicornu Calanthe nipponica* | -2.5 | -0.74418 | 1 | 0 |
|  | *Calanthe brevicornu Calanthe taibaishanensis* | 3.5 | 1.04185 | 1 | 0 |
|  | *Calanthe brevicornu Calanthe tricarinata* | 3.5 | 1.04185 | 1 | 0 |
|  | *Calanthe brevicornu Phaius delavayi* | 1.5 | 0.44651 | 1 | 0 |
|  | *Calanthe brevicornu Phaius flavus* | 3.5 | 1.04185 | 1 | 0 |
|  | *Calanthe ecarinata Calanthe nipponica* | -2.5 | -0.74418 | 1 | 0 |
|  | *Calanthe ecarinata Calanthe taibaishanensis* | 3.5 | 1.04185 | 1 | 0 |
|  | *Calanthe ecarinata Calanthe tricarinata* | 3.5 | 1.04185 | 1 | 0 |
|  | *Calanthe ecarinata Phaius delavayi* | 1.5 | 0.44651 | 1 | 0 |
|  | *Calanthe ecarinata Phaius flavus* | 3.5 | 1.04185 | 1 | 0 |
|  | *Calanthe nipponica Calanthe taibaishanensis* | 6 | 1.78602 | 1 | 0 |
|  | *Calanthe nipponica Calanthe tricarinata* | 6 | 1.78602 | 1 | 0 |
|  | *Calanthe nipponica Phaius delavayi* | 4 | 1.19068 | 1 | 0 |
|  | *Calanthe nipponica Phaius flavus* | 6 | 1.78602 | 1 | 0 |
|  | *Calanthe taibaishanensis Calanthe tricarinata* | 0 | 0 | 1 | 0 |
|  | *Calanthe taibaishanensis Phaius delavayi* | -2 | -0.59534 | 1 | 0 |
|  | *Calanthe taibaishanensis Phaius flavus* | 0 | 0 | 1 | 0 |
|  | *Calanthe tricarinata Phaius delavayi* | -2 | -0.59534 | 1 | 0 |
|  | *Calanthe tricarinata Phaius flavus* | 0 | 0 | 1 | 0 |
|  | *Phaius delavayi Phaius flavus* | 2 | 0.59534 | 1 | 0 |
| Sig equals 1 indicates that the difference of the means is significant at the 0.05 level.  Sig equals 0 indicates that the difference of the means is NOT significant at the 0.05 level. | | | | | |

*Notes*

| X-Function | Kruskal-Wallis ANOVA |
| --- | --- |
| User Name | Administrator |
| Time | 5/10/2022 10:31:18 |
| Data Filter | No |

*Input Data*

|  | Data | Range |
| --- | --- | --- |
| Group Range | [Book1]Sheet2!A"Plant species" | [1*:8*] |
| Data Range | [Book1]Sheet2!B"LSC" | [1*:8*] |

*Descriptive Statistics*

|  |  | N | Min | Q1 | Median | Q3 | Max |
| --- | --- | --- | --- | --- | --- | --- | --- |
| "LSC" | *Calanthe alpina* | 1 | 58 | 58 | 58 | 58 | 58 |
|  | *Calanthe brevicornu* | 1 | 51 | 51 | 51 | 51 | 51 |
|  | *Calanthe ecarinata* | 1 | 42 | 42 | 42 | 42 | 42 |
|  | *Calanthe nipponica* | 1 | 46 | 46 | 46 | 46 | 46 |
|  | *Calanthe taibaishanensis* | 1 | 51 | 51 | 51 | 51 | 51 |
|  | *Calanthe tricarinata* | 1 | 51 | 51 | 51 | 51 | 51 |
|  | *Phaius delavayi* | 1 | 44 | 44 | 44 | 44 | 44 |
|  | *Phaius flavus* | 1 | 42 | 42 | 42 | 42 | 42 |

*Ranks*

|  |  | N | Mean Rank | Sum Rank |
| --- | --- | --- | --- | --- |
| "LSC" | *Calanthe alpina* | 1 | 8 | 8 |
|  | *Calanthe brevicornu* | 1 | 6 | 6 |
|  | *Calanthe ecarinata* | 1 | 1.5 | 1.5 |
|  | *Calanthe nipponica* | 1 | 4 | 4 |
|  | *Calanthe taibaishanensis* | 1 | 6 | 6 |
|  | *Calanthe tricarinata* | 1 | 6 | 6 |
|  | *Phaius delavayi* | 1 | 3 | 3 |
|  | *Phaius flavus* | 1 | 1.5 | 1.5 |

*Test Statistics*

|  | Chi-Square | DF | Prob>Chi-Square |
| --- | --- | --- | --- |
| "LSC" | 7 | 7 | 0.42888 |
| Null Hypothesis: The samples come from the same population.  Alternative Hypothesis: The samples come from different populations. **"LSC": At the 0.05 level, the populations are NOT significantly different.** | | | |

*Dunn's Test*

|  |  | Mean Rank Diff | Z | Prob | Sig |
| --- | --- | --- | --- | --- | --- |
| "LSC" | *Calanthe alpina Calanthe brevicornu* | 2 | 0.59534 | 1 | 0 |
|  | *Calanthe alpina Calanthe ecarinata* | 6.5 | 1.93486 | 1 | 0 |
|  | *Calanthe alpina Calanthe nipponica* | 4 | 1.19068 | 1 | 0 |
|  | *Calanthe alpina Calanthe taibaishanensis* | 2 | 0.59534 | 1 | 0 |
|  | *Calanthe alpina Calanthe tricarinata* | 2 | 0.59534 | 1 | 0 |
|  | *Calanthe alpina Phaius delavayi* | 5 | 1.48835 | 1 | 0 |
|  | *Calanthe alpina Phaius flavus* | 6.5 | 1.93486 | 1 | 0 |
|  | *Calanthe brevicornu Calanthe ecarinata* | 4.5 | 1.33952 | 1 | 0 |
|  | *Calanthe brevicornu Calanthe nipponica* | 2 | 0.59534 | 1 | 0 |
|  | *Calanthe brevicornu Calanthe taibaishanensis* | 0 | 0 | 1 | 0 |
|  | *Calanthe brevicornu Calanthe tricarinata* | 0 | 0 | 1 | 0 |
|  | *Calanthe brevicornu Phaius delavayi* | 3 | 0.89301 | 1 | 0 |
|  | *Calanthe brevicornu Phaius flavus* | 4.5 | 1.33952 | 1 | 0 |
|  | *Calanthe ecarinata Calanthe nipponica* | -2.5 | -0.74418 | 1 | 0 |
|  | *Calanthe ecarinata Calanthe taibaishanensis* | -4.5 | -1.33952 | 1 | 0 |
|  | *Calanthe ecarinata Calanthe tricarinata* | -4.5 | -1.33952 | 1 | 0 |
|  | *Calanthe ecarinata Phaius delavayi* | -1.5 | -0.44651 | 1 | 0 |
|  | *Calanthe ecarinata Phaius flavus* | 0 | 0 | 1 | 0 |
|  | *Calanthe nipponica Calanthe taibaishanensis* | -2 | -0.59534 | 1 | 0 |
|  | *Calanthe nipponica Calanthe tricarinata* | -2 | -0.59534 | 1 | 0 |
|  | *Calanthe nipponica Phaius delavayi* | 1 | 0.29767 | 1 | 0 |
|  | *Calanthe nipponica Phaius flavus* | 2.5 | 0.74418 | 1 | 0 |
|  | *Calanthe taibaishanensis Calanthe tricarinata* | 0 | 0 | 1 | 0 |
|  | *Calanthe taibaishanensis Phaius delavayi* | 3 | 0.89301 | 1 | 0 |
|  | *Calanthe taibaishanensis Phaius flavus* | 4.5 | 1.33952 | 1 | 0 |
|  | *Calanthe tricarinata Phaius delavayi* | 3 | 0.89301 | 1 | 0 |
|  | *Calanthe tricarinata Phaius flavus* | 4.5 | 1.33952 | 1 | 0 |
|  | *Phaius delavayi Phaius flavus* | 1.5 | 0.44651 | 1 | 0 |
| Sig equals 1 indicates that the difference of the means is significant at the 0.05 level.  Sig equals 0 indicates that the difference of the means is NOT significant at the 0.05 level. | | | | | |

*Notes*

| X-Function | Kruskal-Wallis ANOVA |
| --- | --- |
| User Name | Administrator |
| Time | 5/10/2022 10:34:09 |
| Data Filter | No |

*Input Data*

|  | Data | Range |
| --- | --- | --- |
| Group Range | [Book1]Sheet3!A"Plant species" | [1*:8*] |
| Data Range | [Book1]Sheet3!B"IR" | [1*:8*] |

*Descriptive Statistics*

|  |  | N | Min | Q1 | Median | Q3 | Max |
| --- | --- | --- | --- | --- | --- | --- | --- |
| "IR" | *Calanthe alpina* | 1 | 2 | 2 | 2 | 2 | 2 |
|  | *Calanthe brevicornu* | 1 | 2 | 2 | 2 | 2 | 2 |
|  | *Calanthe ecarinata* | 1 | 2 | 2 | 2 | 2 | 2 |
|  | *Calanthe nipponica* | 1 | 2 | 2 | 2 | 2 | 2 |
|  | *Calanthe taibaishanensis* | 1 | 2 | 2 | 2 | 2 | 2 |
|  | *Calanthe tricarinata* | 1 | 2 | 2 | 2 | 2 | 2 |
|  | *Phaius delavayi* | 1 | 2 | 2 | 2 | 2 | 2 |
|  | *Phaius flavus* | 1 | 4 | 4 | 4 | 4 | 4 |

*Ranks*

|  |  | N | Mean Rank | Sum Rank |
| --- | --- | --- | --- | --- |
| "IR" | *Calanthe alpina* | 1 | 4 | 4 |
|  | *Calanthe brevicornu* | 1 | 4 | 4 |
|  | *Calanthe ecarinata* | 1 | 4 | 4 |
|  | *Calanthe nipponica* | 1 | 4 | 4 |
|  | *Calanthe taibaishanensis* | 1 | 4 | 4 |
|  | *Calanthe tricarinata* | 1 | 4 | 4 |
|  | *Phaius delavayi* | 1 | 4 | 4 |
|  | *Phaius flavus* | 1 | 8 | 8 |

*Test Statistics*

|  | Chi-Square | DF | Prob>Chi-Square |
| --- | --- | --- | --- |
| "IR" | 7 | 7 | 0.42888 |
| Null Hypothesis: The samples come from the same population.  Alternative Hypothesis: The samples come from different populations. **"IR": At the 0.05 level, the populations are NOT significantly different.** | | | |

*Dunn's Test*

|  |  | Mean Rank Diff | Z | Prob | Sig |
| --- | --- | --- | --- | --- | --- |
| "IR" | *Calanthe alpina Calanthe brevicornu* | 0 | 0 | 1 | 0 |
|  | *Calanthe alpina Calanthe ecarinata* | 0 | 0 | 1 | 0 |
|  | *Calanthe alpina Calanthe nipponica* | 0 | 0 | 1 | 0 |
|  | *Calanthe alpina Calanthe taibaishanensis* | 0 | 0 | 1 | 0 |
|  | *Calanthe alpina Calanthe tricarinata* | 0 | 0 | 1 | 0 |
|  | *Calanthe alpina Phaius delavayi* | 0 | 0 | 1 | 0 |
|  | *Calanthe alpina Phaius flavus* | -4 | -2 | 1 | 0 |
|  | *Calanthe brevicornu Calanthe ecarinata* | 0 | 0 | 1 | 0 |
|  | *Calanthe brevicornu Calanthe nipponica* | 0 | 0 | 1 | 0 |
|  | *Calanthe brevicornu Calanthe taibaishanensis* | 0 | 0 | 1 | 0 |
|  | *Calanthe brevicornu Calanthe tricarinata* | 0 | 0 | 1 | 0 |
|  | *Calanthe brevicornu Phaius delavayi* | 0 | 0 | 1 | 0 |
|  | *Calanthe brevicornu Phaius flavus* | -4 | -2 | 1 | 0 |
|  | *Calanthe ecarinata Calanthe nipponica* | 0 | 0 | 1 | 0 |
|  | *Calanthe ecarinata Calanthe taibaishanensis* | 0 | 0 | 1 | 0 |
|  | *Calanthe ecarinata Calanthe tricarinata* | 0 | 0 | 1 | 0 |
|  | *Calanthe ecarinata Phaius delavayi* | 0 | 0 | 1 | 0 |
|  | *Calanthe ecarinata Phaius flavus* | -4 | -2 | 1 | 0 |
|  | *Calanthe nipponica Calanthe taibaishanensis* | 0 | 0 | 1 | 0 |
|  | *Calanthe nipponica Calanthe tricarinata* | 0 | 0 | 1 | 0 |
|  | *Calanthe nipponica Phaius delavayi* | 0 | 0 | 1 | 0 |
|  | *Calanthe nipponica Phaius flavus* | -4 | -2 | 1 | 0 |
|  | *Calanthe taibaishanensis Calanthe tricarinata* | 0 | 0 | 1 | 0 |
|  | *Calanthe taibaishanensis Phaius delavayi* | 0 | 0 | 1 | 0 |
|  | *Calanthe taibaishanensis Phaius flavus* | -4 | -2 | 1 | 0 |
|  | *Calanthe tricarinata Phaius delavayi* | 0 | 0 | 1 | 0 |
|  | *Calanthe tricarinata Phaius flavus* | -4 | -2 | 1 | 0 |
|  | *Phaius delavayi Phaius flavus* | -4 | -2 | 1 | 0 |
| Sig equals 1 indicates that the difference of the means is significant at the 0.05 level.  Sig equals 0 indicates that the difference of the means is NOT significant at the 0.05 level. | | | | | |

**Kruskal-Wallis statistical test on the significance difference of tandem repeats across the 8 *Calanthe* group species.**

*Notes*

| X-Function | Kruskal-Wallis ANOVA |
| --- | --- |
| User Name | Administrator |
| Time | 5/10/2022 12:24:30 |
| Data Filter | No |

*Input Data*

|  | Data | Range |
| --- | --- | --- |
| Group Range | [Book1]Sheet1!A"Plant species" | [1*:8*] |
| Data Range | [Book1]Sheet1!B"Tandem repeats" | [1*:8*] |

*Descriptive Statistics*

|  |  | N | Min | Q1 | Median | Q3 | Max |
| --- | --- | --- | --- | --- | --- | --- | --- |
| "Tandem repeats" | *Calanthe alpina* | 1 | 34 | 34 | 34 | 34 | 34 |
|  | *Calanthe brevicornu* | 1 | 38 | 38 | 38 | 38 | 38 |
|  | *Calanthe ecarinata* | 1 | 37 | 37 | 37 | 37 | 37 |
|  | *Calanthe nipponica* | 1 | 39 | 39 | 39 | 39 | 39 |
|  | *Calanthe taibaishanensis* | 1 | 30 | 30 | 30 | 30 | 30 |
|  | *Calanthe tricarinata* | 1 | 37 | 37 | 37 | 37 | 37 |
|  | *Phaius delavayi* | 1 | 40 | 40 | 40 | 40 | 40 |
|  | *Phaius flavus* | 1 | 28 | 28 | 28 | 28 | 28 |

*Ranks*

|  |  | N | Mean Rank | Sum Rank |
| --- | --- | --- | --- | --- |
| "Tandem repeats" | *Calanthe alpina* | 1 | 3 | 3 |
|  | *Calanthe brevicornu* | 1 | 6 | 6 |
|  | *Calanthe ecarinata* | 1 | 4.5 | 4.5 |
|  | *Calanthe nipponica* | 1 | 7 | 7 |
|  | *Calanthe taibaishanensis* | 1 | 2 | 2 |
|  | *Calanthe tricarinata* | 1 | 4.5 | 4.5 |
|  | *Phaius delavayi* | 1 | 8 | 8 |
|  | *Phaius flavus* | 1 | 1 | 1 |

*Test Statistics*

|  | Chi-Square | DF | Prob>Chi-Square |
| --- | --- | --- | --- |
| "Tandem repeats" | 7 | 7 | 0.42888 |
| Null Hypothesis: The samples come from the same population.  Alternative Hypothesis: The samples come from different populations. **"Tandem repeats": At the 0.05 level, the populations are NOT significantly different.** | | | |

*Dunn's Test*

|  |  | Mean Rank Diff | Z | Prob | Sig |
| --- | --- | --- | --- | --- | --- |
| "Tandem repeats" | *Calanthe alpina Calanthe brevicornu* | -3 | -0.87123 | 1 | 0 |
|  | *Calanthe alpina Calanthe ecarinata* | -1.5 | -0.43561 | 1 | 0 |
|  | *Calanthe alpina Calanthe nipponica* | -4 | -1.16164 | 1 | 0 |
|  | *Calanthe alpina Calanthe taibaishanensis* | 1 | 0.29041 | 1 | 0 |
|  | *Calanthe alpina Calanthe tricarinata* | -1.5 | -0.43561 | 1 | 0 |
|  | *Calanthe alpina Phaius delavayi* | -5 | -1.45204 | 1 | 0 |
|  | *Calanthe alpina Phaius flavus* | 2 | 0.58082 | 1 | 0 |
|  | *Calanthe brevicornu Calanthe ecarinata* | 1.5 | 0.43561 | 1 | 0 |
|  | *Calanthe brevicornu Calanthe nipponica* | -1 | -0.29041 | 1 | 0 |
|  | *Calanthe brevicornu Calanthe taibaishanensis* | 4 | 1.16164 | 1 | 0 |
|  | *Calanthe brevicornu Calanthe tricarinata* | 1.5 | 0.43561 | 1 | 0 |
|  | *Calanthe brevicornu Phaius delavayi* | -2 | -0.58082 | 1 | 0 |
|  | *Calanthe brevicornu Phaius flavus* | 5 | 1.45204 | 1 | 0 |
|  | *Calanthe ecarinata Calanthe nipponica* | -2.5 | -0.72602 | 1 | 0 |
|  | *Calanthe ecarinata Calanthe taibaishanensis* | 2.5 | 0.72602 | 1 | 0 |
|  | *Calanthe ecarinata Calanthe tricarinata* | 0 | 0 | 1 | 0 |
|  | *Calanthe ecarinata Phaius delavayi* | -3.5 | -1.01643 | 1 | 0 |
|  | *Calanthe ecarinata Phaius flavus* | 3.5 | 1.01643 | 1 | 0 |
|  | *Calanthe nipponica Calanthe taibaishanensis* | 5 | 1.45204 | 1 | 0 |
|  | *Calanthe nipponica Calanthe tricarinata* | 2.5 | 0.72602 | 1 | 0 |
|  | *Calanthe nipponica Phaius delavayi* | -1 | -0.29041 | 1 | 0 |
|  | *Calanthe nipponica Phaius flavus* | 6 | 1.74245 | 1 | 0 |
|  | *Calanthe taibaishanensis Calanthe tricarinata* | -2.5 | -0.72602 | 1 | 0 |
|  | *Calanthe taibaishanensis Phaius delavayi* | -6 | -1.74245 | 1 | 0 |
|  | *Calanthe taibaishanensis Phaius flavus* | 1 | 0.29041 | 1 | 0 |
|  | *Calanthe tricarinata Phaius delavayi* | -3.5 | -1.01643 | 1 | 0 |
|  | *Calanthe tricarinata Phaius flavus* | 3.5 | 1.01643 | 1 | 0 |
|  | *Phaius delavayi Phaius flavus* | 7 | 2.03286 | 1 | 0 |
| Sig equals 1 indicates that the difference of the means is significant at the 0.05 level.  Sig equals 0 indicates that the difference of the means is NOT significant at the 0.05 level. | | | | | |
