# Supplementary material for: Plastome structure of 8 Calanthe s.l. species (Orchidaceae): comparative genomics, phylogenetic analysis
Source: BMC Plant Biol. 2022 Aug 3;22:387. doi: 10.1186/s12870-022-03736-0 (PMC9347164; doi:10.1186/s12870-022-03736-0)
Supplement: Supplementary file 9 — Additional file 9. [file 12870_2022_3736_MOESM9_ESM.pdf]

Kruskal-Wallis ANOVA statistical test on the significance difference of RSCU across the 8 *Calanthe* group plastomes.

Notes

X-Function	Kruskal-Wallis ANOVA
User Name	Administrator
Time	5/10/2022 21:52:36
Data Filter	No

Input Data

	Data	Range
Group Range	[Book1]Sheet2!A"Plant species"	[1*:168*]
Data Range	[Book1]Sheet2!B"RSCUs"	[1*:168*]

Descriptive Statistics

		N	Min	Q1	Median	Q3	Max
"RSCUs"	*C. alpina*	21	1	2	2	4	6
	C. brevicornu	21	1	2	2.03	4.005	6
	C. tricarinata	21	1	2	2	4	6.01
	P. delavayi	21	1	2	2	4	6
	P. flavus	21	1	2	2	4	6.02
	C. nipponica	21	1	2	2	4	6.01
	C. taibaishanensis	21	1	2	2	4.005	6.01
	C. ecarinata	21	1	2	2	4	7

Ranks

		N	Mean Rank	Sum Rank
"RSCUs"	*C. alpina*	21	80.2619	1685.5
	C. brevicornu	21	87.35714	1834.5
	C. tricarinata	21	85.21429	1789.5
	P. delavayi	21	83.2381	1748
	P. flavus	21	85.21429	1789.5
	C. nipponica	21	85.5	1795.5
	C. taibaishanensis	21	86.21429	1810.5
	C. ecarinata	21	83	1743

Test Statistics

	Chi-Square	DF	Prob>Chi-Square
"RSCUs"	0.3353	7	0.99985

Null Hypothesis:The samples come from the same population.
Alternative Hypothesis:The samples come from different populations.
"RSCUs": At the 0.05 level, the populations are NOT significantly different.

Dunn's Test

		Mean Rank Diff	Z	Prob	Sig

"RSCUs"	*C. alpina C. brevicornu*	-7.09524	-0.49158	1	0
	C. alpina C. tricarinata	-4.95238	-0.34311	1	0
	C. alpina P. delavayi	-2.97619	-0.2062	1	0
	C. alpina P. flavus	-4.95238	-0.34311	1	0
	C. alpina C. nipponica	-5.2381	-0.36291	1	0
	C. alpina C. taibaishanensis	-5.95238	-0.4124	1	0
	C. alpina C. ecarinata	-2.7381	-0.1897	1	0
	C. brevicornu C. tricarinata	2.14286	0.14846	1	0
	C. brevicornu P. delavayi	4.11905	0.28538	1	0
	C. brevicornu P. flavus	2.14286	0.14846	1	0
	C. brevicornu C. nipponica	1.85714	0.12867	1	0
	C. brevicornu C. taibaishanensis	1.14286	0.07918	1	0
	C. brevicornu C. ecarinata	4.35714	0.30187	1	0
	C. tricarinata P. delavayi	1.97619	0.13692	1	0
	C. tricarinata P. flavus	0	0	1	0
	C. tricarinata C. nipponica	-0.28571	-0.0198	1	0
	C. tricarinata C. taibaishanensis	-1	-0.06928	1	0
	C. tricarinata C. ecarinata	2.21429	0.15341	1	0
	P. delavayi P. flavus	-1.97619	-0.13692	1	0
	P. delavayi C. nipponica	-2.2619	-0.15671	1	0
	P. delavayi C. taibaishanensis	-2.97619	-0.2062	1	0
	P. delavayi C. ecarinata	0.2381	0.0165	1	0
	P. flavus C. nipponica	-0.28571	-0.0198	1	0
	P. flavus C. taibaishanensis	-1	-0.06928	1	0
	P. flavus C. ecarinata	2.21429	0.15341	1	0
	C. nipponica C. taibaishanensis	-0.71429	-0.04949	1	0
	C. nipponica C. ecarinata	2.5	0.17321	1	0
	C. taibaishanensis C. ecarinata	3.21429	0.22269	1	0

Sig equals 1 indicates that the difference of the means is significant at the 0.05 level.
Sig equals 0 indicates that the difference of the means is NOT significant at the 0.05 level.
